# Demethylzeylasteral suppresses the expression of MESP1 by reducing H3K18la level to inhibit the malignant behaviors of pancreatic cancer

**DOI:** 10.1038/s41420-025-02603-9

**Published:** 2025-07-03

**Authors:** Xiaolei Ma, Mengxing Cheng, Yanxin Jia, Kun Zhang, Haocheng Zhang, Di Feng, Wenxiao Xu, Guofen Qiao

**Affiliations:** 1 Department of Pharmacology (State Key Laboratory of Frigid Zone Cardiovascular Diseases, State-Province Key Laboratories of Biomedicine-Pharmaceutics of China, Key Laboratory of Cardiovascular Research, Ministry of Education), College of Pharmacy, Harbin Medical University, Harbin, China; 2 Department of Orthopedics, The Second Affiliated Hospital of Harbin Medical University, Harbin, China; 3 Department of Pharmacy, The Sixth Affiliated Hospital of Harbin Medical University, Harbin, China; 4 Department of Pathology, Harbin Medical University Cancer Hospital, Harbin, China

**Keywords:** Targeted therapies, Acetylation

## Abstract

Glycolysis is a hallmark metabolic pathway in pancreatic cancer (PC). As the end product of glycolysis, lactic acid accumulates significantly in PC. Lactic acid serves as a primary substrate for histone lactylation, leading to an upregulation of histone lactylation levels, which likely contributes to progression of PC. This study reveals novel insights, highlighting that H3K18la levels are elevated in PC tissues and cells. Notably, the natural compound demethylzeylasteral (DML), derived from Tripterygium wilfordii Hook F (TwHF), substantially decreases lactic acid generation in PC cells, subsequently resulting in the downregulation of H3K18la levels and inhibiting the aggressive characteristics of PC cells. To further investigate the underlying mechanisms, we conducted RNA-seq analysis on DML-treated cells and ChIP-seq analyses for H3K18la. For the first time, mesoderm-related factor 1 (MESP1) was identified as a target protein modulated by both DML and H3K18la. DML was shown to repress the expression of MESP1, while sodium lactate (Nala) was found to partially restore its expression levels. Overexpression of MESP1 was linked to the promotion of epithelial-mesenchymal transition (EMT) and apoptosis in PC cells. Furthermore, RNA-seq analyses following MESP1 silencing indicated its significant association with critical physiological processes in PC cells, including the cell cycle, apoptosis, and cell adhesion. Importantly, MESP1 has also been connected to various cancer metabolism pathways, such as MAPK, PI3K-AKT, and carbon metabolism. This research is groundbreaking in demonstrating that DML impedes the malignant behavior of PC cells by downregulating H3K18la levels and diminishing the expression of the oncogene MESP1.

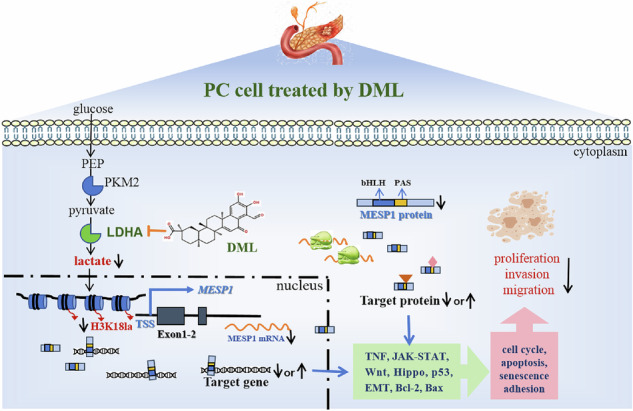

## Introduction

Pancreatic cancer (PC) is a highly malignant tumor and the seventh leading cause of cancer-related mortality globally. Recent years have witnessed a steady increase in the incidence of PC, and it is projected to become the second leading cause of cancer-related mortality in the United States by 2025 [1]. Over the past four decades, the survival rate of PC patients has remained largely unchanged. Currently, it is widely accepted that the occurrence of PC is predominantly driven by KRAS mutations, present in approximately 90% of PC patients. Besides KRAS, significant mutations have been identified in TP53, CDKN2A, and Smad4 in 80%, 26%, and 25% of patients, respectively [2–4]. Sequencing results from primary tumors and matched metastatic tissues revealed high concordance in driver gene mutations [5, 6], underscoring the significant role of non-hereditability, transcriptional reprogramming, and epigenetic plasticity in the metastatic cascade of PC. Therefore, elucidating the molecular mechanisms of epigenetics in PC progression is crucial for identifying novel diagnostic and therapeutic targets.

Histone lactylation, a novel epigenetic modification, was discovered in 2019. Core proteins of MCF-7 cells were analyzed using high-performance liquid chromatography-tandem mass spectrometry. A mass shift of 72.021 Da in three lysine residues of the proteolytic peptide segment corresponded to the addition of a lactate group to the ε-amino group of lysine residues. Further extensive mass spectrometry in HeLa cells and murine bone marrow-derived macrophages (BMDMs) identified 26 and 16 histone Kla sites, respectively [7, 8]. Lactate serves as the substrate for histone lactylation by providing lactate groups. A decrease in intracellular lactate levels will consequently reduce histone lactylation. LDHA (lactate dehydrogenase A) is the key enzyme catalyzing the conversion of pyruvate to lactate during anaerobic glycolysis, representing the primary catalytic enzyme for cytoplasmic lactate synthesis. Therefore, silencing LDHA will diminish cytoplasmic lactate production, reduce nuclear lactate availability, and ultimately inhibit histone lactylation [9]. Some studies have shown that the level of lactylation in gastrointestinal tumors cells is generally upregulated. Lactylation modification sites are widely distributed in cellular subcellular localization and have diverse functions, which are significantly enriched in a variety of important tumor-related biological processes.

Prior investigations have demonstrated that the glycolysis-H3K18la-TTK/BUB1B positive feedback loop promotes the malignant progression of PC, which is similar to the discovery of the NUSAP1-LDHA-glycolysis-lactate feed-forward loop [10, 11]. The elucidation of such positive feedback loops contributes to an in-depth exploration of the potential association between enhanced PC invasiveness and metabolic remodeling, and provides a novel perspective for understanding the mechanism of the Warburg effect in tumorigenesis. Furthermore, studies have also shown that lactylation modification affects PDAC resistance and is identified as a biomarker for poor PC prognosis [12, 13]. In summary, lactylation modification plays a key role in the development and therapeutic response of PC. However, the downstream mechanisms by which lactylation functions in PC and drugs that inhibit lactylation modification have not yet been thoroughly investigated, and further exploration is urgently needed.

Demethylzeylasteral (DML), a pentacyclic triterpenoid compound isolated from the xylem of Tripterygium wilfordii (TwHF) roots, has exhibited a broad spectrum of pharmacological properties and therapeutic effects since the 1990s. It modulates various signaling pathways, prevents disease progression, and exhibits significant anti-inflammatory [14], immunosuppressive [15], and anti-coronavirus effects [16]. DML demonstrates efficacy in treating various cancers, such as colorectal cancer, gastric cancer, chronic myelogenous leukemia, melanoma, and other malignancies [17]. Recent studies have also demonstrated that DML reduced lactate production, thereby inhibiting H3 histone lactylation levels (H3K9la and H3K56la), to counteract the progression of hepatocellular carcinoma [18]. However, limited research has been conducted to investigate the function and mechanism of DML in PC.

The MESP1 gene encodes an alkaline helix-loop-helix (bHLH) transcription factor, classified as bHLHC5 [19]. MESP1 acts as a master regulator of the cardiovascular system [19–23]. MESP1 has long been recognized for its pivotal role in embryonic cardiovascular development. However, recent reports indicate that abnormal expression of MESP1 promotes tumorigenesis [24]. Analysis of The Cancer Genome Atlas (TCGA) database reveals that high MESP1 expression is closely associated with the development of multiple malignancies. MESP1 not only promoted the proliferation of gastric cancer cells but also played a crucial role in the proliferation and survival of non-small cell lung cancer (NSCLC) and was associated with poor patient prognosis [24, 25]. However the expression of MESP1 in PC tissues and cells has not been reported, and its potential mechanisms remain unclear.

All in all, we observed elevated levels of Pan Kla and H3K18la in PC tissues and cells, which promoted the progression of PC. Our study represented the first demonstration that DML reduced H3K18la levels, thereby suppressing the expression of the oncogenic factor MESP1 and inhibiting the progression of PC. Furthermore, we have, for the first time, investigated the expression levels, function, and underlying mechanisms of MESP1 in PC, thus addressing a significant gap in the current literature. These results significantly contribute to the understanding of histone lactylation mechanisms in PC, providing novel insights into potential therapeutic targets and offering new theoretical support for the clinical application of DML in PC treatment.

## Results

### The level of histone lactylation was significantly increased in PC tissues and cell lines

**Fig. 1:**
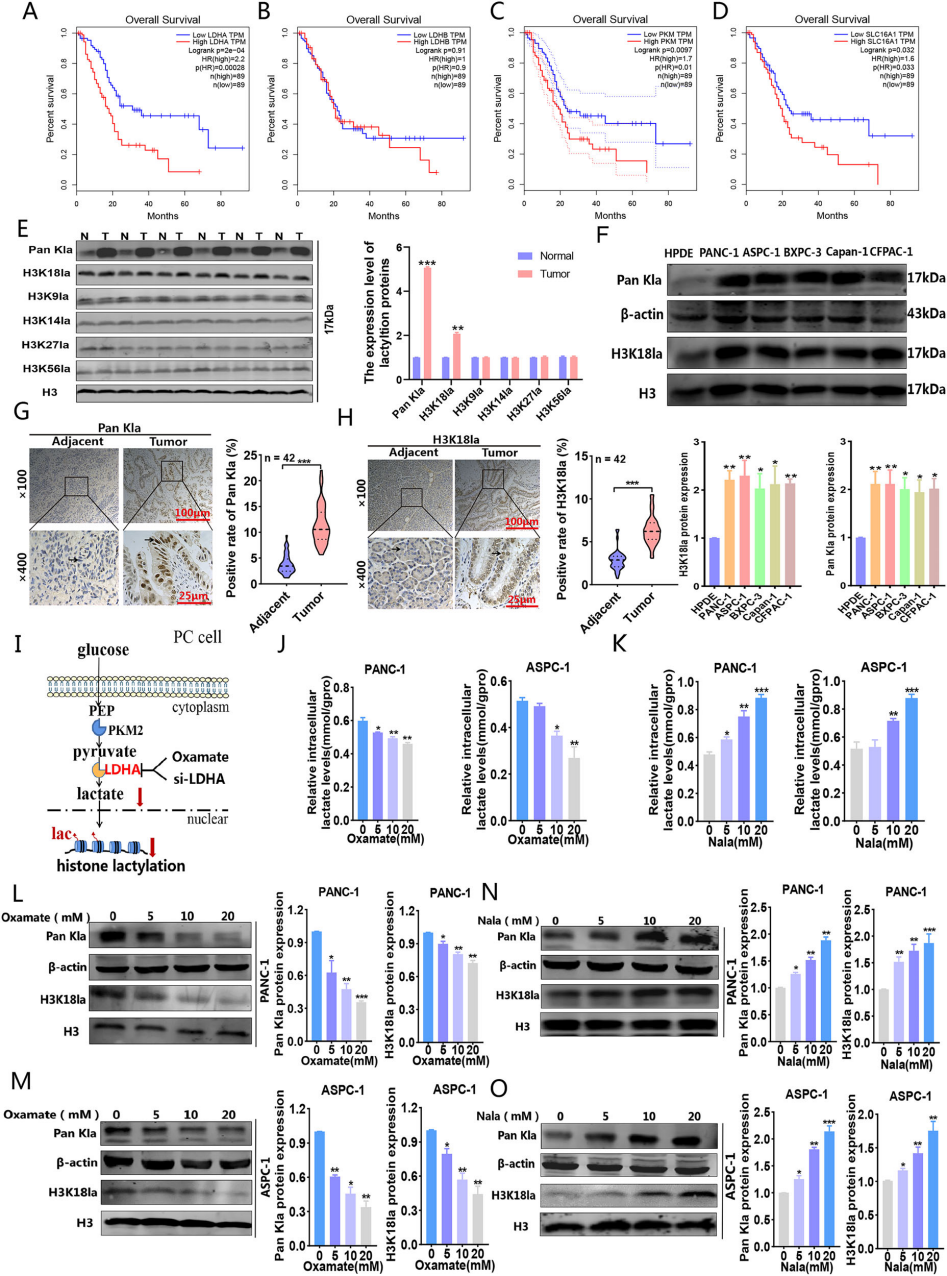
Exploration of the histone lactylation levels in PC. **A**–**D** The relationship between LDHA, LDHB, PKM and SLC16A1 and the overall survival rate of PC patients was analyzed by TCGA database (cancergeni.nih.gov). **E** The major type of 17 kDa histone lactylation in PC patients tissues was detected by western blot. **F** Western blot was used to investigate the level of H3K18la in various PC cell lines. **G**, **H** Lactylation levels were visualized by IHC in normal and tumor tissues. **I** Schematic diagram of histone lactylation inhibition methods target. **J**, **K** Intracellular lactate levels were measured from PANC-1 and ASPC-1 cells cultured in different concentrations of oxamate or Nala by a lactate colorimetric kit. **L**–**O** Lactylation and H3K18la levels were detected in PANC-1 and ASPC-1 cells cultured in different concentrations of Nala or oxamate by western blot. All data are shown as the mean ± SD of at least three independent experiments. *p < 0.05, **p < 0.01, ***p < 0.001.

Analysis of 89 high-expression samples and 89 low-expression samples of LDHA, LDHB, PKM, and SLC16A1 from the TCGA database revealed that elevated expression levels of these genes, which play a crucial role in lactate synthesis, were negatively correlated with the survival rate of PC patients (Fig. 1A–D). The activation of glycolysis leads to a significant buildup of lactic acid. Given lactic acid’s role as a substrate for histone lactylation, we proceeded to analyze the lactylation levels of histones in tissues from PC patients. Our western blot assays revealed that the predominant lactylation proteins band was located around 17 kDa. Subsequently, we examined all histone lactylation types at this molecular weight and observed a pronounced elevation in H3K18la (Fig. 1E). Comprehensive evaluation through western blotting demonstrated markedly increased level of Pan Kla and H3K18la across various PC cell lines (Fig. 1F). We procured 42 pairs of adjacent and tumor tissues from PC patients for immunohistochemical analysis. The expression levels of Pan Kla and H3K18la in tumor tissues were markedly elevated compared to their adjacent normal counterparts (Fig. 1G, H). In order to investigate the effects of histone lactylation on the progression of PC, we manipulated cellular lactate concentrations by applying Nala and inhibiting glycolysis, thereby adjusting the levels of Pan Kla and H3K18la (Fig. 1I, K). The findings revealed that oxamate decreased the intracellular lactate levels and lactylation in a dose-dependent manner (Fig. 1L, M), whereas Nala had the opposite effect, increasing both intracellular lactate and lactylation levels (Fig. 1N, O). These findings indicate that Pan Kla and H3K18la levels are elevated in PC tissues and cells and regulated by glycolysis.

### The decrease in histone lactylation modification suppressed the aggressive biological activities of PC cells

**Fig. 2:**
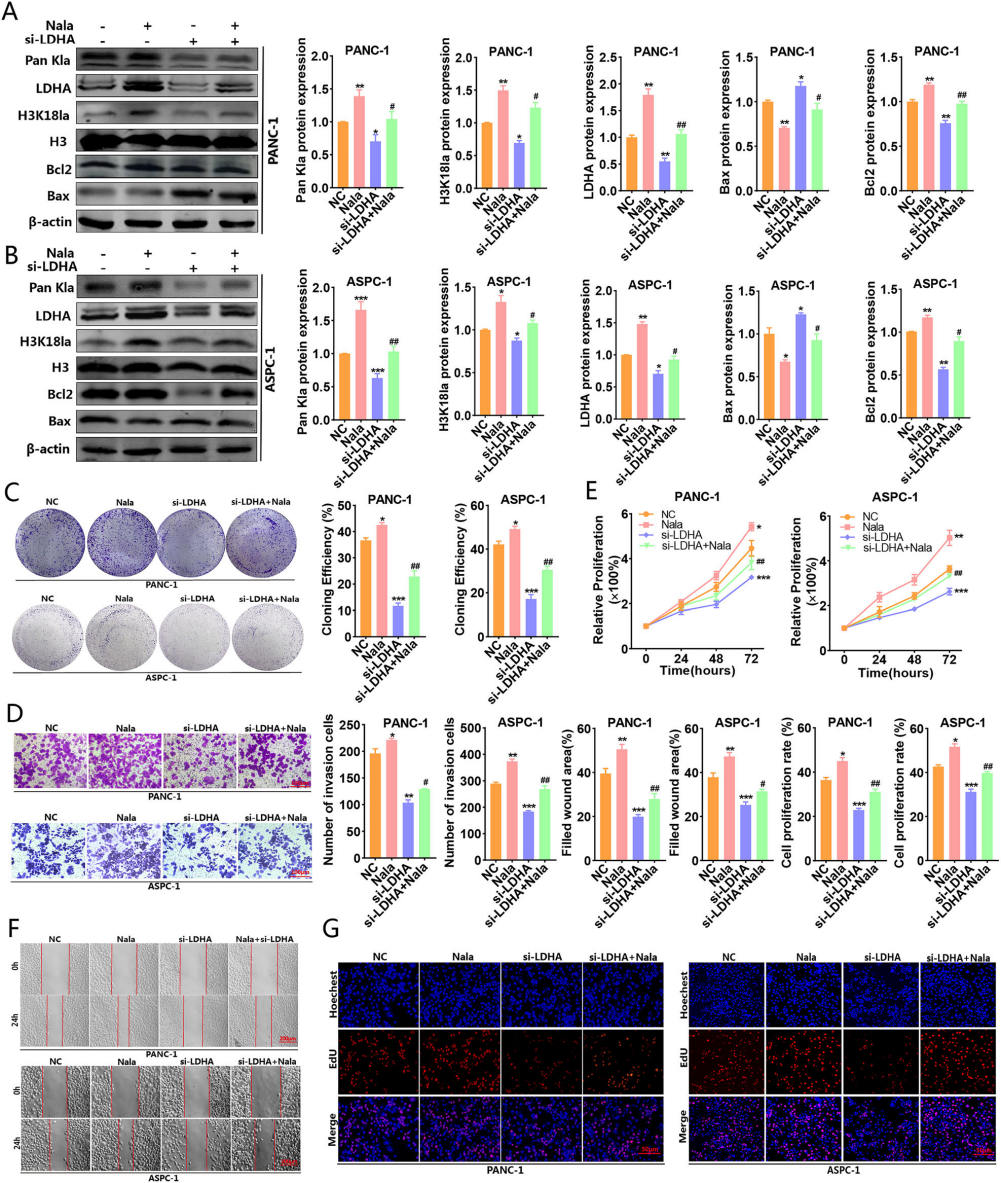
Functional experiments were performed to elucidate the involvement of histone lactylation in the malignant biological behavior of PC cells. **A**, **B** The expression levels of Pan Kla, H3K18la, Bax and Bcl2 in PANC-1 and ASPC-1 cells after LDHA silencing were detected by western blot. Colony formation (**C**) transwell invasion (**D**) CCK-8 (**E**) wound-healing (**F**) and EdU (**G**) assays were used to explore the effects of H3K18la inhibition on the proliferation, migration and invasion of PC cells. All data are shown as the mean ± SD of at least three independent experiments. *p < 0.05, **p < 0.01, ***p < 0.001, vs. NC. ^#^*p* < 0.05, ^##^*p* < 0.01, ^###^*p* < 0.001, vs. si-LDHA.

Given that oxamate might demonstrate biological activities beyond merely restraining lactate synthesis, we implemented LDHA silencing to diminish lactate levels, thereby lowering lactylation for further functional investigations. All experiments were carried out utilizing two PC cell lines, PANC-1 and ASPC-1. Initially, western blot analyses indicated that the silence of LDHA led to a decrease in H3K18la levels, whereas Nala was shown to alleviate the suppressive effect of si-LDHA, thereby partially reinstating the lactylation levels. We observed that the reduction of H3K18la levels led to an upregulation of Bax expression, a downregulation of Bcl2 expression, and an enhancement of apoptosis rate, while Nala exhibited a partial antagonistic effect against the pro-apoptotic response (Fig. 2A, B and Supplementary Fig. S1A, B). Functional assays revealed that lowering H3K18la levels impeded cellular aggregation, proliferation, invasion, and migration capabilities. Furthermore, Nala was shown to partially restore the biological functionalities of cancerous cells (Fig. 2C–G). These results suggest that decreasing H3K18la levels diminishes the malignant biological characteristics of PC cells.

### DML diminished histone lactylation by restricting lactate synthesis, thereby impairing the biological functions of PC cells

**Fig. 3:**
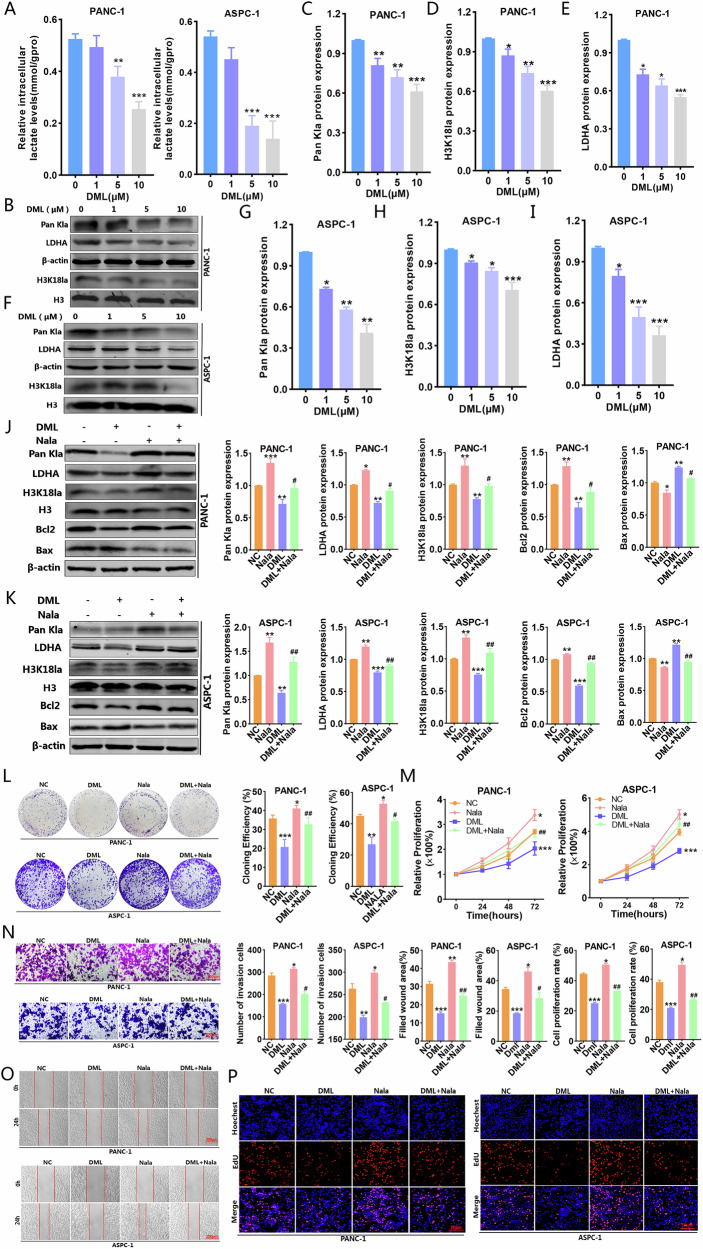
DML was involved in histone lactylation and inhibited the progression of PC. **A** Intracellular lactate levels were measured from PANC-1 and ASPC-1 cells cultured in different concentrations of DML by a lactate colorimetric kit. The expression levels of Pan Kla, H3K18la, and LDHA in PANC-1 (**B**–**E**) and ASPC-1 (**F**–**I**) cells treated by different concentrations of DML were detected by western blot. The rescue of Nala on the expression levels of Pan Kla, H3K18la, LDHA, Bax and Bcl2 in PANC-1 (**J**) and ASPC-1 (**K**) cells treated with DML was detected by western blot. **L**–**P** Colony formation (**L**) CCK-8 (**M**) transwell invasion (**N**) wound-healing (**O**) and EdU (**P**) assays were used to investigate the effect of DML on the proliferation, migration and invasion of PC cells by regulating H3K18la. All data are shown as the mean ± SD of at least three independent experiments. *p < 0.05, **p < 0.01, ***p < 0.001, vs. NC. ^#^*p* < 0.05, ^##^*p* < 0.01, ^###^*p* < 0.001, vs. DML.

Prior research has shown that DML suppresses lactate production and displays anti-cancer properties in liver cancer; however, its function in PC cells has not been thoroughly investigated. Lactic acid assays (Fig. 3A) and western blot (Fig. 3B–I) analyses demonstrated that DML significantly diminished LDHA expression, decreased lactic acid levels in a concentration-dependent manner, and lowered histone lactylation levels. Follow-up investigations revealed that DML enhanced Bax expression, reduced Bcl2 expression, and facilitated apoptosis rate, whereas Nala was able to partially counteract the inhibitory effects of DML on histone lactylation and attenuated its pro-apoptotic effect (Fig. 3J, K and Supplementary Fig. S1C, D). Functional assays revealed that DML significantly reduced the colony formation, proliferation, migration, and invasion abilities of PC cells, while Nala partially reinstated multiple malignant biological behaviors of cancer cells (Fig. 3L–P). These results indicate that DML reduced LDHA expression and lactic acid synthesis, thereby suppressing histone lactylation levels, inhibiting proliferation, migration, and invasion of PC cells while promoting apoptosis.

### MESP1 served as a downstream target of DML inhibiting PC progression by decreasing H3K18la level

**Fig. 4:**
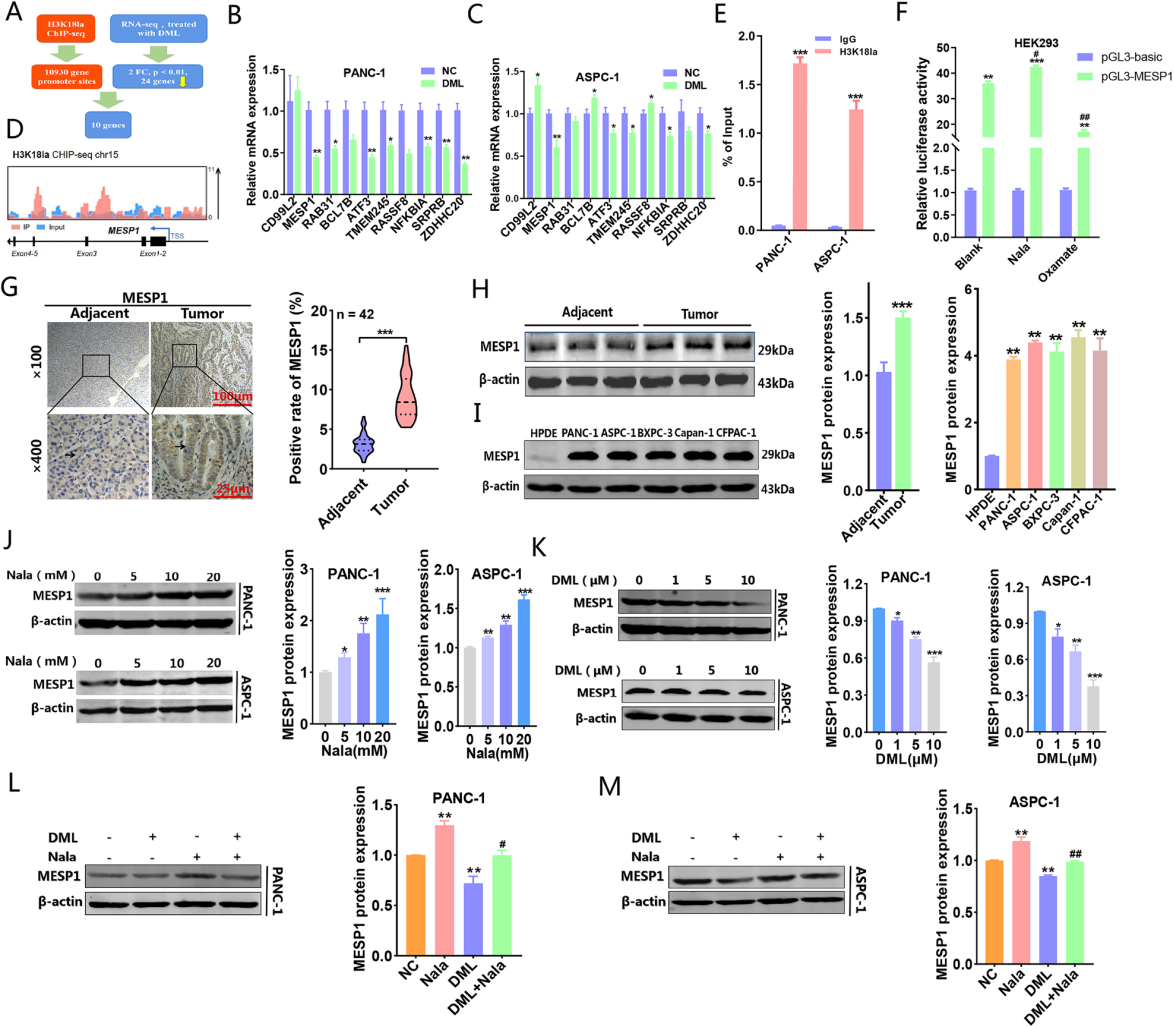
To search for the target protein of DML reducing H3K18la inhibiting PC progression. **A** Mechanistic diagram of the exploring of target proteins for which DML exerts its function by inhibiting H3K18la. The regulatory effects of DML on candidate genes were verified by qRT-PCR in PANC-1 (**B**) and ASPC-1 (**C**). **D** IGV tracks for MESP1 from ChIP-seq analysis. **E** The binding relationship between H3K18la and the MESP1 promoter region was analyzed by ChIP-qPCR using H3K18la or IgG antibodies in the PANC-1 and ASPC-1 cell lines. **F** The pGL3-MESP1 reporter gene vector was constructed, and the dual-luciferase reporter gene assay was used to explore the effect of H3K18la on the promoter activity and transcriptional level of MESP1. **G** MESP1 expression level was detected by IHC in normal and tumor tissues. Western blot was used to detect the expression of MESP1 in PC tissues (**H**) and cell lines (**I**). Detected the effect of different concentrations of DML (**J**) and Nala (**K**) on MESP1 expression levels by western blot. Nala rescued the inhibitory effect of DML on MESP1 expression levels by western blot in PANC-1 (**L**) and ASPC-1 (**M**). All data are shown as the mean ± SD of at least three independent experiments. *p < 0.05, **p < 0.01, ***p < 0.001, vs. NC, IgG or pGL3-basic. ^#^*p* < 0.05, ^##^*p* < 0.01, vs. DML or *p*GL3-MESP1 of Blank.

In order to delve deeper into the mechanism through which DML decreased H3K18la and consequently impeded PC progression, we collected total RNA from PC cells subjected to DML (10 μM) treatment for a duration of 48 h and performed RNA sequencing analysis. In comparison to the control group, we identified 391 upregulated genes and 398 downregulated genes (Supplementary Fig. S2A, B). The KEGG analysis indicated that DML influenced prevalent oncological pathways, encompassing cellular immunity, TNF signaling, NF-kappaB and apoptosis process (Supplementary Fig. S2C). Concurrently, GO analysis of these differentially expressed genes (DEGs) distinctly pointed out that DML predominantly affects tumor-associated functional fields, including tumor necrosis, cellular proliferation, and cell apoptosis (Supplementary Fig. S2D). These findings preliminarily illustrated the substantial potential of DML in regulating cancer progression. Given that the above results suggested DML exerted a tumor-suppressive effect by inhibiting H3K18la, we proceeded to investigate downstream target genes utilizing H3K18la antibodies for chromatin immunoprecipitation and sequencing (ChIP-seq). GO and KEGG analyses revealed that genes specifically binding to H3K18la are primarily involved in cancer cell functional pathways, such as wound-healing, intercellular adhesion, and cell junction, implying a regulatory role of H3K18la in PC (Supplementary Fig. S2E–G). Subsequently, we conducted a cross-analysis of the RNA-seq and ChIP-seq datasets to pinpoint ten candidate target genes that exhibited binding to H3K18la and were modulated by DML (Fig. 4A). PANC-1 and ASPC-1 cell lines were subjected to DML treatment, followed by qRT-PCR validation of these ten genes, where we assessed their expression levels in PC cells versus normal pancreatic ductal epithelial cells. The results indicated that, in comparison to the other candidate genes, the mRNA expression of MESP1 was markedly downregulated by DML (Fig. 4B, C), while it showed elevated expression in cancer cells (Supplementary Fig. S2H). Furthermore, ChIP-seq analyses revealed that H3K18la was significantly enriched in the promoter region of the MESP1, indicating its potential role in the regulation of MESP1 transcription (Fig. 4D). ChIP-qPCR and dual-luciferase reporter assays demonstrated the binding of H3K18la to the promoter region of MESP1. Furthermore, the upregulation of H3K18la levels activated the promoter activity of MESP1, thereby promoting the initiation of transcription. Conversely, the downregulation of H3K18la levels suppressed MESP1 promoter activity, leading to the inhibition of MESP1 transcription (Fig. 4E, F). Immunohistochemical staining and western blot assays demonstrated that MESP1 expression was markedly increased in human PC tissues and cell lines (Fig. 4G–I). Nala enhanced MESP1 expression in a concentration-dependent fashion (Fig. 4J), whereas DML suppressed its expression (Fig. 4K). Moreover, the external supplementation of Nala counteracted the suppression of MESP1 expression caused by DML (Fig. 4L, M). These findings demonstrated that MESP1 expression was markedly elevated in PC tissues and cell lines. DML diminished MESP1 transcription and expression levels through the inhibition of H3K18la.

### The suppression of MESP1 encouraged the apoptosis of PC cells while simultaneously hindering their proliferation, invasion, and migration

**Fig. 5:**
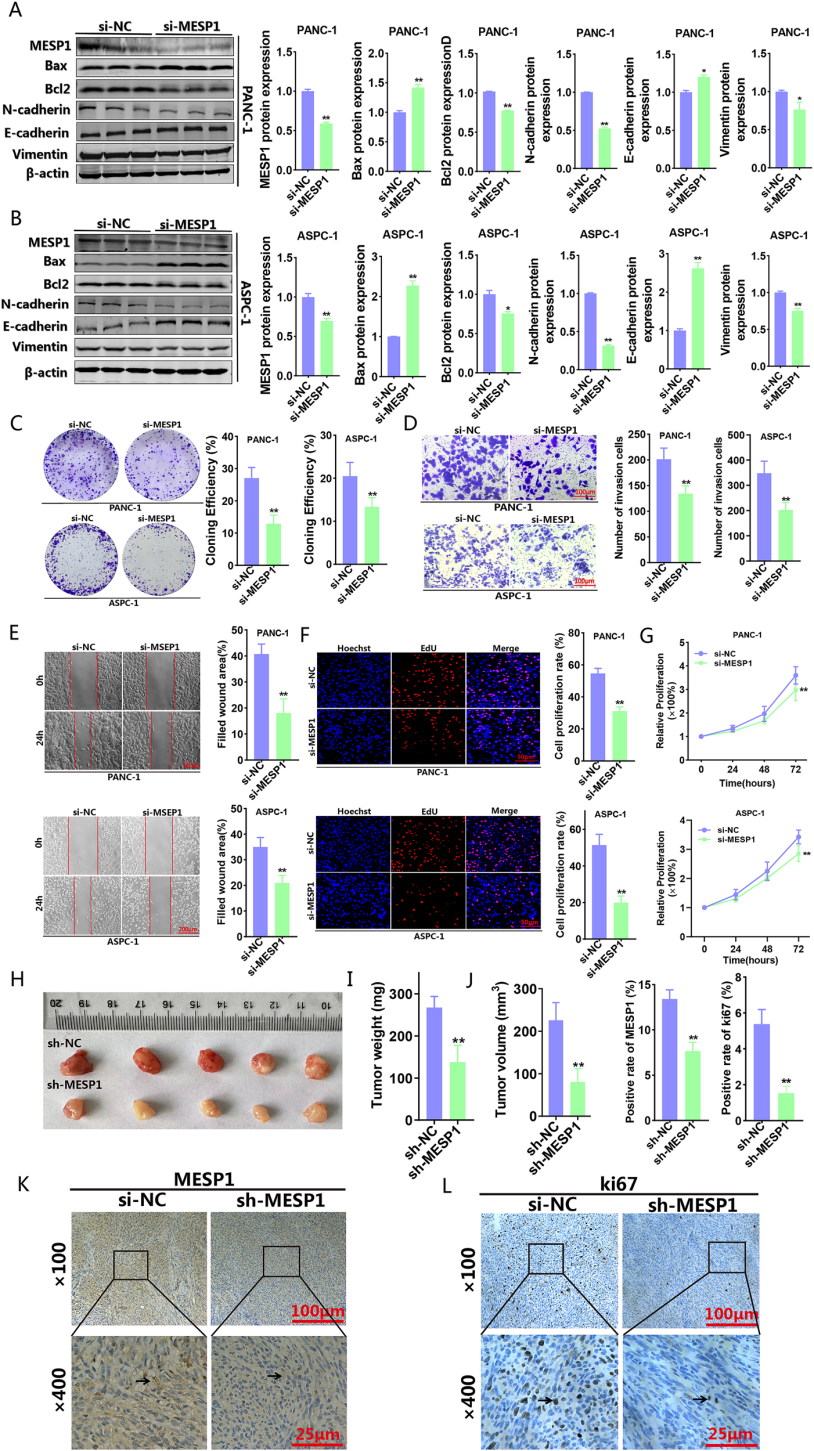
To investigate the function of MESP1 in PC cell lines. Western blot was used to detect the effect of silencing MESP1 on the expression levels of Bax, Bcl2, N-cadherin, E-cadherin and Vimentin in PANC-1 (**A**) and ASPC-1 cells (**B**). Colony formation (**C**) transwell invasion (**D**) wound-healing (**E**) EdU (**F**) and CCK-8 (**G**) assays investigated the effect of silencing MESP1 on the proliferation, migration and invasion of PC cells. sh-MESP1 cell line was used to construct a xenograft model in nude mice (**H**) and the tumor weight (**I**) and volume (**J**) were measured. MESP1 (**K**) and ki67 (**L**) expression level was detected by IHC in sh-NC and sh-MESP1 animal tumor tissues. All data are shown as the mean ± SD of at least three independent experiments. *p < 0.05, **p < 0.01, ***p < 0.001, vs. si-NC.

Due to the limited research available on the function of MESP1 in PC, we constructed small interfering RNAs (siRNAs) aimed at silencing MESP1 expression to investigate its role. Subsequently, western blot analyses revealed that the downregulation of MESP1 expression led to an increase in Bax protein levels, a decrease in Bcl2 protein levels, enhanced cancer cell apoptosis rate, suppression of EMT, and a reduction in metastasis of PC cells (Fig. 5A, B and Supplementary Fig. S1E, F). A series of functional assays demonstrated that silencing MESP1 significantly impaired cell colony formation, proliferation, invasion, and migration capabilities (Fig. 5C–G). Additionally, we constructed a stable MESP1 knockout cell line and investigated the effects of MESP1 in vivo (Fig. 5H). The results indicated that MESP1 knockout inhibited the growth of subcutaneous tumors (Fig. 5I, J). Immunohistochemical evaluations additionally demonstrated that in comparison to the sh-NC group, the sh-MESP1 group displayed a diminished expression of Ki67 and a decrease in tumor proliferation (Fig. 5K, L).

### DML reduced MESP1 expression by inhibiting H3K18la, thereby suppressing the malignant behavior of PC cells

**Fig. 6:**
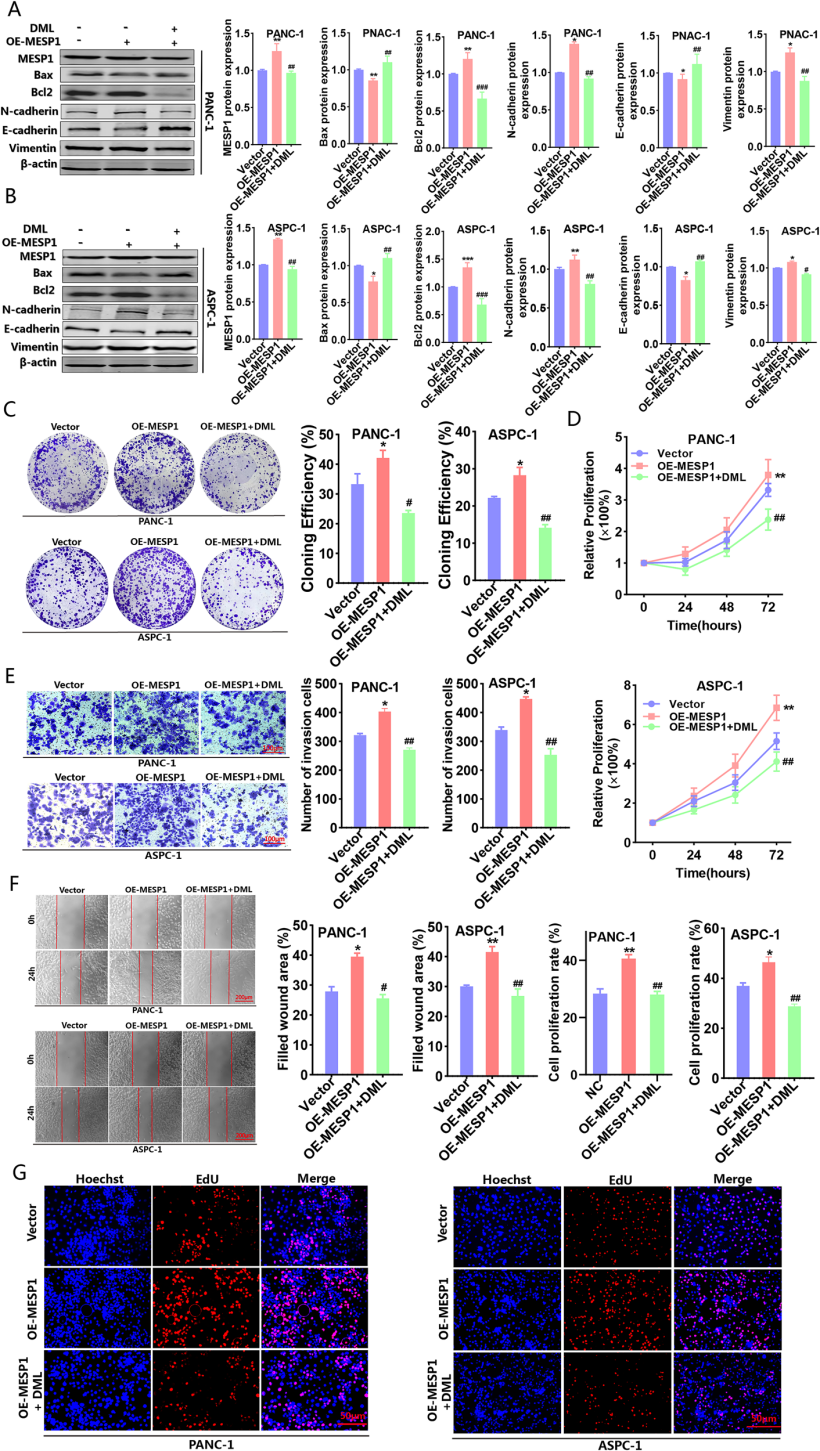
MESP1 was confirmed to be the key protein in DML inhibiting H3K18la to prevent PC progression. Western blot was used to detect the effect of upregulating MESP1 on the expression levels of Bax, Bcl2, N-cadherin, E-cadherin and Vimentin in PANC-1 (**A**) and ASPC-1 cells (**B**) treated by DML. Colony formation (**C**) CCK-8 (**D**) transwell invasion (**E**) wound-healing (**F**), and EdU (**G**) assays investigated the effect of upregulating MESP1 on the proliferation, migration and invasion of PC cells treated with DML. All data are shown as the mean ± SD of at least three independent experiments. *p < 0.05, **p < 0.01, ***p < 0.001, vs. Vector. ^#^*p* < 0.05, ^##^*p* < 0.01, ^###^*p* < 0.001 vs. OE-MESP1.

**Fig. 7:**
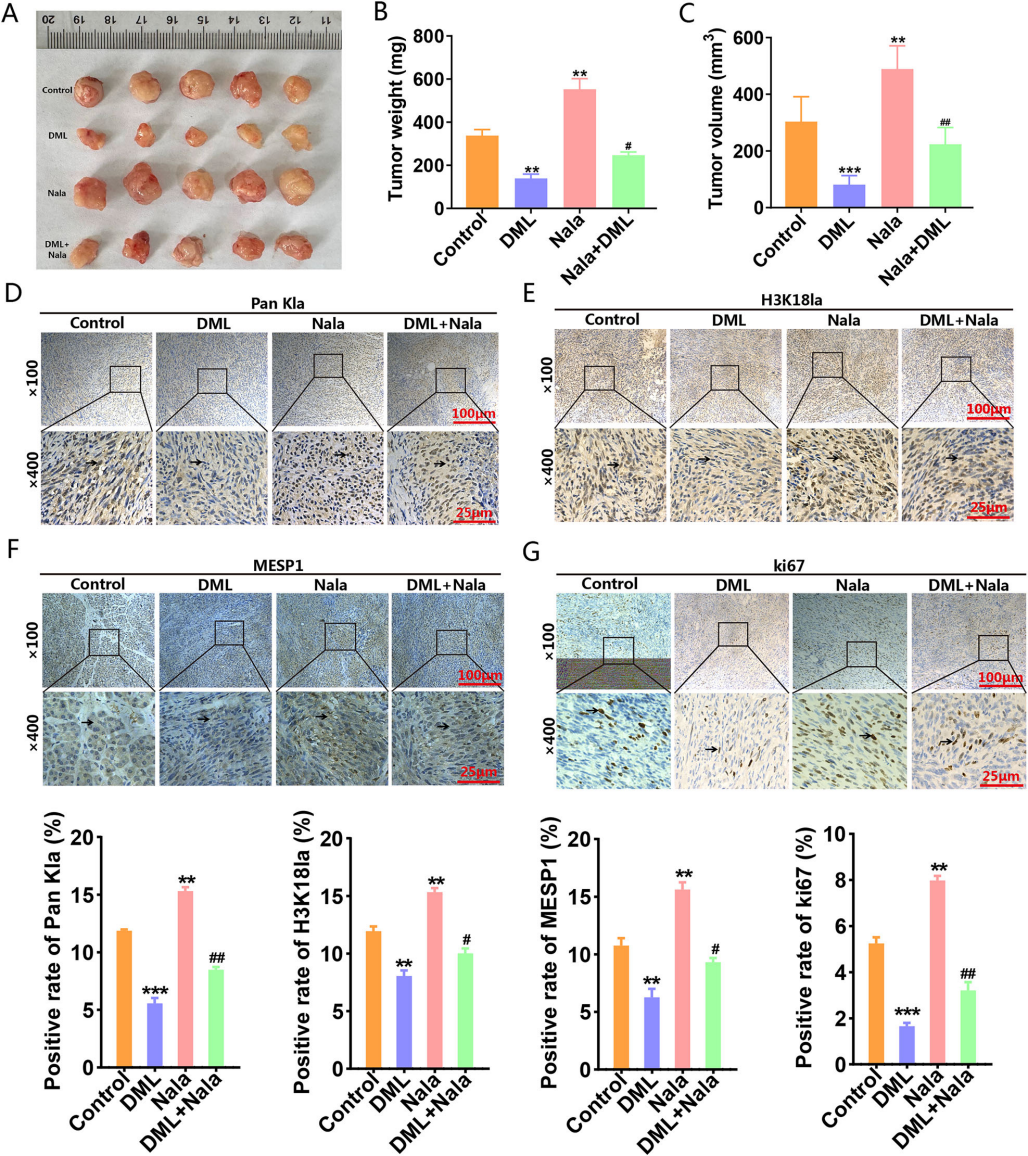
Regulation of DML on PC cell growth and MESP1 expression in vivo. **A** General pictures of animal demonstrated the effects of Nala (20 mM) and DML (30 mg/kg) treated PANC-1 cells in xenografts. 20 days after treatment, the nude mice were sacrificed to detect the tumor weight (**B**) and volume (**C**). **D**–**G** Pan Kla, H3K18la, MESP1 and ki67 expression level were detected by IHC in tumor tissues. All data are shown as the mean ± SD of at least three independent experiments. **p < 0.01, ***p < 0.001, vs. Control. ^#^*p* < 0.05, ^##^*p* < 0.01, vs. DML.

Subsequently, we constructed a vector for the overexpression of MESP1, resulting in a successful enhancement of MESP1 expression in PC cells. We aimed to verify whether DML contributes to the downregulation of MESP1 expression through the inhibition of H3K18la, thereby attenuating the malignant characteristics of PC cells. Western blot analysis demonstrated that MESP1 overexpression decreased Bax levels, increased Bcl2 expression, and consequently, inhibited apoptosis. Moreover, MESP1 overexpression promoted the EMT process and accelerated metastasis in PC cells. Importantly, DML reversed the apoptosis inhibition and metastatic promotion induced by MESP1 overexpression (Fig. 6A, B and Supplementary Fig. S1G, H). Functional experiments verified that DML counteracted the promoting effects of MESP1 overexpression on cell proliferation, invasion and migration (Fig. 6C–G). Subsequently, we constructed subcutaneous xenograft models to explore the function and mechanism of DML in vivo (Fig. 7A). The results indicated that in contrast to the control group, tumors in the Nala group were heavier and larger, whereas tumors in the DML group were lighter and smaller. Compared to the DML group, adding Nala partially mitigated the inhibitory effect of DML on tumor growth (Fig. 7B, C). Immunohistochemistry analysis demonstrated that DML inhibited the expression levels of Pan Kla, H3K18la, MESP1, and Ki67 in vivo, while Nala partially reinstated their expression levels (Fig. 7D–G). These results indicate that DML downregulates the expression of MESP1 by inhibiting H3K18la, thereby suppressing the proliferation, invasion, and migration of PC cells and promoting apoptosis. The downstream mechanisms of MESP1 in PC remain unexplored. To investigate this, we silenced MESP1 in the PANC-1 cell line for RNA-seq and performed a comprehensive analysis of the mechanistic pathways and molecular functions potentially associated with MESP1. The RNA-seq underscore the pivotal role of MESP1 in diverse cellular physiological mechanisms and cancer pathways, establishing it as a vital regulator of pancreatic cancer progression with substantial implications for research and therapeutic strategies (Supplementary Fig. S3A–I). We will conduct a more in-depth exploration of the specific mechanism of MESP1 in future research.

## Discussion

Extensive research has indicated that various forms of histone post-translational modifications are linked to the EMT in PC, facilitating the proliferation, invasion, and migration of PC cells. Lactylation, a relatively novel form of histone post-translational modification reliant on lactic acid, remains underexplored in the context of PC. Given that PC is characterized by a heightened glycolytic demand, there is a significant accumulation of lactic acid within the tumor cells, thereby supplying ample substrate for lactylation processes. Moreover, an analysis utilizing the TCGA database revealed a negative correlation between the expression of enzymes associated with lactic acid production and the survival rates of PC patients. In a cohort of 42 PC patient tissue samples, we observed notably elevated expression levels of Pan Kla and H3K18la in malignant tissues. Furthermore, a reduction in H3K18la levels was found to significantly suppress the malignant behaviors of PC cells while promoting apoptotic pathways. These evidences underscore the critical role of histone lactylation in facilitating the progression of PC.

DML is considerably present in TwHF (1.973 mg/g) and demonstrates low toxicity, rendering it advantageous for advancements in pharmaceutical development. Furthermore, research indicates that DML improves the chemosensitivity of PC cells to gemcitabine and colorectal cancer cells to 5-fluorouracil [12, 21]. Nonetheless, the underlying mechanism by which DML operates in PC remains ambiguous, posing challenges for its drug development and clinical application. Our experimental findings revealed that DML decreased lactate levels and downregulated H3K18la in PC cells and curtailed proliferation, invasion, migration, and clonal formation of PC cells while promoting apoptosis. Co-treatment with Nala partially mitigated the tumor-suppressive effects of DML, indicating that the regulation of histone lactylation is a crucial mechanism through which DML exerts its anticancer activity.

To further investigate the mechanism by which DML downregulation of H3K18la impedes PC progression, we identified potential downstream target genes through cross-analysis of multi-omics sequencing, a vital methodological approach that enables comprehensive and accurate identification of research pathways. we initially conducted RNA sequencing on both DML-treated and untreated groups, followed by ChIP sequencing utilizing an H3K18la antibody. This cross-analysis of the two datasets yielded ten candidate genes that are modulated by DML and demonstrate binding affinity to H3K18la. To rigorously screen for target genes, we employed PCR to validate the sequencing outcomes, revealing that the mRNA level of MESP1 was markedly downregulated by DML, while MESP1 expression was significantly elevated in PC cells. DML’s inhibition of MESP1 protein levels exhibited a concentration-dependent response, which was subsequently reversed by Nala treatment. A thorough analysis confirmed that MESP1 serves as a downstream target of DML’s regulatory effect on H3K18la levels.

The expression and functionality of MESP1 in PC were explored for the first time, and our findings revealed a significant upregulation of MESP1 in both PC tissues and various cell lines. The silencing of MESP1 resulted in the repression of malignant biological characteristics in PC cells, facilitated apoptosis, restricted the EMT, and decelerated the progression of PC. MESP1 is a pivotal protein through which DML exerts its anti-cancer effects by regulating H3K18la. As a transcription factor, MESP1 possesses the ability to bind directly to DNA, thereby modifying the expression of numerous genes that are integral to cell adhesion, migration, and other cancer-associated traits, thus contributing to tumorigenesis and development. Prior investigations have established that elevated MESP1 expression inhibits apoptosis and promotes cell cycle advancement [24]. The suppression of apoptosis is facilitated through pathways such as caspase-3/PARP1, which are essential for programmed cell death [22]. RNA-seq of silencing MESP1 indicated that MESP1 was linked to multiple cancer-associated pathways, including TNF, JAK-STAT, Wnt, and Hippo pathways. Consequently, the enriched pathways were categorized and analyzed, demonstrating that MESP1 predominantly influences cytokine signaling pathways, apoptosis, cellular senescence, the cell cycle, and cell adhesion in cellular physiological processes. PC is noted for its high metabolic rate. Beyond energy, the rapid proliferation of PC cells necessitates a significant supply of biological macromolecules (nucleic acids, lipids, and proteins) as substrates for synthesis. Hence, the levels and metabolism of carbon molecules within cells are particularly pivotal. Bioinformatics analysis revealed a correlation between MESP1 and metabolic pathways such as MAPK and PI3K-AKT, some of which demonstrated enrichment in carbon metabolism. These results underscore the critical role of MESP1 in the progression of PC, positioning it as a potential target molecule for the diagnosis, treatment, or prognosis of this malignancy.

At present, the establishment of genetic-level diagnostic and therapeutic strategies for PC remains elusive, while epigenetics emerges as a promising avenue for the identification of novel therapeutic targets. Consequently, in this investigation, we have elucidated for the first time a mechanism through which DML impedes the progression of PC by down-regulating H3K18la, subsequently diminishing the expression of the downstream oncogene MESP1. These findings clarify the inhibitory impact and underlying mechanism of DML on PC, thereby contributing to the body of knowledge regarding histone lactylation in the context of PC. This research offers crucial theoretical groundwork for the potential application of DML as a clinical therapeutic agent for PC and pioneers the exploration of the expression, function, and mechanism of MESP1, addressing a significant lacuna in PC research.

Despite the advances made, several challenges remain within this investigation. Lactylation, as a relatively recent modification, lacks comprehensive mechanistic elucidation and the specific enzymes involved remain inadequately characterized, leading to imprecise regulation of lactylation both in vivo and in vitro. Furthermore, RNA-seq and a variety of experimental outcomes indicate that Nala does not fully negate the anticancer properties of DML, suggesting that DML’s influence is not limited to the inhibition of H3K18la but possibly involves additional molecular pathways. Therefore, further investigation into these alternative pathways is vital for advancing DML as a viable therapeutic agent while mitigating potential adverse effects. Lastly, we undertook a thorough analysis of the pathways in which MESP1 may be involved in PC. While we established MESP1’s significance in PC development, our study has not yet provided a precise exploration and validation of these pathways. MESP1 belongs to the bHLHC family and possesses a PAS (PER-ARNT-SIM) domain following the bHLH motif, resulting in a three-dimensional structure characterized by an internal cavity suitable for small-molecule binding and external surfaces conducive to protein-protein interactions [26]. These proteins play pivotal roles in various pro-tumor and antitumor pathways, and their functions can be modulated by both natural and tumor-derived metabolites. Consequently, MESP1 is likely positioned as a target molecule for specific natural metabolites, influencing downstream proteins and cancer progression. It is evident that MESP1 may interact with DNA to regulate oncogene transcription within the nucleus, and may directly associate with cancer-related proteins to modulate their functions in the cytoplasm. Its mechanisms are intricate, and its roles are multifaceted, underscoring its significance for research. We are committed to further elucidating the specific mechanisms of MESP1 in PC with the aim of promoting its early adoption as a target gene for the diagnosis and treatment of this malignancy.

## Materials and methods

### Clinical tissue samples

Twenty-five pairs of human fresh PC and para-cancer tissue samples and 42 pairs of paraffin-embedded PC and para-cancer tissue samples were from the Department of Pathology, Affiliated Cancer Hospital of Harbin Medical University. None of the patients received any radiotherapy or chemotherapy before surgery. The patient’s tissue was frozen in liquid nitrogen immediately after surgical resection for further analysis. All patients signed informed consent before operation. This study was approved by the Ethics Committee of Harbin Medical University. (IRB3002720). Researchers were blinded to the group allocation both during the experiment and/or when assessing the outcome.

### Cell culture and transfection

PANC-1 and BXPC-3 were cultured in DMEM medium, ASPC-1 and HPDE in RPMI1640 medium, and Capan-1 and CFPAC in IMDM medium. All cells were cultured with 10% FBS except Capan-1, which used 20% FBS. All cell cultures were supplemented with 1% antibiotics to prevent contamination, and all cells were cultured in 37 °C incubators containing 5% CO_2_. All cell lines in the study were provided by BeNa Culture Collection (Henan, China). All the siRNAs were synthesized by Guangzhou Ribo Company (Guangzhou, China) and OE-MESP1 plasmid was biosynthesized by Yuanjing Biology (Guangzhou, China). Both siRNAs and OE-MESP1 plasmids were diluted with opti and transfected with lipo-2000. Thirty to fifty percent cells were inoculated in six-well plates, and 50 μM siRNAs/2000 ng OE-MESP1 plasmid and 8 μl lipo2000 were added to each well. 12-well plates, 24-well plates and 96-well plates were halved successively. (si-LDHA: CTGGAAGATAGGTTT; si-NC: UUUGUACUACACAAAAGUACUG; si-MESP1: GCCTCAGCGAGGAGAGTCT)

### Construction of stable cell line

Yuanjing Biological Corporation (Guangzhou, China) designed and synthesized the sh-MESP1 lentivirus for our study. Thirty to fifty percent of the cells were uniformly inoculated in the culture plate, cells from 2–3 wells were digested into single-cell suspension and cell counts were performed to calculate the average number of cells per well. The original medium was discarded, 1/2 volume of fresh medium was added, and polybrene was added until the final concentration was 8 μg/mL. According to the titer of the virus (10^8^ TU/mL), the virus were directly added into the cells, shaken evenly, and then placed in the incubator for further culture. The formula for calculating virus dosage is V (μl) = 1000 × MOI × N/T. The lentivirus carried puromycin resistance gene, and the puromycin was added to the medium 48 to 72 h after virus infection, and the stable cells were screened and enriched. The culture medium with FBS containing antibiotics was changed every 2–3 days until the control cells completely floated to death.

### ChIP-seq

Ten ng of DNA samples were prepared for illumina sequencing as the following steps: (1) DNA samples were blunt-ended; (2) A dA base was added to the 3’ end of each strand; (3) illumina’s genomic adapters were ligated to the DNA fragments; (4) PCR amplification was performed to enrich ligated fragments; (5) Size selection of 200–1500 bp enriched product using AMPure XP beads. The completed libraries were quantified by Agilent 2100 Bioanalyzer. The libraries were denatured with 0.1 M NaOH to generate single-stranded DNA molecules, captured on illumina flow cell, amplified in situ. The libraries were then sequenced on the Illumina NovaSeq 6000 following the NovaSeq 6000 S4 Reagent Kit (300 cycles) protocol.

### RNA-seq

The experimental procedure of digital RNA sequencing: after the extracted total RNA samples were inspected and quantified by agarose electrophoresis and Nanodrop, the mRNA was enriched with oligo (dT) magnetic beads (if the RNA was degraded or prokaryotic, the RNA removal kit was directly used): the RNA sequencing library was completed by the kit. The first strand cDNA was generated by random primers after RNA fragmentation, the second strand cDNA was synthesized by adding dUTP, the double-strand cDNA was repaired at the end and connected with Illumina matching splitter after A, and the final library was amplified by PCR. The constructed library was inspected by Agilent2100, quantified by qPCR, and sequenced by Illumina Nova Seq 6000 sequencer.

### Xenograft tumor models

The 4–6-week-old nude mice (Charles River, Beijing, China) were randomly assigned to the experimental group and control groups. One week before modeling, nude mice were intraperitoneally injected with sodium lactate at a dose of 1 g/kg per day for 7 days. A total of 5 × 10^7^ PC cells were digested and suspended in 200 μl Matrigel (Corning, New York, USA), and injected into the right underarm of each nude mouse. Two weeks later, we administered DML (30 mg/kg, injected intraperitoneally, once every 2 days) to nude mice for 20 days. And then, the nude mice were sacrificed, the tumor weight and volume were measured, the tumor tissues were collected and fixed with paraformaldehyde, and then immunohistochemical analysis was performed. Researchers were blinded to the group allocation both during the experiment and/or when assessing the outcome.

### Cell invasion assay

Transwell (Corning, New York, USA) with a pore size of 8 μm was pre-cooled in the refrigerator, and Matrigel (Corning, New York, USA) diluted by eight times of 100 μl was added to the upper chamber of transwell and placed in the incubator at 37 °C for 30 min, waiting for gel formation. After the matrix gel was solidified, 2 × 10^3^ transfected cells were resuspended in the upper chamber of transwell using serum-free medium. A total of 500 μl medium with 10% FBS was added into the lower chamber of transwell. After incubating in the incubator for 36 h, the cells passing through the lower chamber surface were fixed with cold methanol and stained with crystal violet for 15 min. Then the transwell was washed with PBS, and the bottom membrane of the chamber was removed after drying. The chambers were sealed with neutral gum, the number of invaded cells was observed under a microscope using Image J.

### Wound-healing

PC cells were inoculated in a 6-well plate and transfected when the cells were evenly spread. The tip of a 10 μl pipette was used to make a scratch in the center of the pore plate, and the dropped cells were cleaned with PBS and then cultured in a serum-free medium. Scratch areas at 0 and 24 h were recorded at a fixed point under the microscope. After the photo is taken, the data is processed with the Image J software.

### Cell count kit 8 (CCK-8) assay

About 2500–3000 cells were placed in 96-well plates and transfected when the cell density reached 30–50%. After transfection, 20 μl of CCK-8 detection solution (Meilunbio, Dalian, China) was added to each well at 0, 24, 48 and 72 h, respectively, and the cell was left for 2 h at 37 °C. The cell proliferation rate was observed by the absorbance detection at 450 nm wavelength with an enzyme labeled instrument.

### 5-Ethynyl-2’-deoxyuridine (EdU) assay

A total of 2 × 10^5^ cells were spread on a crawling sheet in a 24-well plate and EdU labeling was performed 48 h after transfection. The prepared EdU medium of 300 μl was added, the liquid was discarded after 2 h, and each cell well was washed with 300 μl PBS twice for 5 min each time. Add 150 μl 4% paraformaldehyde to each well and fix at room temperature for 30 min, then add glycine with 2 mg/mL concentration to neutralize paraformaldehyde. After penetrating the cells with 0.5% TritonX-100, 300 μl 1 × Apollo staining reaction solution was added to each well, and incubated on the decolorizing shaker at room temperature for 30 min away from light. Each well was added with 300 μl 1× Hoechst33342 reaction solution and incubated in a shaker at room temperature for 30 min for DNA staining. The number of proliferating cells was observed with Zeiss Axio Scope A1 microscope and the data was processed with Image J software. The EdU kit in this experiment was provided by Ribo (Guangzhou, China).

### Colony formation experiment

About 1000 cells were inoculated in a 6-well plate and the medium was changed every 3 days. After 14 days, the culture medium was abandoned, the cells were cleaned with PBS, 4% paraformaldehyde was added and fixed at room temperature for 30 min, and the cells were stained with crystal violet solution. After taking photos, the data were processed by Image J software to count the number of colonies.

### Western blot

Protein was extracted from cells and tissues with RAPI lysate (Beyotime, Shanghai, China), protein concentration was detected with BCA (Beyotime, Shanghai, China), protein was treated by 6× loading, and the sample was boiled at 100 °C for 7 min. First of all, 30–40 min of 70 V constant voltage electrophoresis, after the marker has been obviously dispersed, adjust the voltage to 110 V and continue the experiment until all the detected samples are separated. The constant current was set at 300 mA, and the transfer time was set according to the molecular weight of the sample protein. The nitrocellulose filter membrane (Pall Corporation, New York, USA), was placed in 5% skim milk and kept closed for 2 h at room temperature. Overnight incubation of primary antibodies: (Pan Kla, H3K18la, H3 (Jingjie biological, Hangzhou, China), Bcl2, Bax, Vimentin, E-cadherin (Proteintech, Wuhan, China), N-cadherin, MESP1 (Affinity Biosciences, Beijing, China), LDHA (ZenBioScience Chengdu, China))

### RNA extraction, reverse transcription, and quantitative realtime PCR analysis (qRT-PCR)

Total RNA was extracted with Trizol reagent, then cDNA was obtained using reverse transcription kit (Toyobo, Japan). Gene mRNA levels in cells and tissues were detected by qRT-PCR using SYBRGreen Mix kit. The gene sequences in the study are as follows (Table 1):

**Table 1:** All the gene primer sequences used in the qRT-PCR of this study (3’-5’).

Gene	F	R
ZDHHC20	CTCAGTGGCTGGAGAATGGA	GGTTAGTTATGAACAAGTGGTACTC
CD99L2	GGTAAAGGTGATGGCCGGTA	TGAGACCCTGCTGAATGCTG
RASSF8	ACAAGCCACCAAACGCTTAC	TCTGCTGGATGAACTGCTGG
BCL7B	AAGAAGTGGGTGACTGTGGG	AAGCCATTAGGTTCTCGGGC
RAB31	TCCTCATCTGGGACACTGCT	TCGCACTTGTTTCCAGCGAT
TMEM245	TGGCTGACACAAGGGTTAGG	ACCGATGATTGCTCCTTCCA
SRPRB	GTGGTCACTTGGGCTCTAGG	AGGGTCAGGGTCTCACAACT
MESP1	TCGAAGTGGTTCCTTGGCAG	CCTCCTGCTTGCCTCAAAGT
NFKBIA	ATTGCTGAGGCACTTCTGGG	CTCACAGGCAAGGTGTAGGG
ATF3	GATGCTTCAACACCCAGGC	TGACTGATTCCAGCGCAGAG

### Immunohistochemistry (IHC)

The tissue slices with a thickness of 4 μm were baked in the oven for 1 h, then immediately soaked in xylene for 10 min and repeated three times. The slices were hydrated in graded concentrations of ethanol. The slices were immersed in 3% hydrogen peroxide for 10 min. Prepare 2000 ml sodium citrate solution at a ratio of 50:1. Heat the solution in a pressure cooker until the solution in the pot boils. Then heat slice in the solution with the lid closed. After 2 min and 35 s, spray the outer surface of the pot with cold water to cool it down. After removing the lid, place the pressure cooker in a sink filled with cold water and let it cool for 20 min. After drying the film, add 50–60 µl of primary antibody (1:200) and leave at 4 °C overnight. The next day, take out the water at room temperature and let it sit peacefully for 30 min, then add secondary antibody (goat anti-rabbit) and let it sit at room temperature for 30 min. The color development of DAB working liquid lasted for 30 s, during which the color reaction was observed with a microscope, and the color development was terminated by adding distilled water about 30 s. Stained with hematoxylin for 35 s, the sections were dehydrated sequentially in ethanol with a gradient of concentration, and then soaked in 3 parts of xylene for 10 min each time. The positive cells were observed under the microscope after the neutral gum was covered with a glass plate. We processed the IHC images using ImageJ. The positive staining area was extracted using an automated thresholding method. We then calculated the positive area, total area, and the positive rate (positive ratio = positive area / total tissue area × 100%), followed by statistical analysis.

### Lactic acid assay

A total of 5 × 10^6^ cells were homogenized by adding 200 μl normal saline (0.9% NaCl solution). After homogenization, centrifuge at 4 °C, 10,000 × *g* for 10 min, take the supernatant and put it on the ice to be measured. The remaining supernatant was used for protein concentration determination. This is followed according the L-Lactic Acid (LA) Colorimetric Assay Kit instructions from Elabscience (Wuhan China). A standard curve should be made with a standard product before measurement. OD values of each hole were measured at 530 nm by enzyme-labeled instrument. The formula for calculating lactic acid content in cells is: LA (mmol/gprot) = (δA530-b) ÷ a ÷ Cpr × f (ΔA530: OD value for sample determination − OD value for blank) (OD value when the standard concentration is 0). f: dilution times of sample before adding to the detection system Cpr: protein concentration of sample to be tested (gprot/L)) (Table 2).

**Table 2:** All the abbreviations that appear in this article.

bHLH	Helix-loop-helix
BMDM	Bone marrow macrophage
ChIP-seq	Chromatin immunoprecipitation and sequencing
CMS	Cardiomyocytes
DML	Demethylzeylasteral
ECs	Endothelial cells
GCN5	General control of nucleotide synthesis
Hippo	Hippopotamus
HDAC	Histone deacetylase
iPSCs	Induced pluripotent stem cells
JAK-STAT	Janus kinase signal transducer and activator of transcription
LSD1	Histone lysine-specific demethylase 1
MESP1	Mesoderm-related factor 1
MAPK	Mitogen-activated protein kinase
Nala	Sodium lactate
NSCLC	Non-small cell lung cancer
PC	Pancreatic cancer
PI3K-AKT	Phosphoinositide 3-kinase-Protein Kinase B
PKM	Pulemyot Kalashnikova
PTEN	Gene of phosphate and tension homology deleted on chromosome ten
PAS	PER-ARNT-SIM
SIRT	Sirtuin 1
SLC16A1	Recombinant Solute Carrier Family 16, Member 1
SMCs	Smooth muscle cells
TCGA	The cancer genome atlas
TwHF	Tripterygium wilfordii Hook F
TNF	Tumor necrosis factor

### Dual-luciferase reporter assay

The promoter of MESP1 was cloned into the firefly luciferase vector to construct the pGL3-MESP1, and the empty vector was pGL3-basic. After treating the cells with Nala/oxamate for 24 h, the 0.5 ug/ml plasmid vector was transfected with lipofectamine™ 2000 transfection reagent. After transfection for 36 h, samples were collected for dual-luciferase detection. The Luciferase activity of the samples was detected using the dual-luciferase reporter assay system (E1910) of Promega company.

### Flow cytometry

A total of 10^5^ cells were collected to prepare a single-cell suspension and rinsed with PBS. The cells were fixed at 4 °C for more than 2 h with 70% ethanol. The cell precipitates were collected by centrifugation. Then, pre-cooled PBS was added to resuspend the cells and the cell precipitates were collected. Cell staining was performed using the Cell Cycle and Apoptosis Analysis Kit from Biosharp company. Red fluorescence was detected at the excitation wavelength of 488 nm by flow cytometry, and the light scattering was also detected simultaneously. The apoptosis rate of cells was analyzed and obtained.

### Statistical analysis

The statistical analyses were mainly conducted using SPSS 16.0 (IBM, SPSS, Chicago, IL, USA) and GraphPad Prism 8.0 (GraphPad Software Inc., CA, USA). All data are presented as means ± standard deviation (SD). Researchers were blinded to the group allocation both during the experiment and/or when assessing the outcome. All experiments were repeated at least three times independently, and two-tailed unpaired *t*-test was applied for two groups comparison analysis. *p* < 0.05 was considered statistically significant.

## Supplementary information

Original western blots

Supplementary results

## Data Availability

Two RNA-seq and one ChIP-seq data have been uploaded to the NCBI SRA database (https://www.ncbi.nlm.nih.gov/sra/PRJNA1160192). The data generated or analyzed in the current study are available from the corresponding author upon reasonable request.
