# Supplementary material for: Demethylzeylasteral suppresses the expression of MESP1 by reducing H3K18la level to inhibit the malignant behaviors of pancreatic cancer
Source: Cell Death Discov. 2025 Jul 3;11:305. doi: 10.1038/s41420-025-02603-9 (PMC12229608; doi:10.1038/s41420-025-02603-9)

Figure1E:

Pan Kla ，n = 6

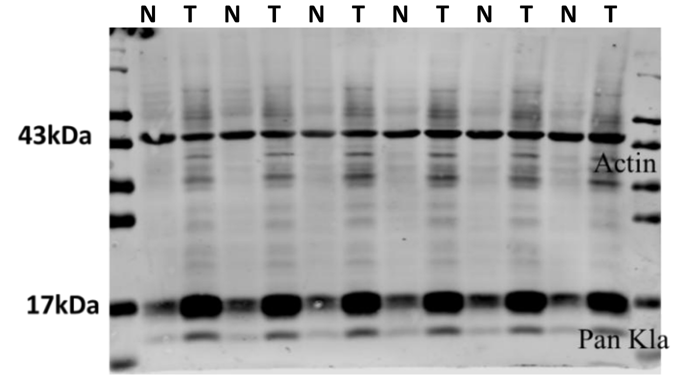

H3K18la，n=6

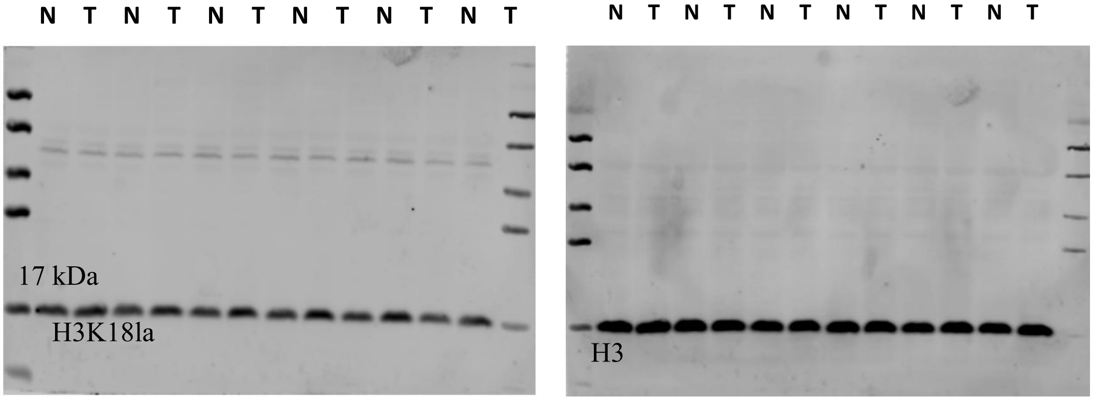

H3K9la，n=6

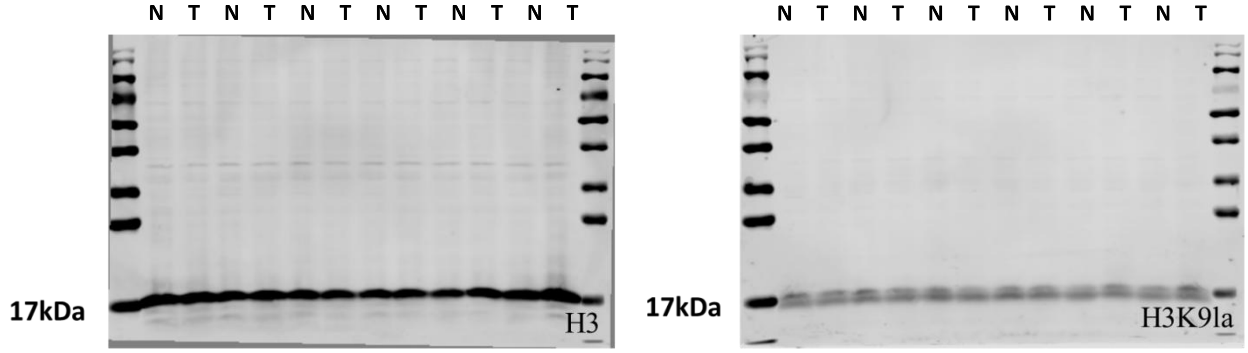

H3K14la，n=6

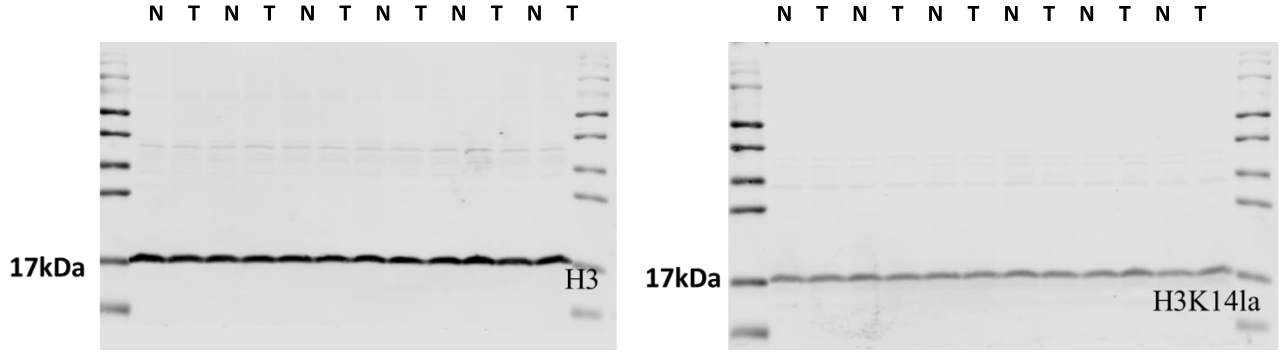

H3K27la，n=6

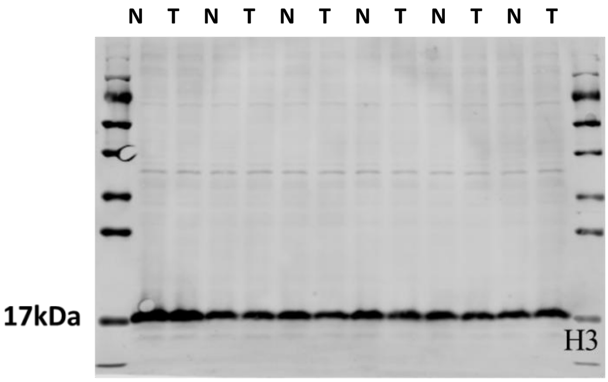

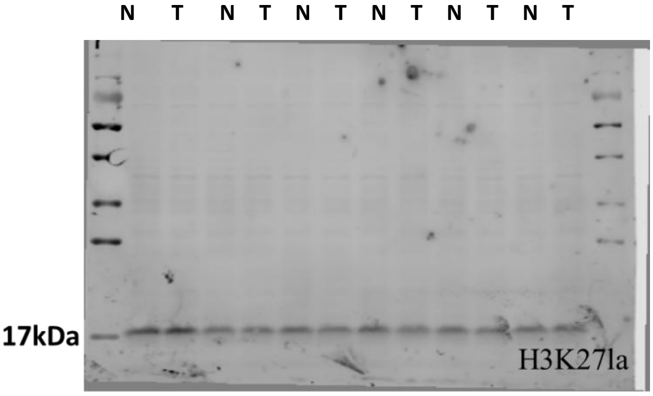

H3K56la，n=6

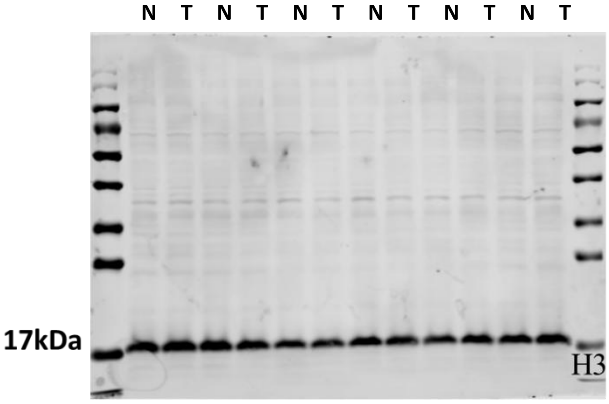

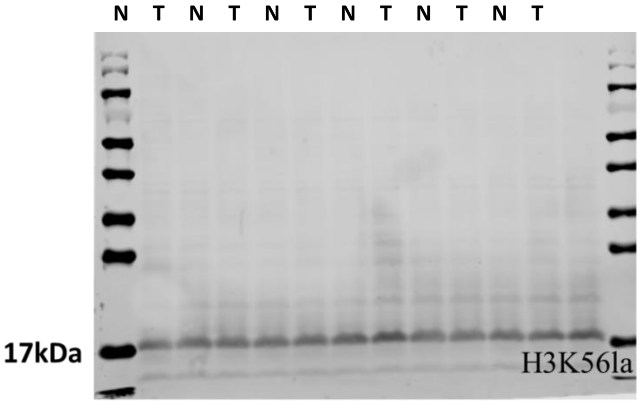

Figure1F:

Pan Kla，n = 5

1、 2、

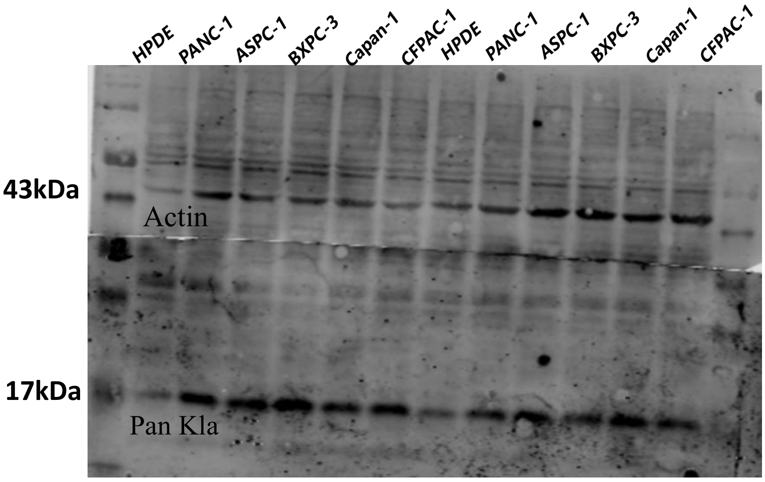

3、

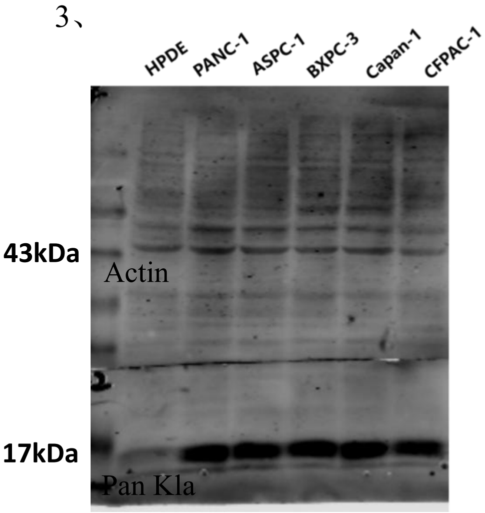

4、

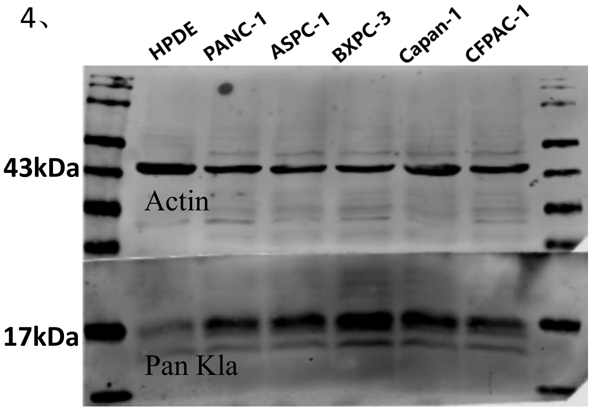

5、

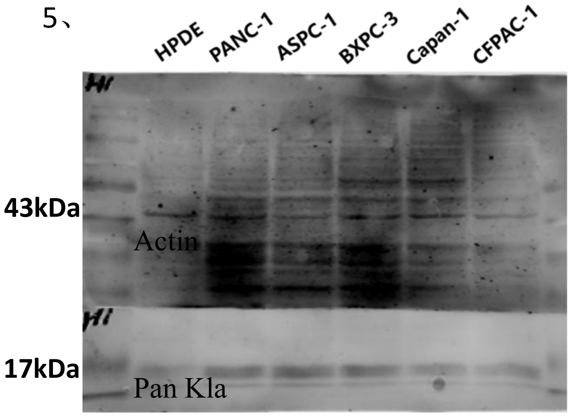

H3K18la，n=3

1、

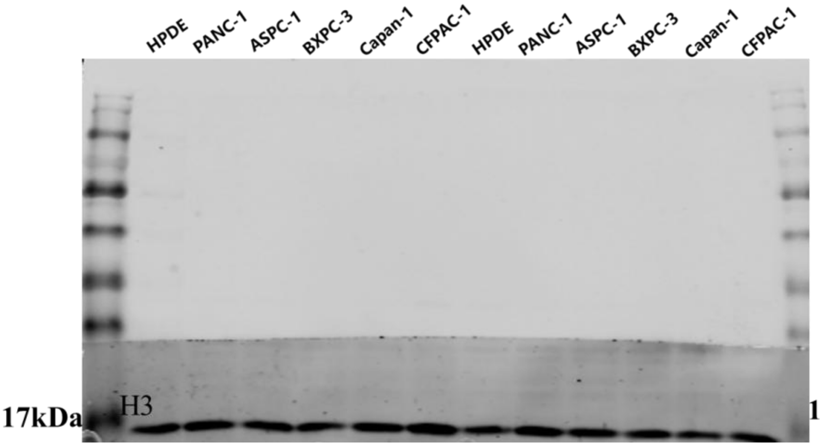

2、

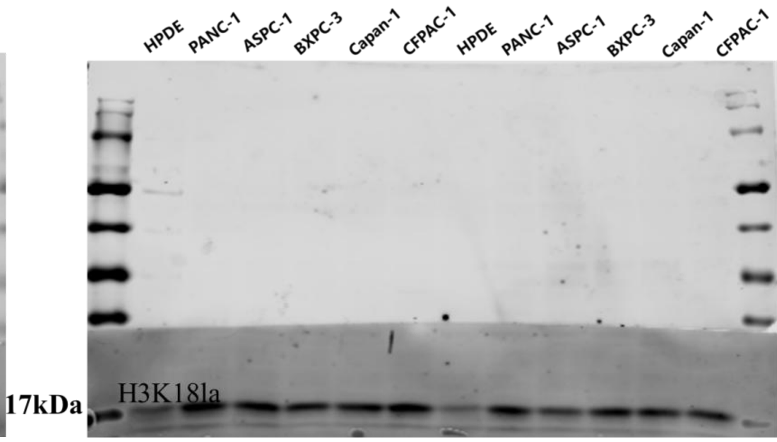

3、

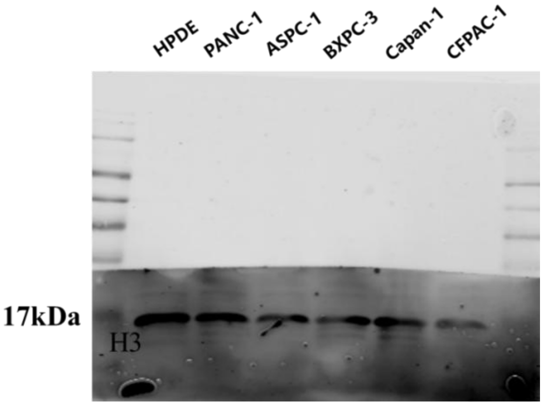

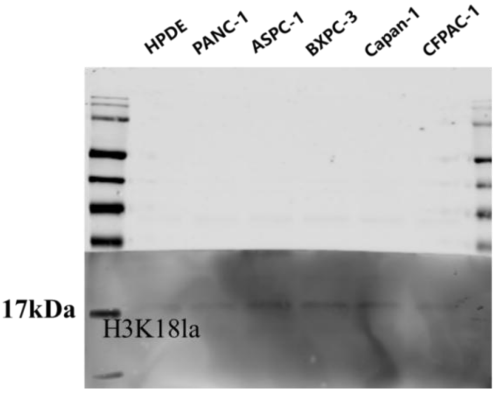

Figure1L:

Pan Kla，n = 3

1、

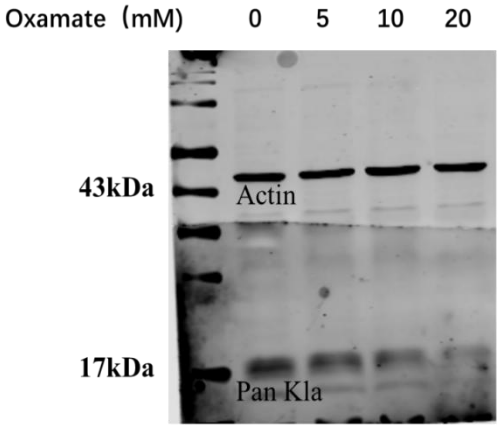

2、

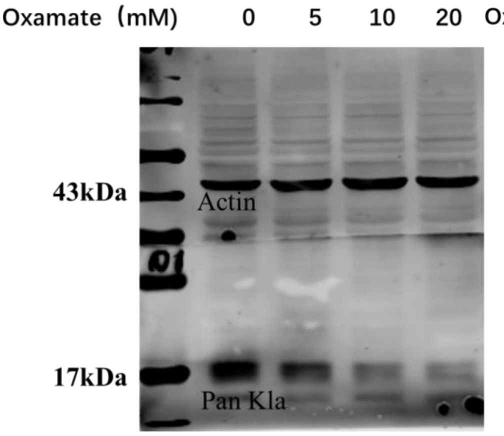

3、

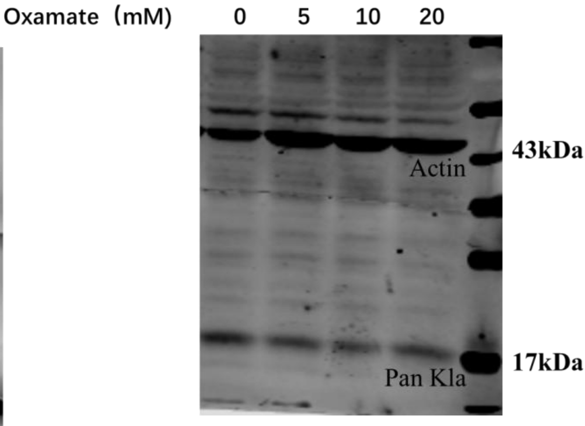

H3K18la，n=3

1、

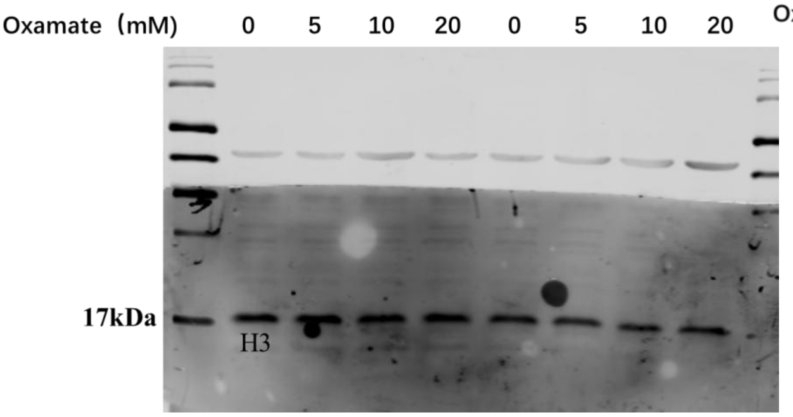

2、

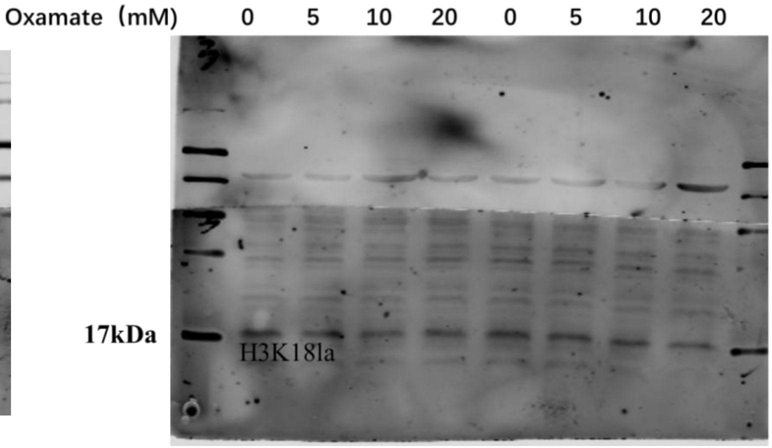

3、

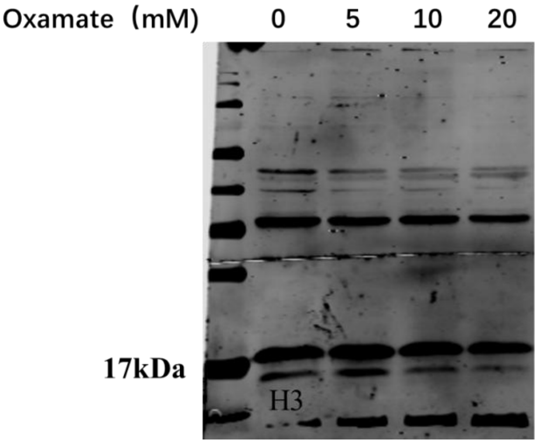

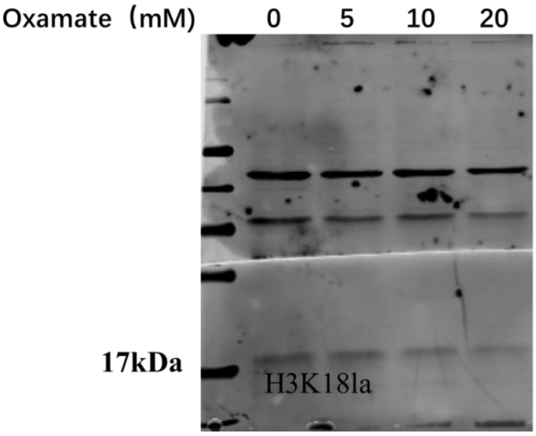

Figure1N:

Pan Kla，n = 3

1、

Nala（mM） 0 5 10 20

43kDa

17kDa

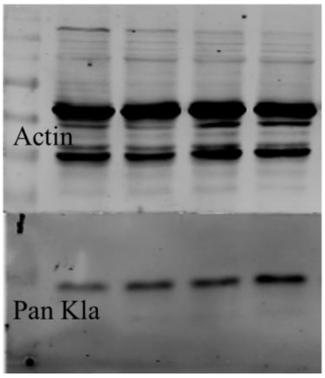

2、

Nala（mM） 0 5 10 20

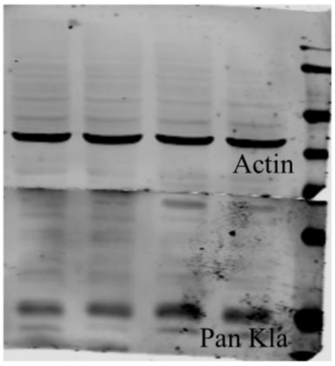

17kDa

3、

Nala（mM） 0 5 10 20

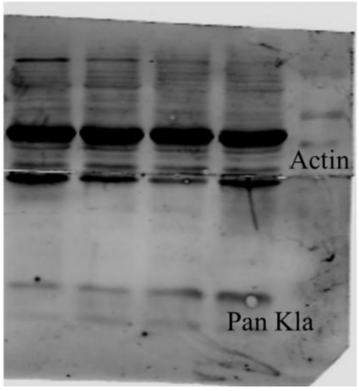

43kDa

17kDa

H3K18la，n=3

1、

Nala（mM） 0 5 10 20

17kDa

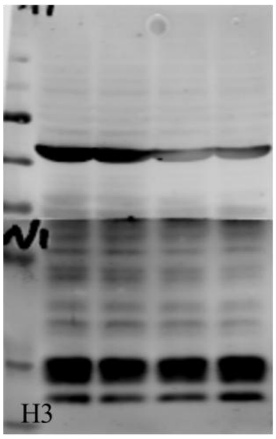

Nala（mM） 0 5 10 20

17kDa

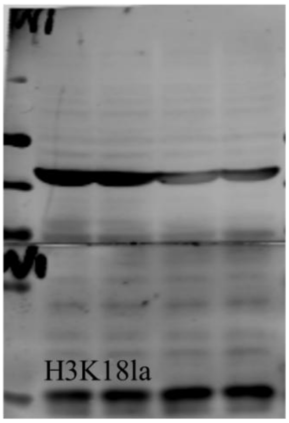

2、

Nala（mM） 0 5 10 20

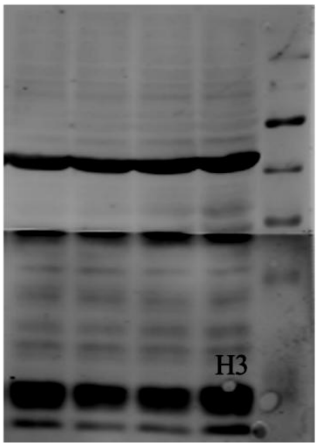

17kDa

Nala（mM） 0 5 10 20

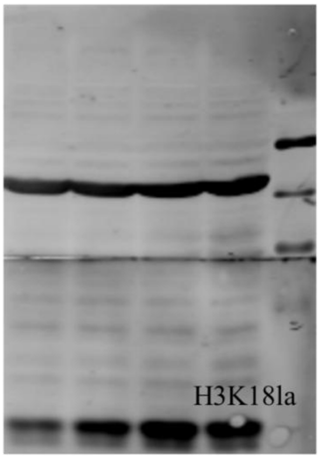

17kDa

3、Nala（mM） 0 5 10 20

17kDa

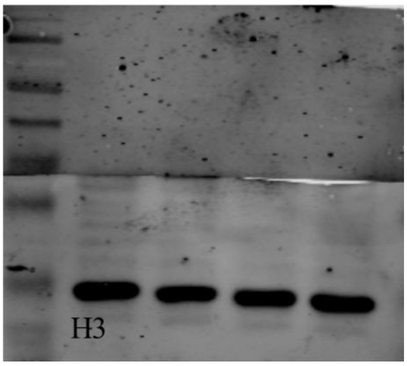

Nala（mM） 0 5 10 20

17kDa

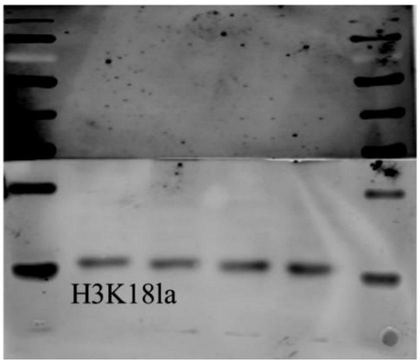

Figure1M:

Pan Kla，n = 3

1、

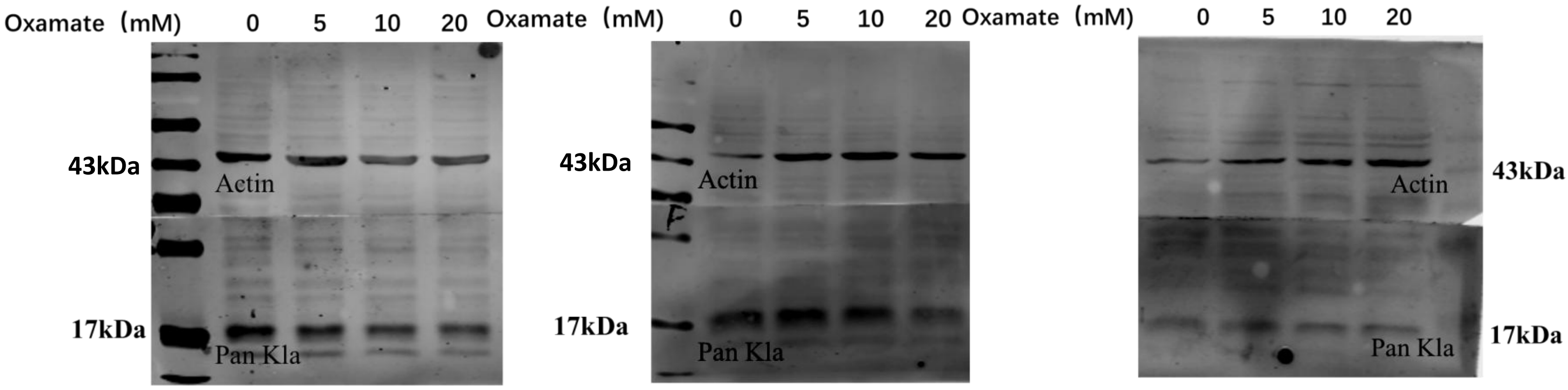

H3K18la，n=4

1、 2、

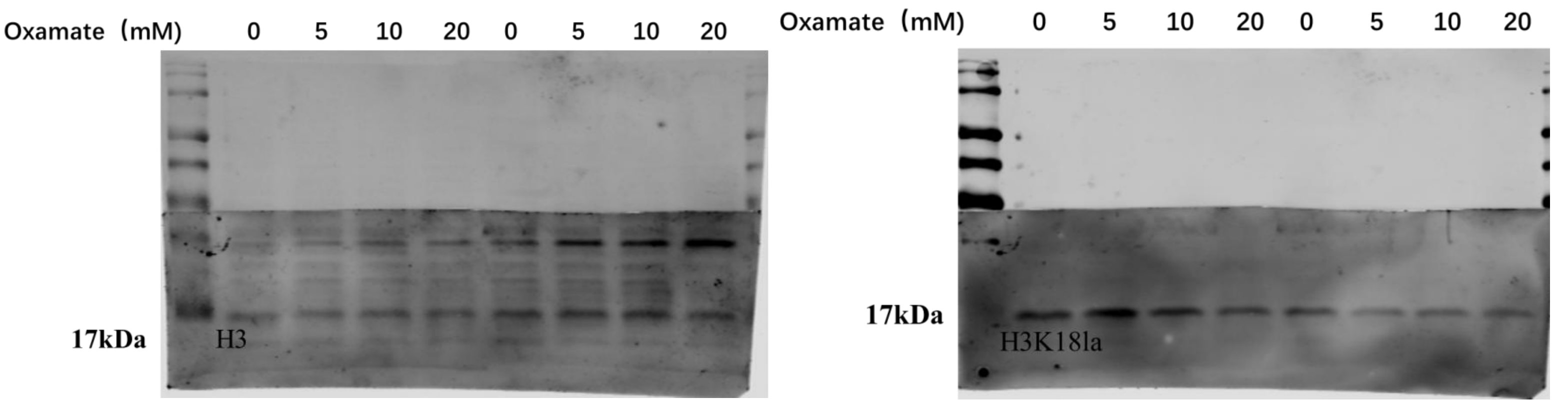

3、

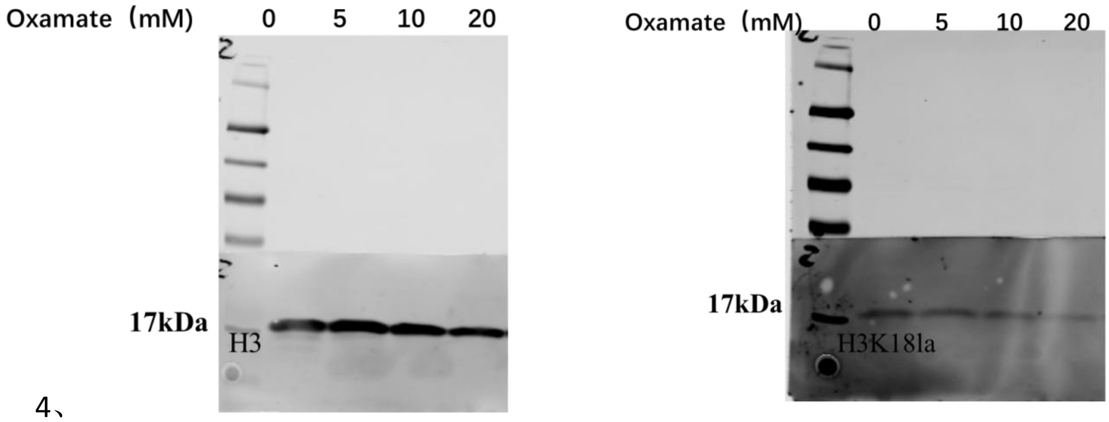

4、

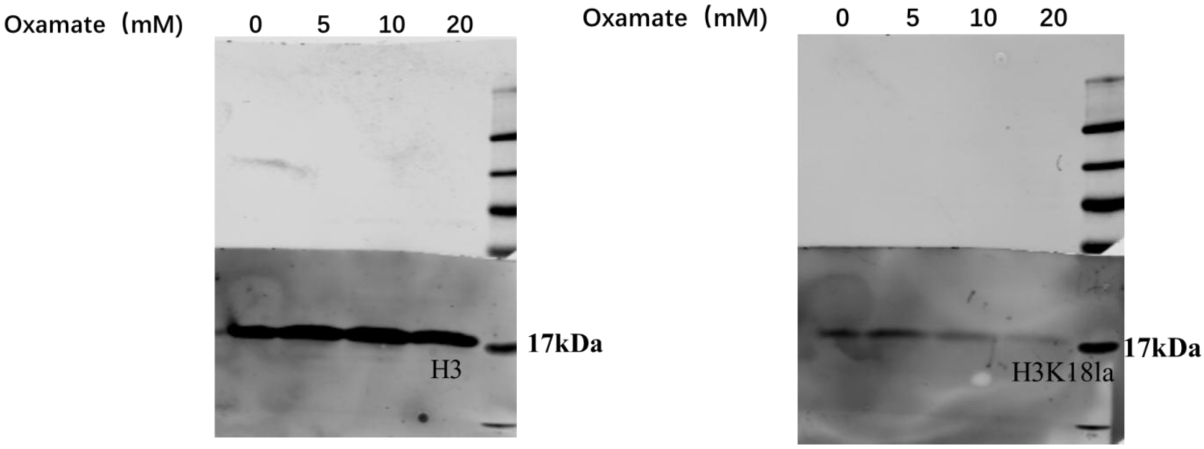

Figure1O:

Pan Kla，n = 3

1、

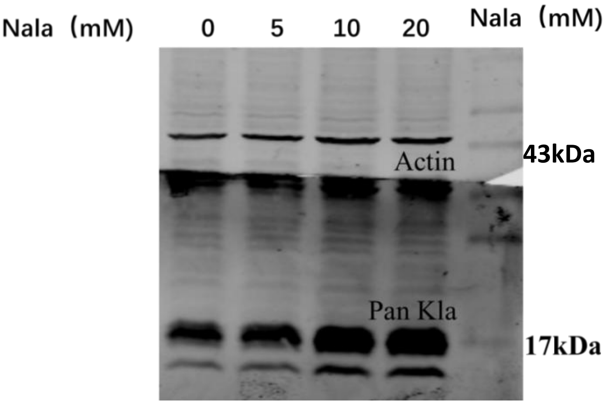

2、

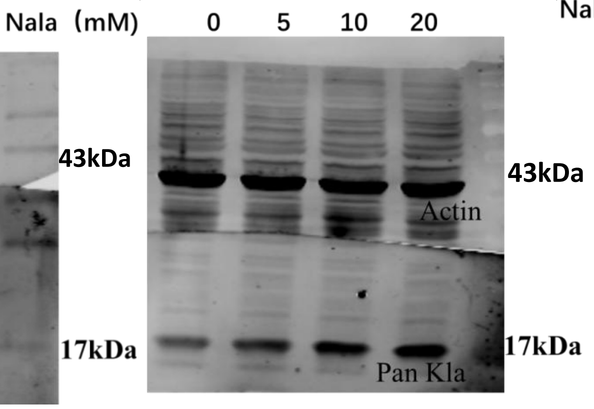

3、

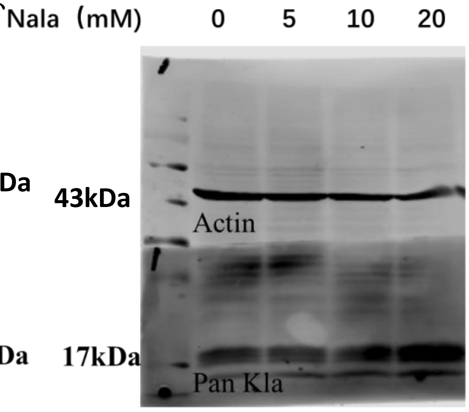

H3K18la，n=4

1、

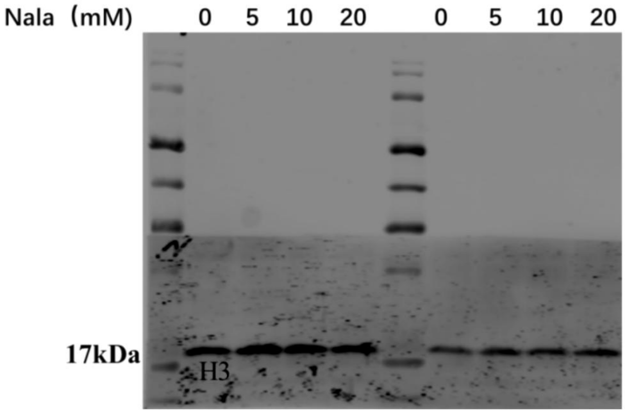

2、

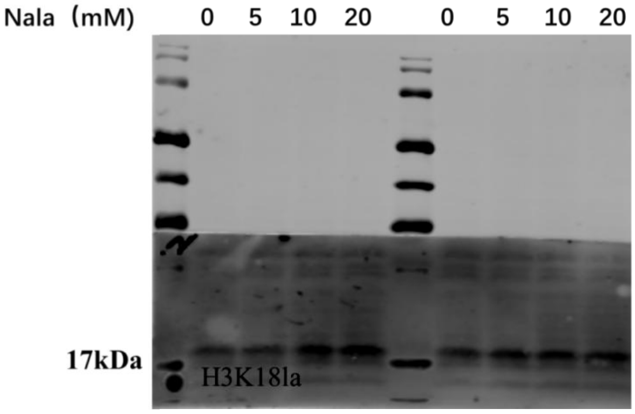

3、

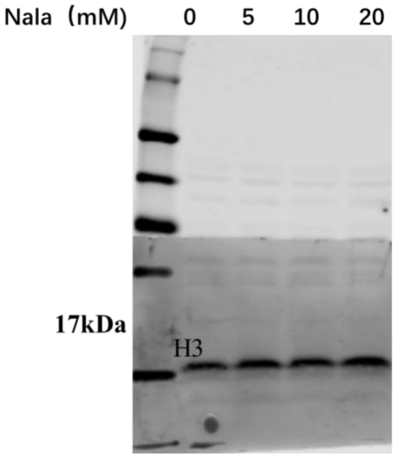

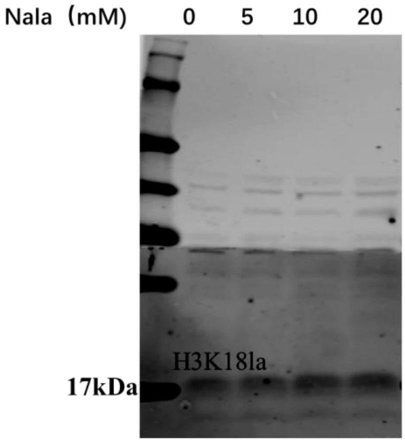

4、

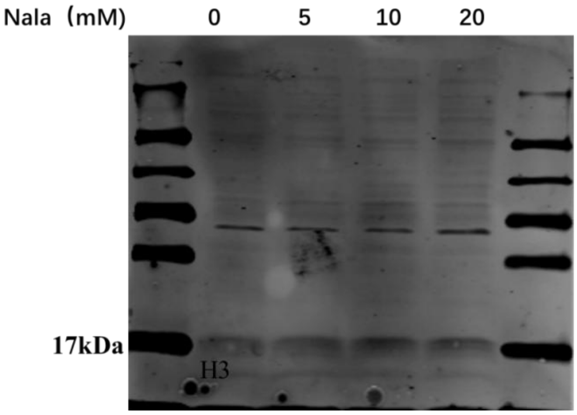

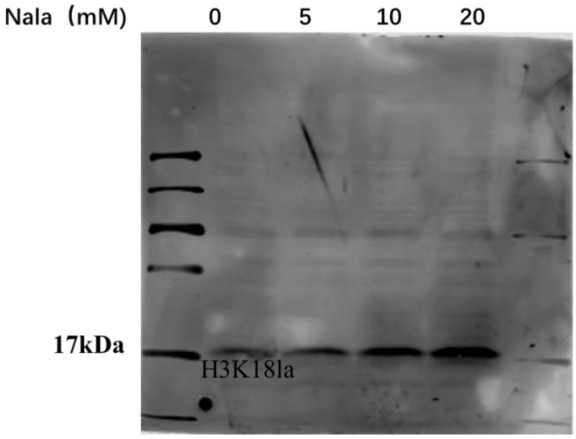

Figure2A:

Pan Kla，n = 5

1、 2、

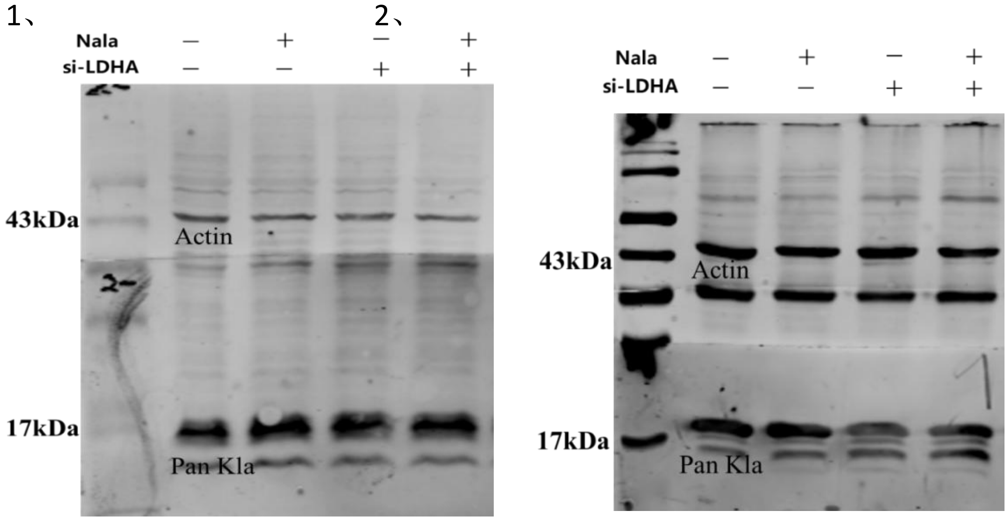

3、 4、 5、

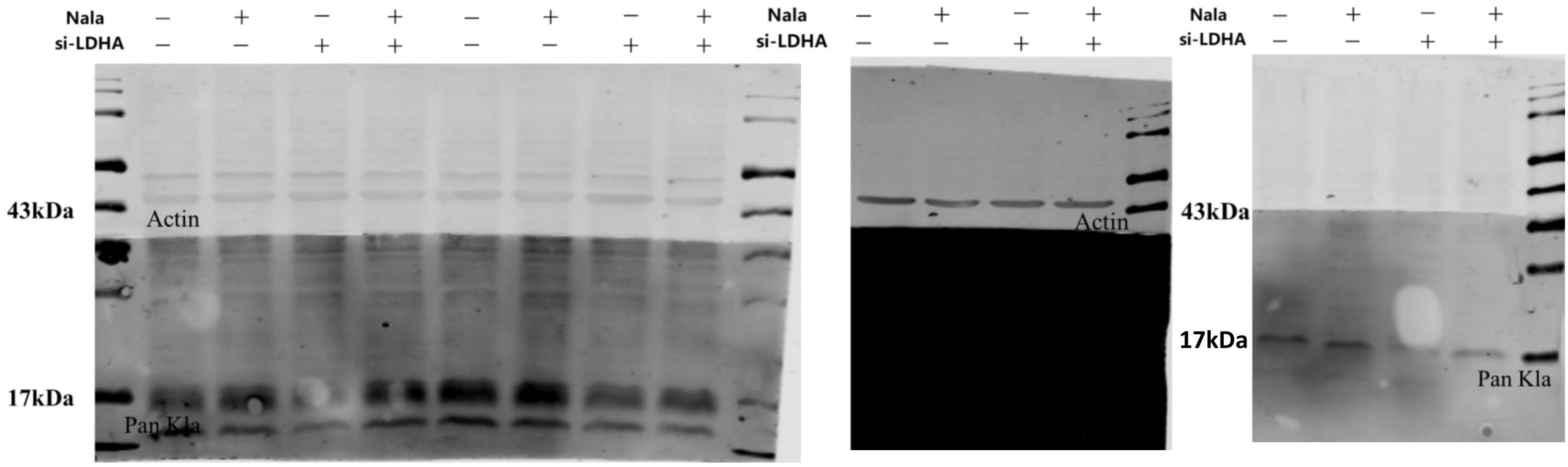

LDHA，n = 4

1、 2、 3、 4、

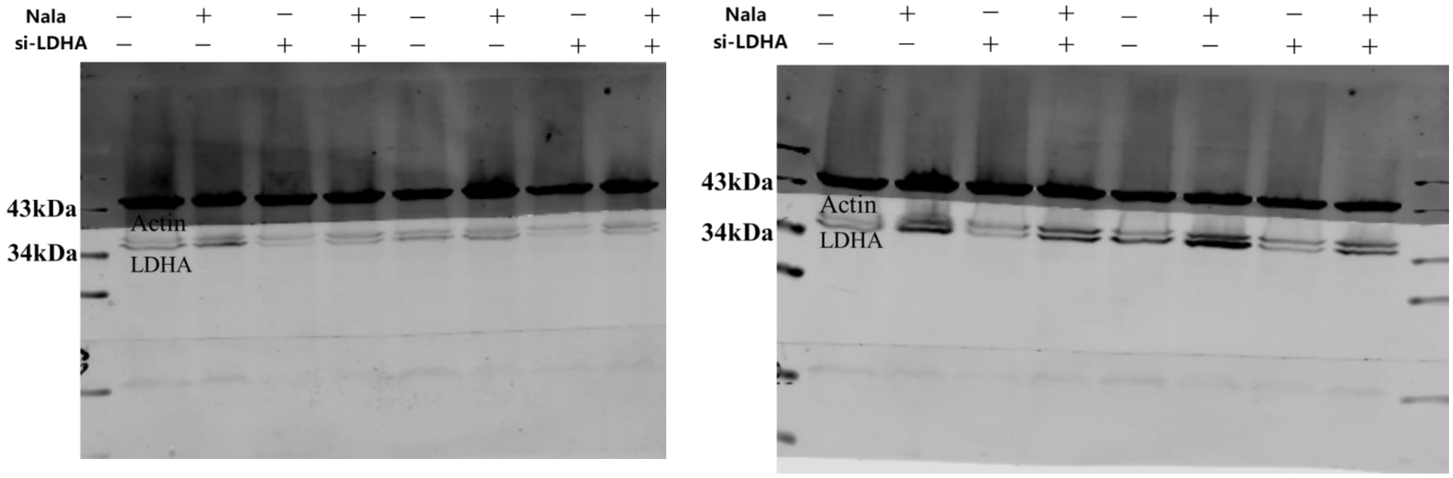

H3K18la，n=4

1、

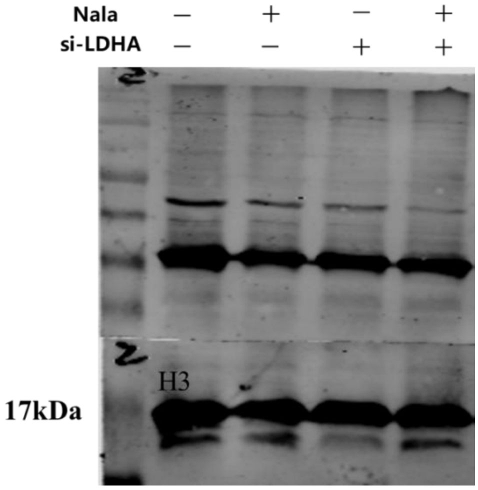

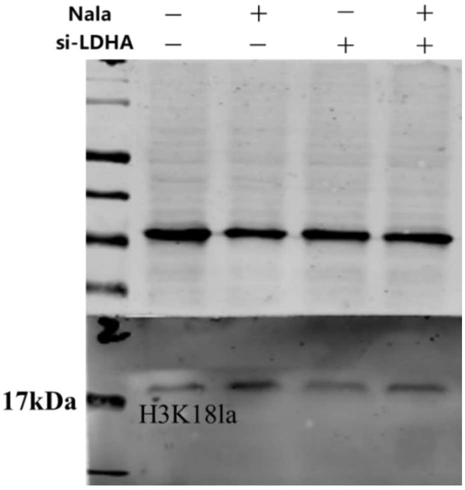

2、 3、 4、

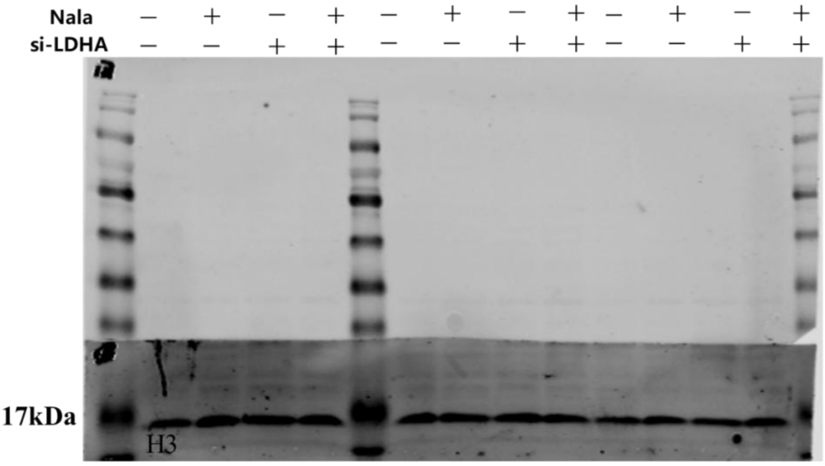

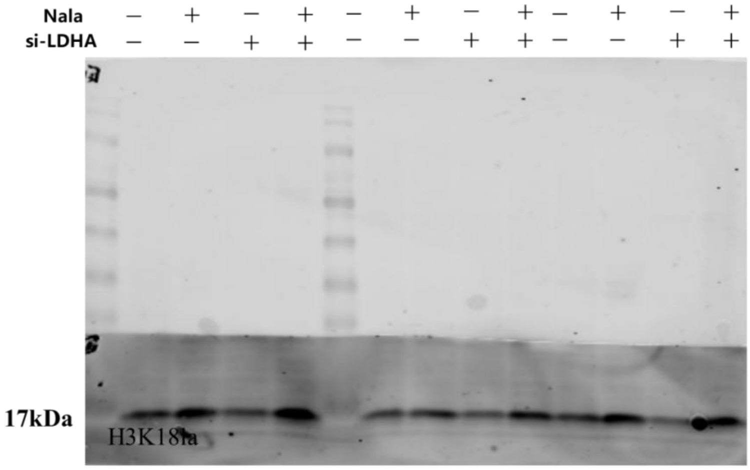

Bcl2，n=5

1、 2、

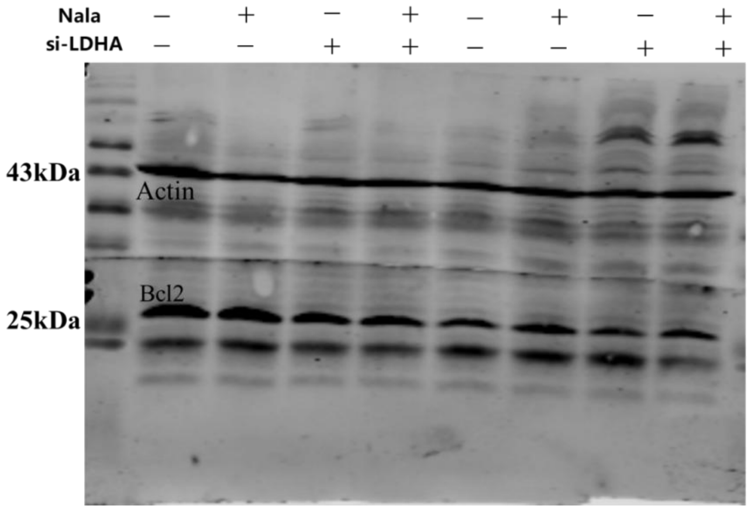

3、 4、

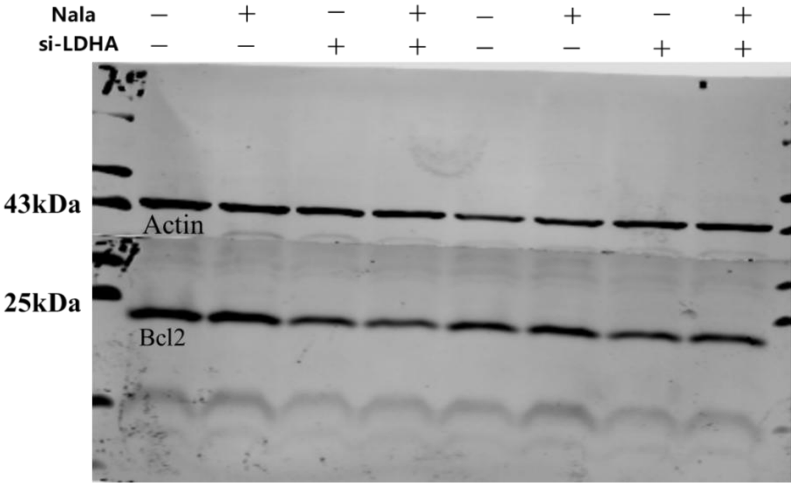

5、

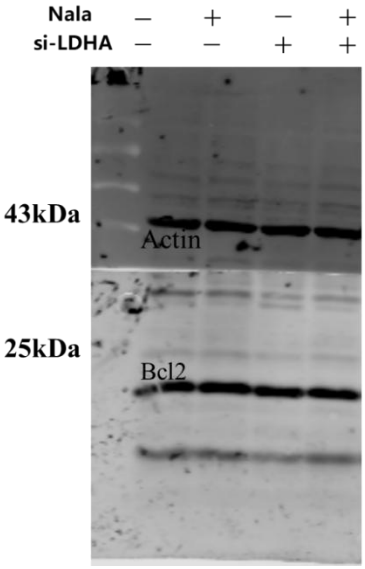
Nala – + – +
si-LDHA – – + +
43kDa
Actin
25kDa
Bcl2

Bax，n=3

1、 2、

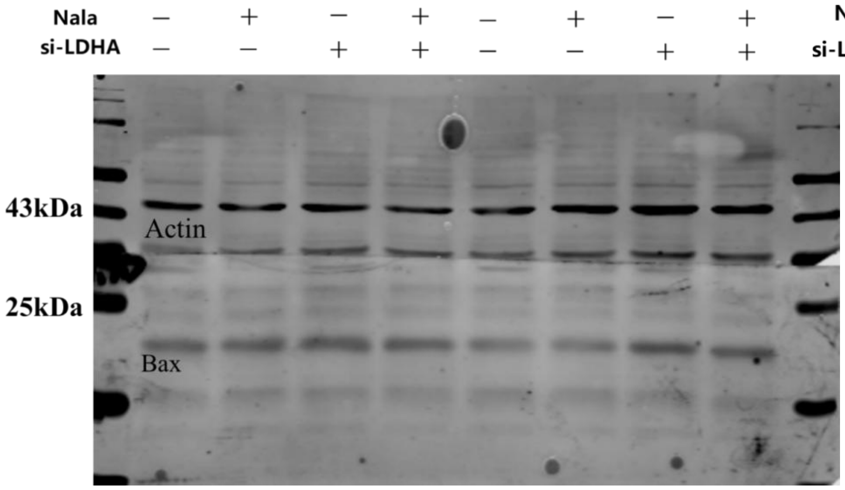
Nala – + – + – + – +
si-LDHA – – + + – – + +
43kDa
Actin
25kDa
Bax

3、

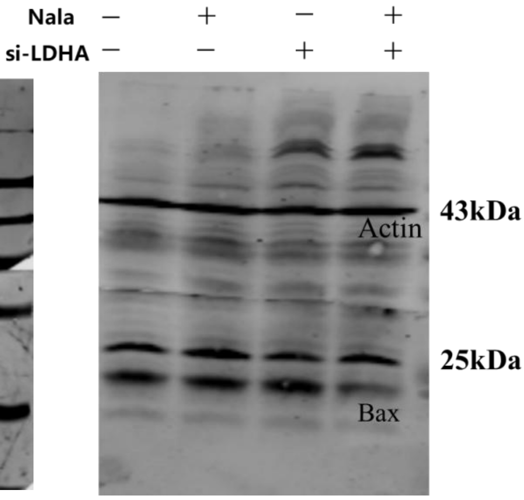
Nala – + – +
si-LDHA – – + +
43kDa
Actin
25kDa
Bax

Figure2B:

Pan Kla，n = 7

1、

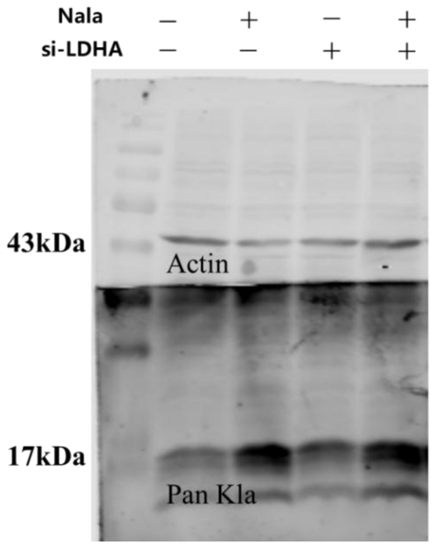

2、

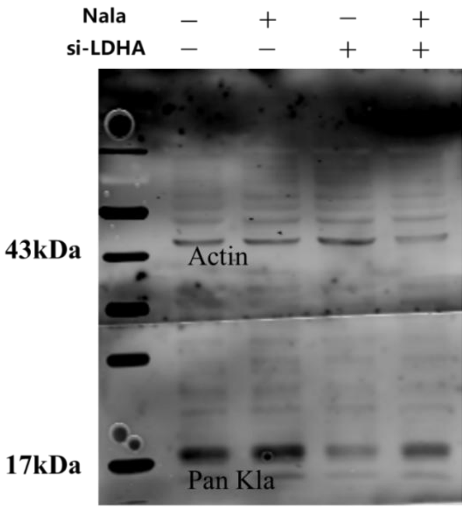

3、

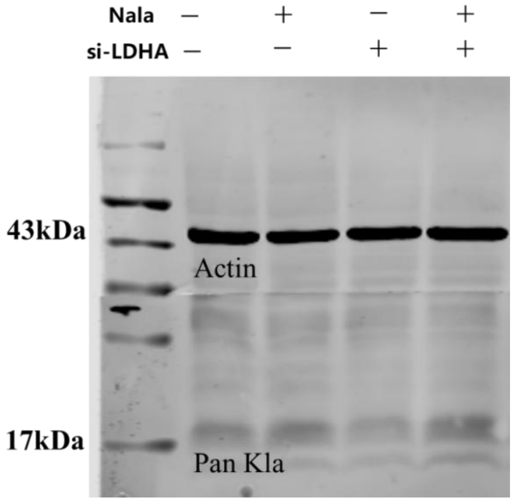

4、 5、

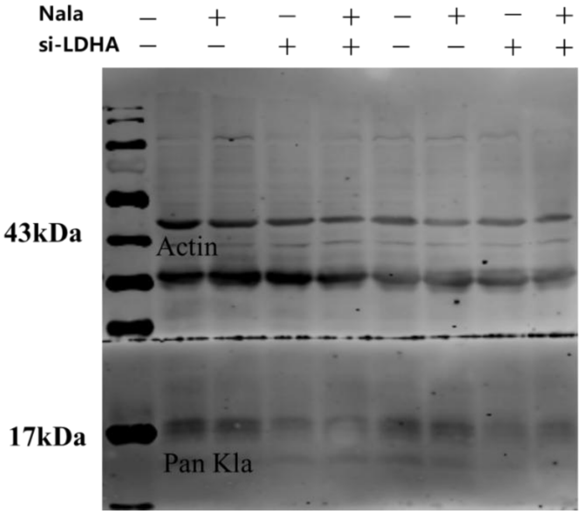

6、 7、

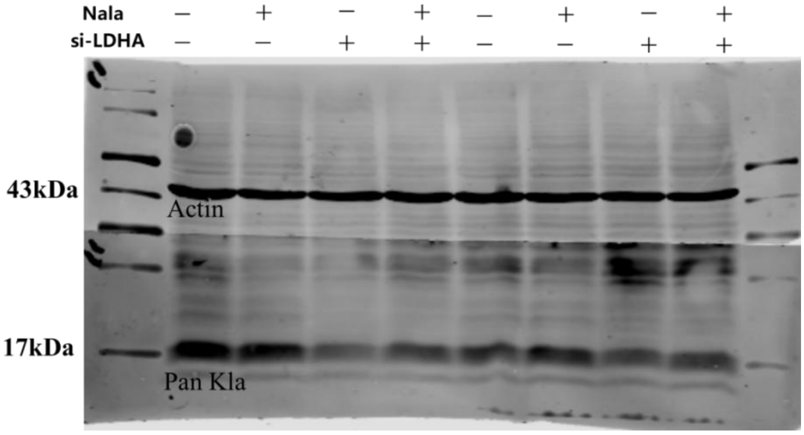

LDHA，n = 4

1、 2、

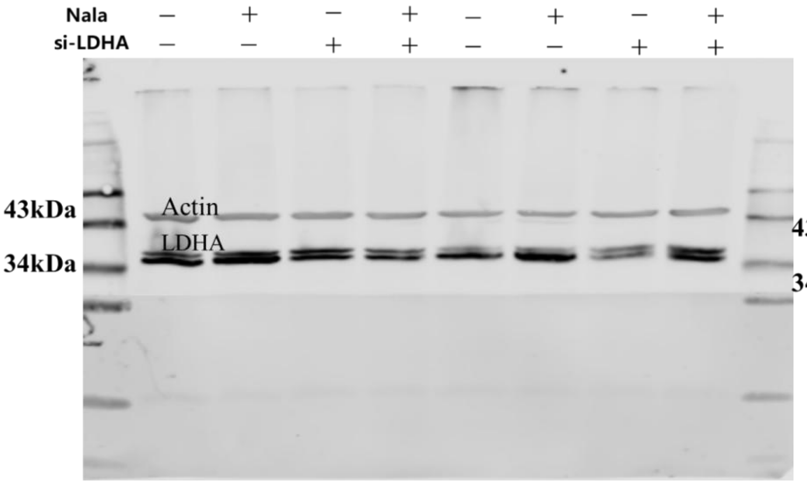

3、 4、

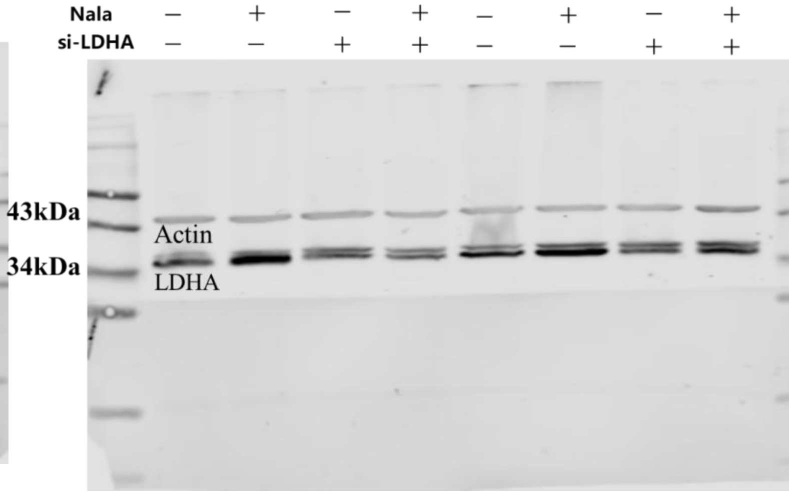

H3K18la，n=3

1、 2、

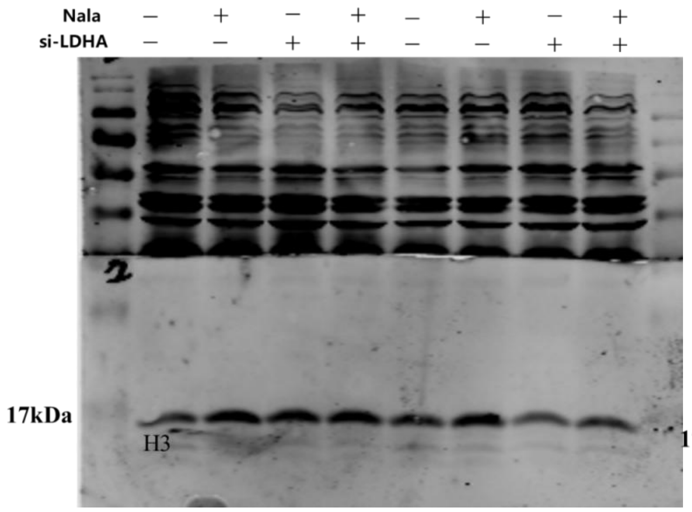

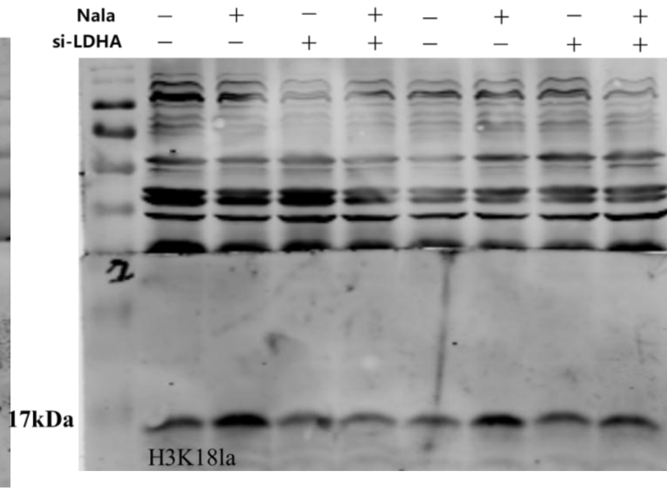

3、

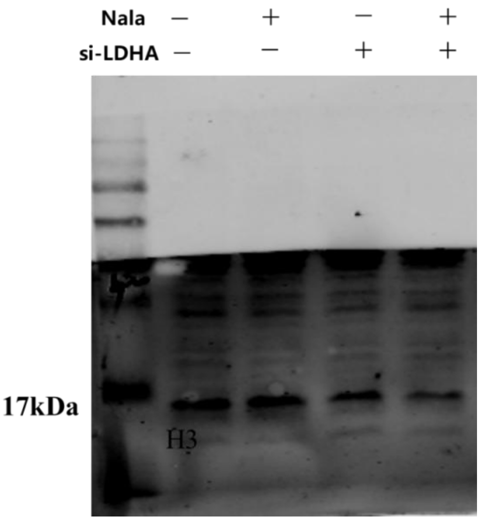

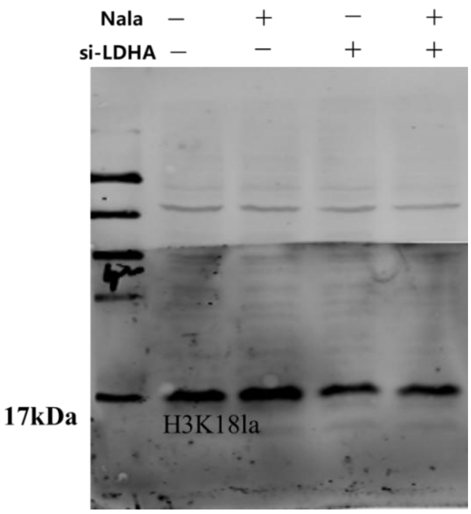

Bcl2，n=4

1、 2、 3、 4、

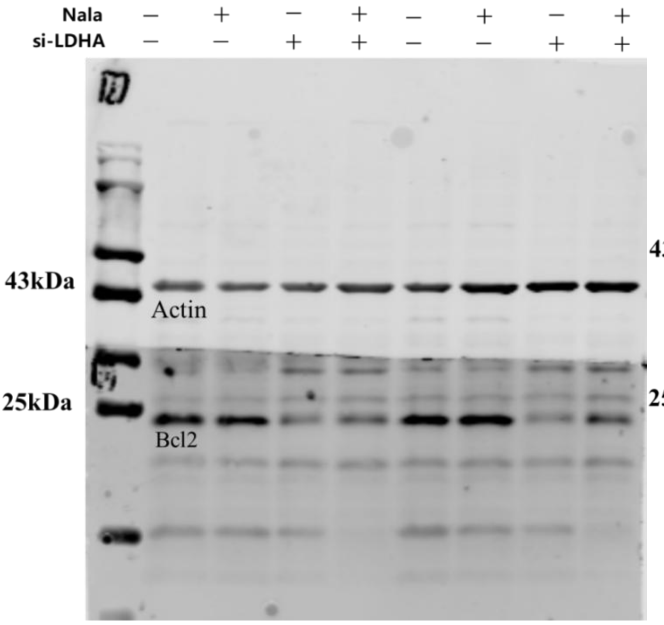

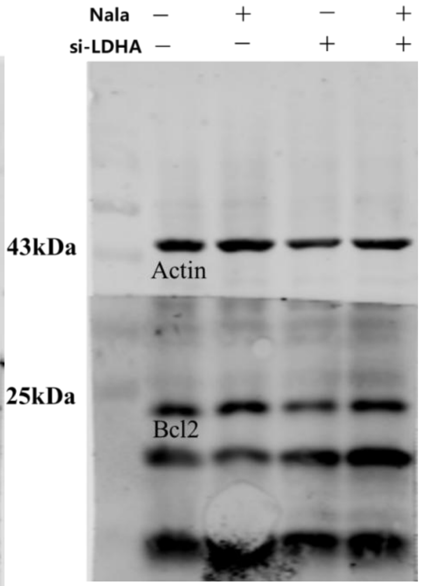

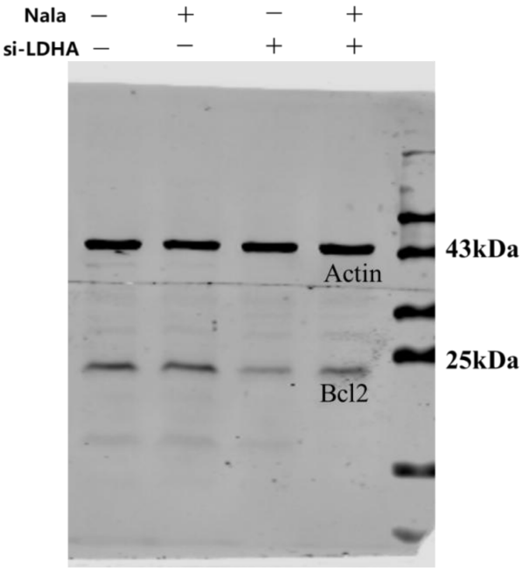

Bax，n=3

1、

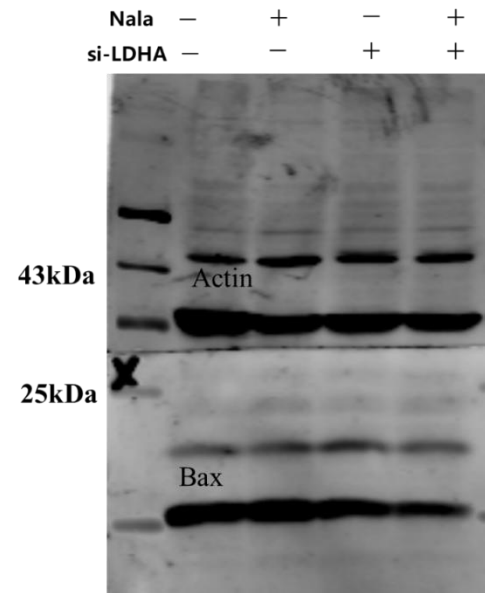
Nala — + — +
si-LDHA — — + +
43kDa
Actin
25kDa
Bax

2、

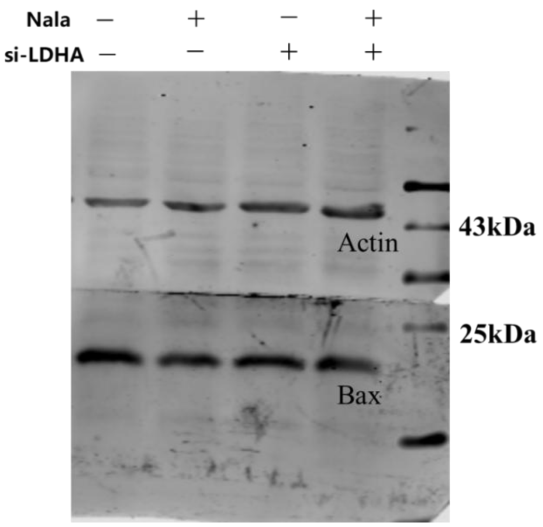
Nala — + — +
si-LDHA — — + +
43kDa
Actin
25kDa
Bax

3、

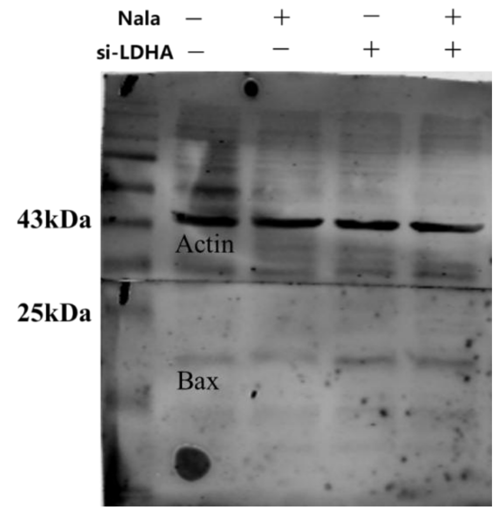
Nala — + — +
si-LDHA — — + +
43kDa
Actin
25kDa
Bax

Figure3B:

Pan Kla，n = 4

1、

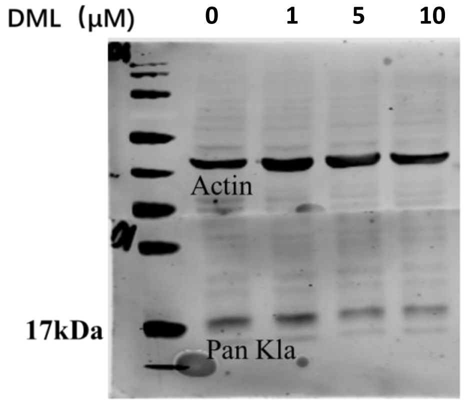

2、

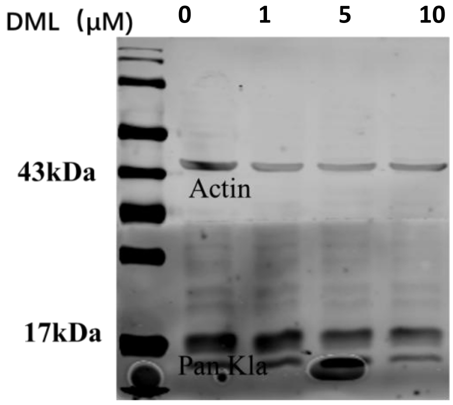

3、

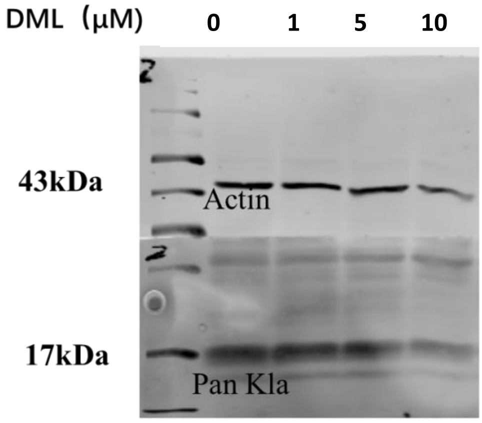

4、

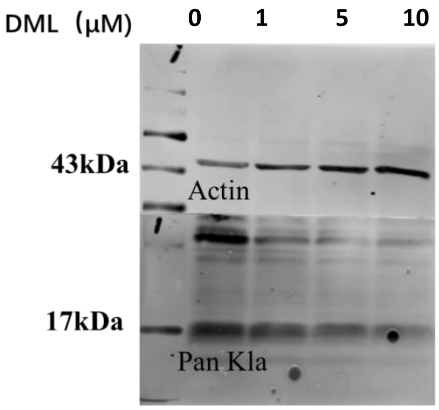

LDHA，n = 3

1、

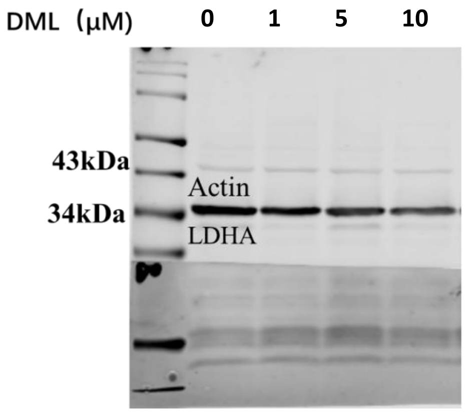

2、

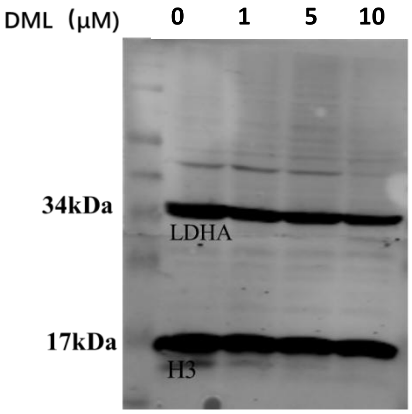

3、

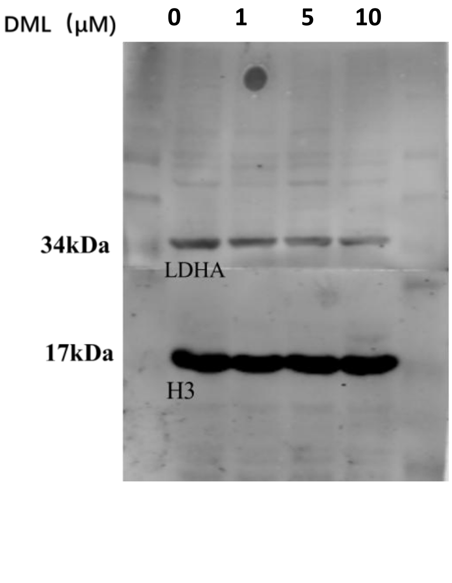

H3K18la，n=5

1、

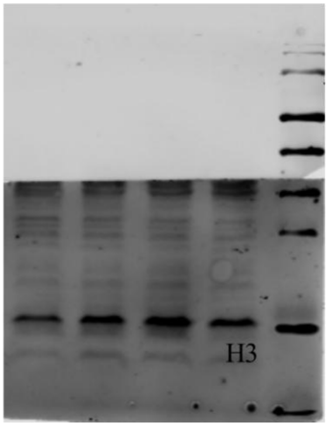

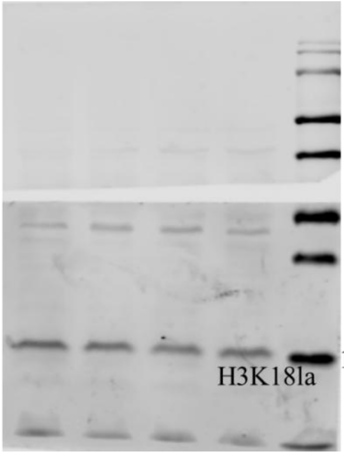

2、

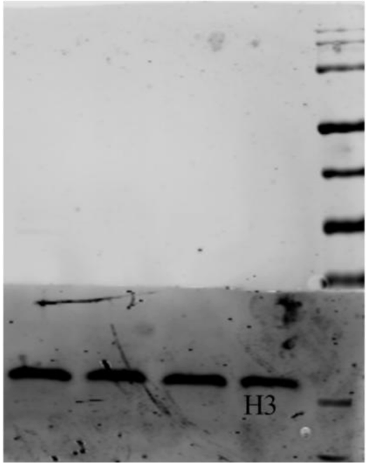

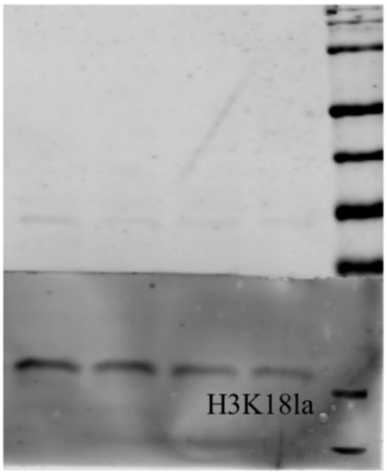

3、

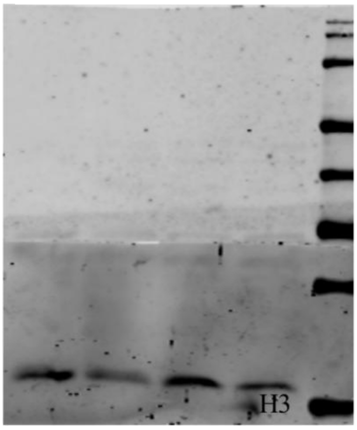

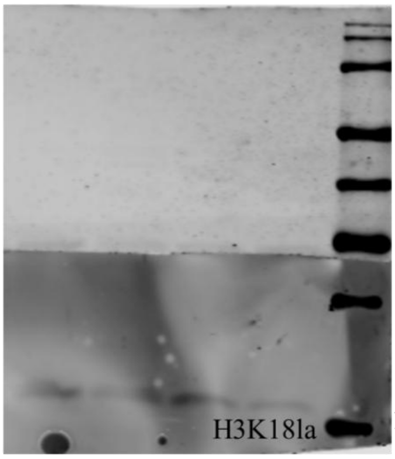

4、

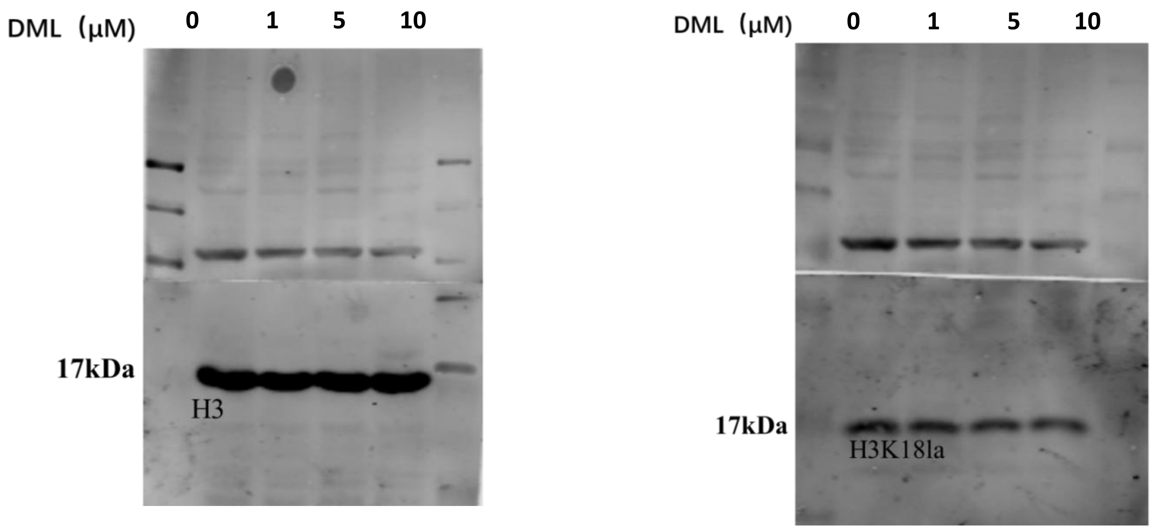
DML（μM） 0 1 5 10
17kDa
H3
DML（μM） 0 1 5 10
17kDa
H3K18la

5、

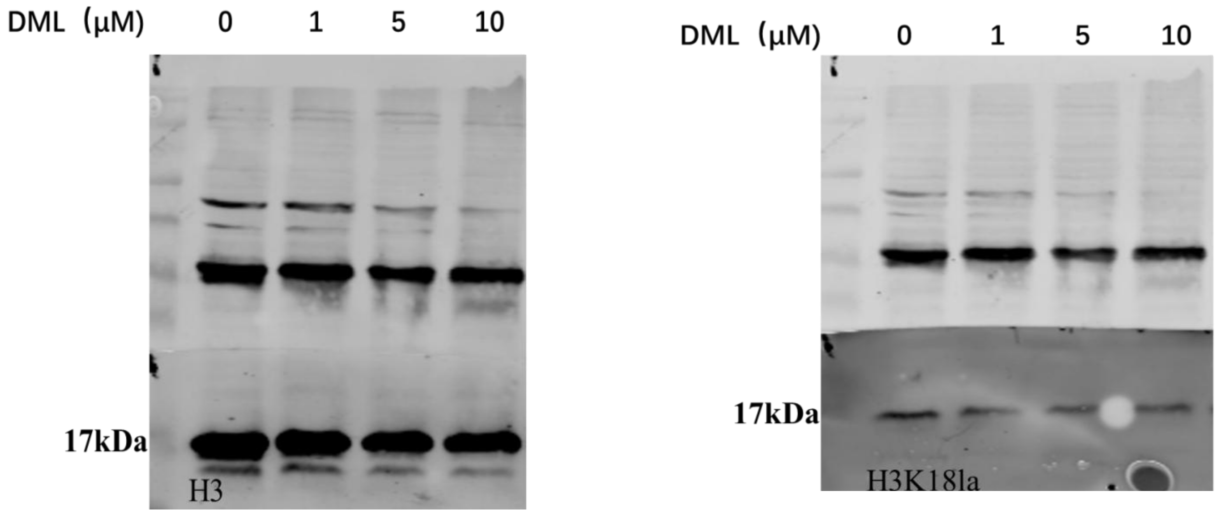
DML（μM） 0 1 5 10
17kDa
H3
DML（μM） 0 1 5 10
17kDa
H3K18la

Figure3F:

Pan Kla，n = 3

1、

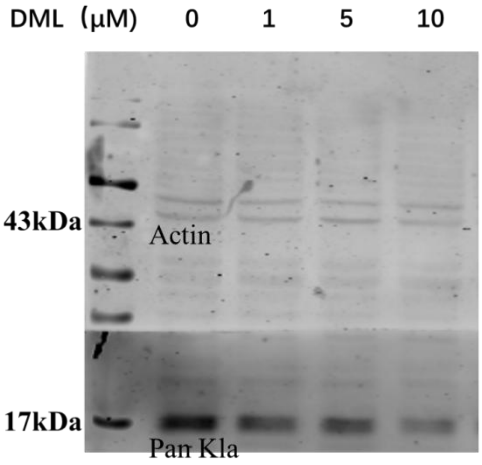

2、

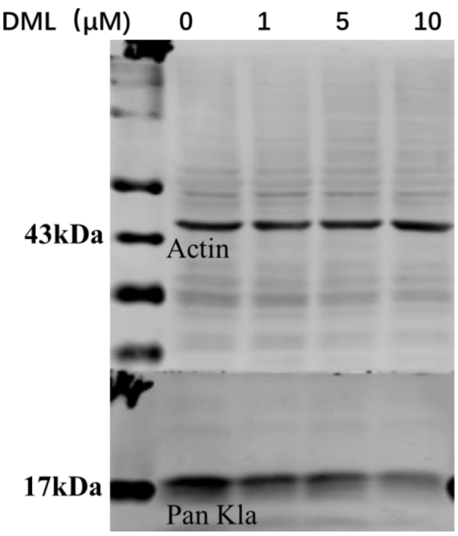

3、

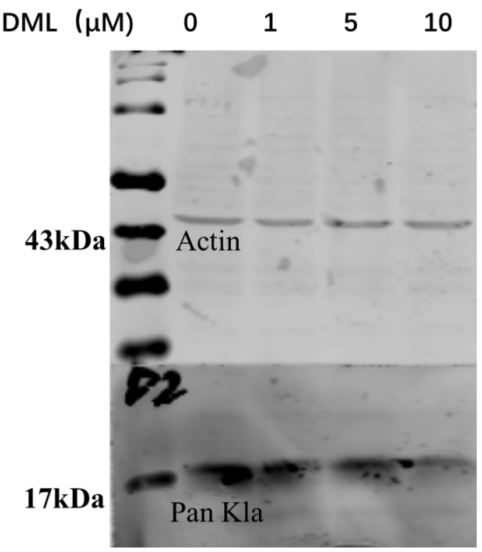

LDHA，n = 3

1、

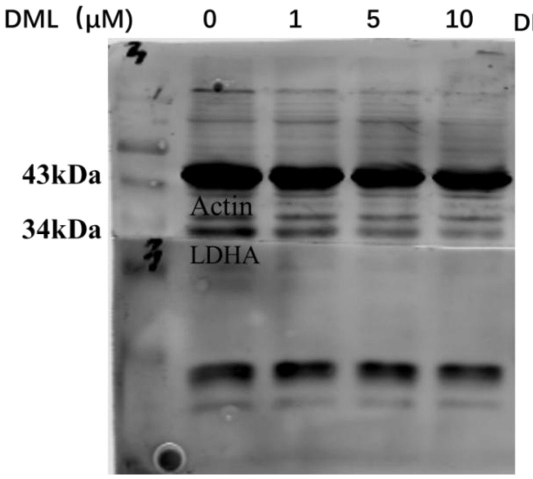

2、

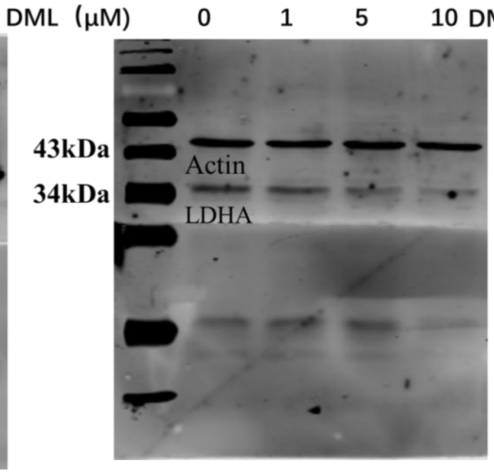

3、

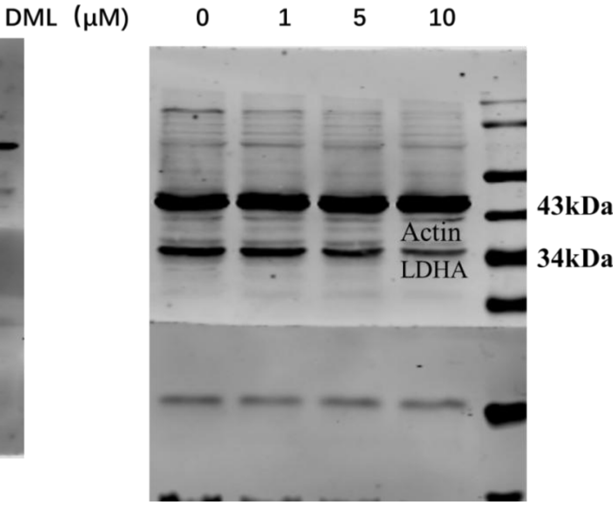

H3K18la，n=3

1、

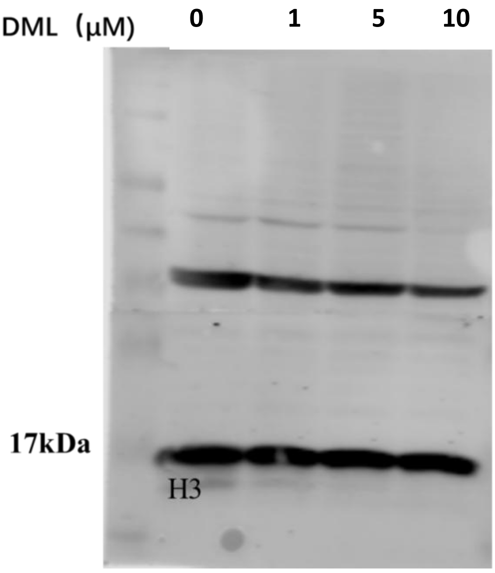

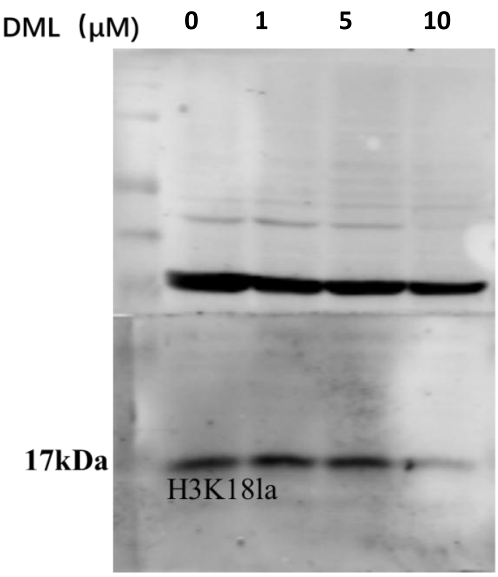

2、 3、

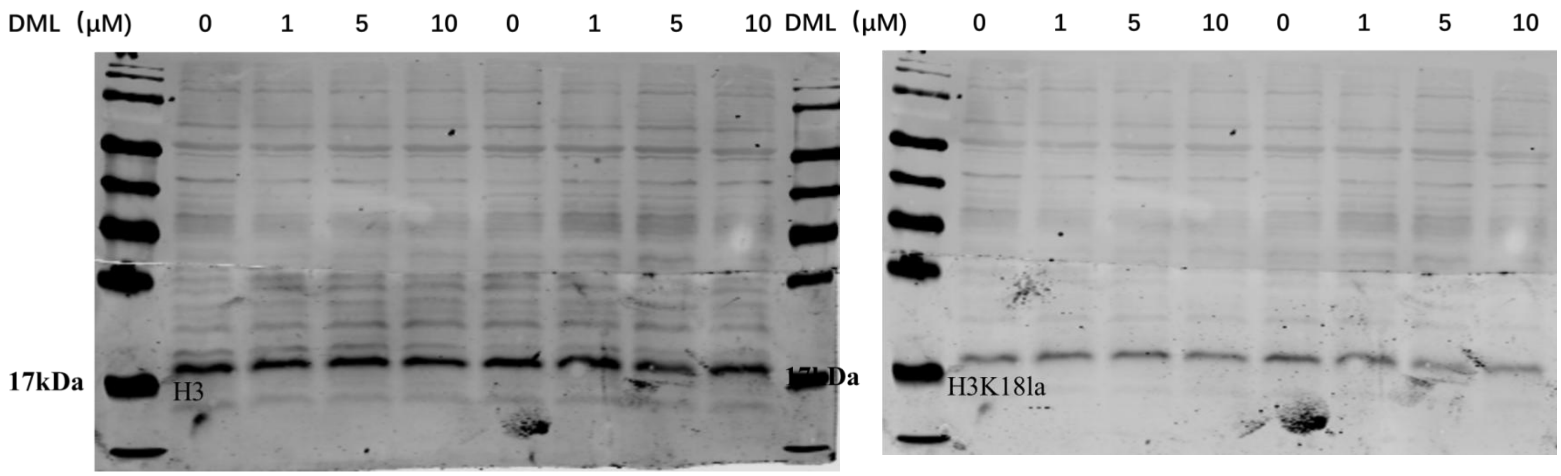

Figure3J:

Pan Kla，n = 5

1、 2、 3、

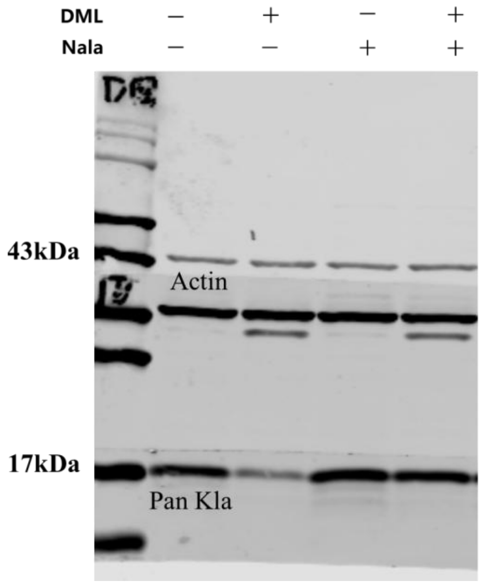

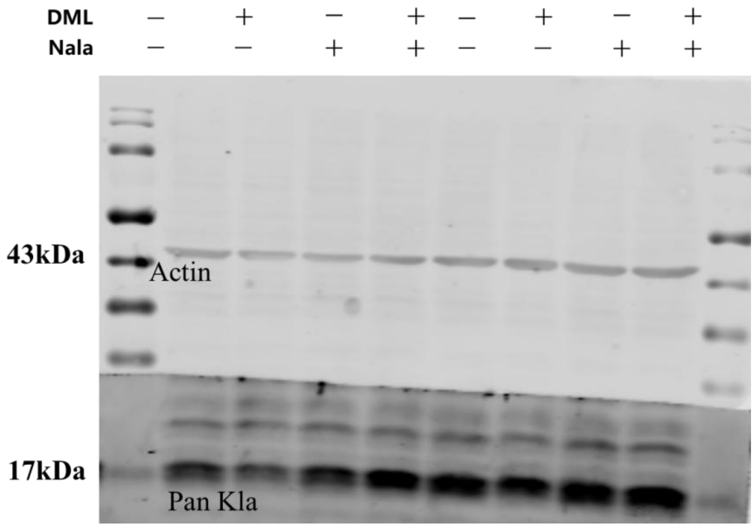

4、 5、

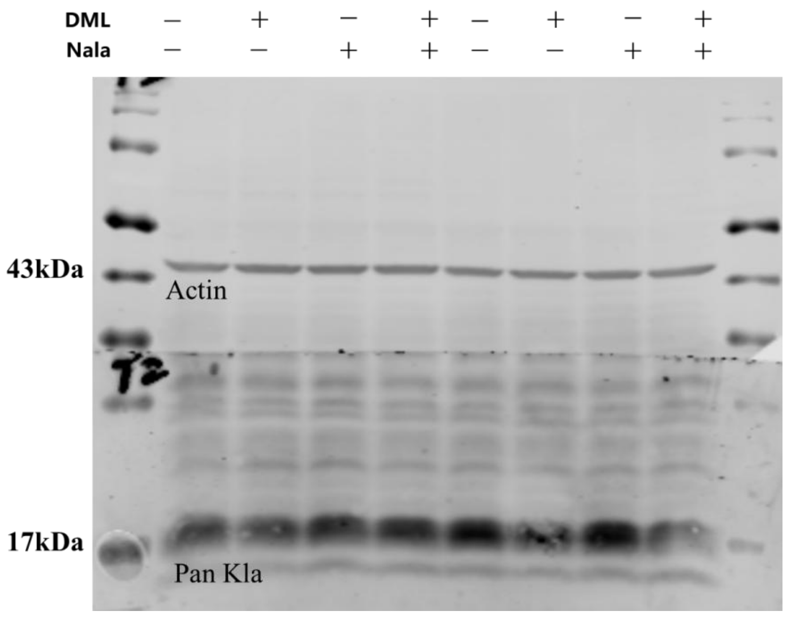

LDHA，n = 3

1、

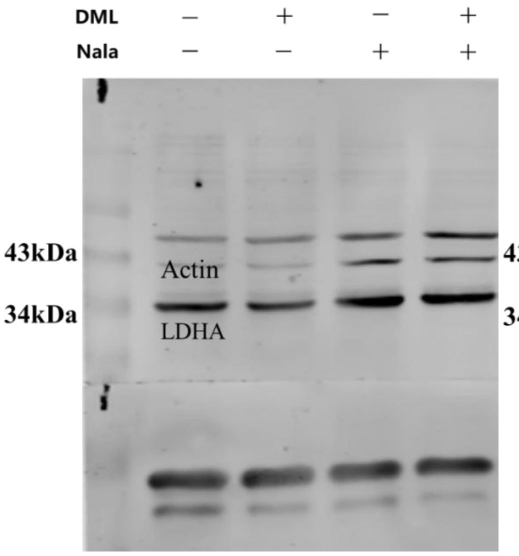

2、

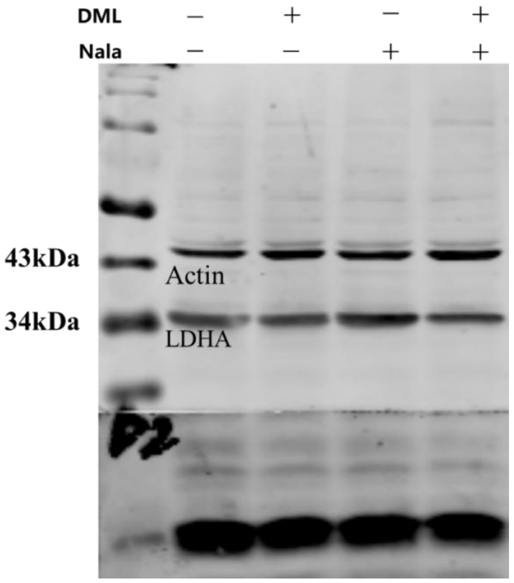

3、

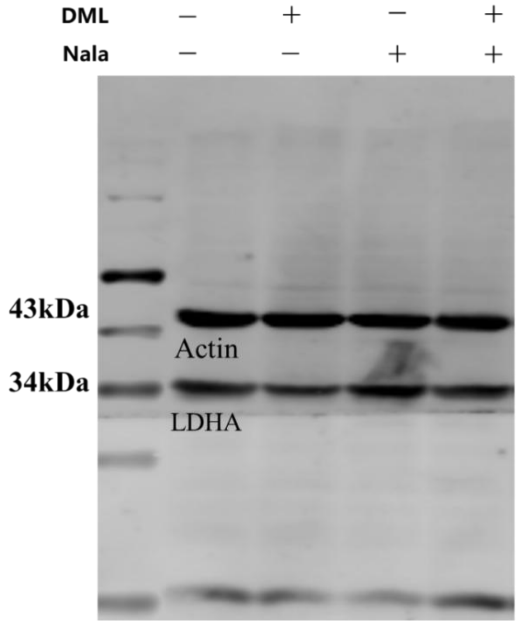

H3K18la，n=3

1、 2、

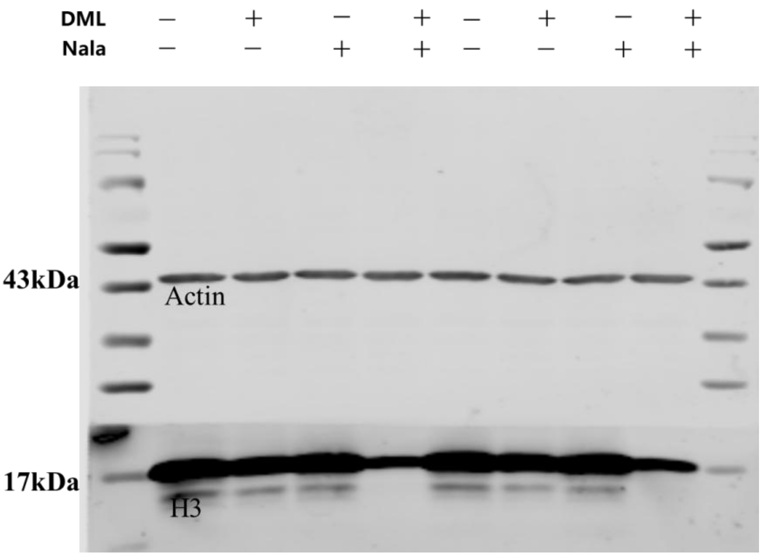

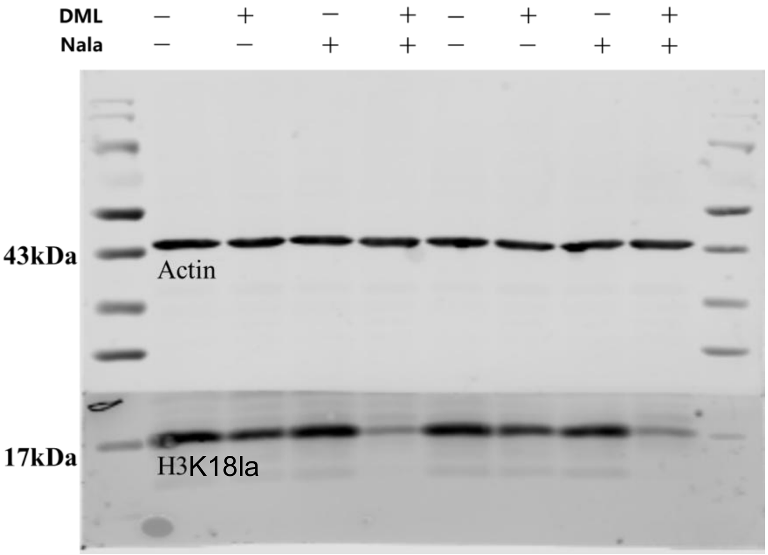

3、

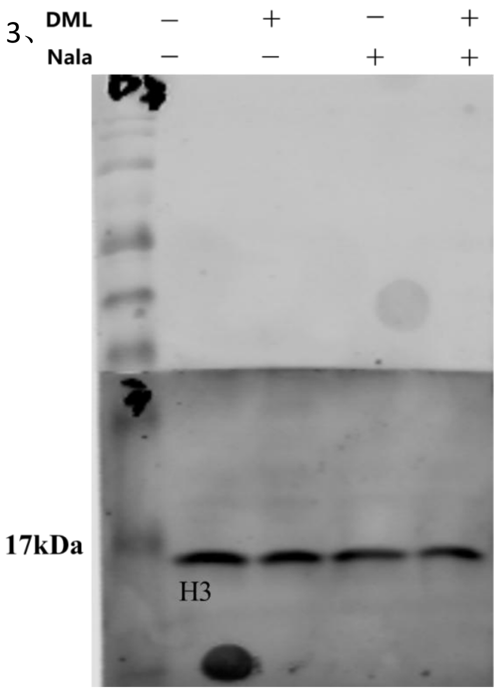

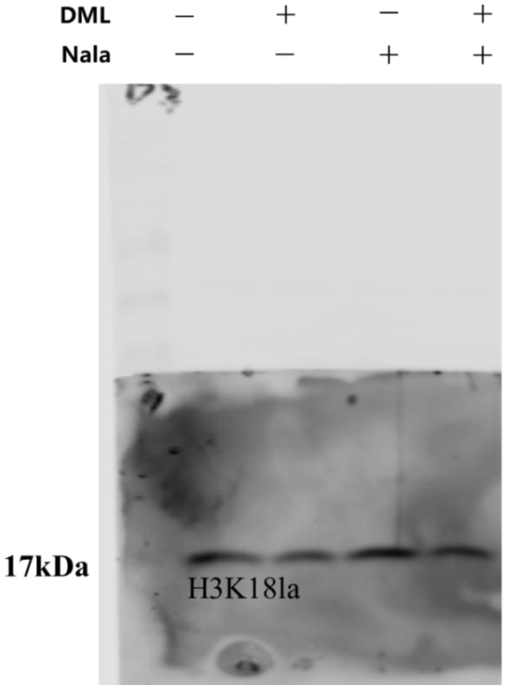

Bcl2，n=5

1、 2、

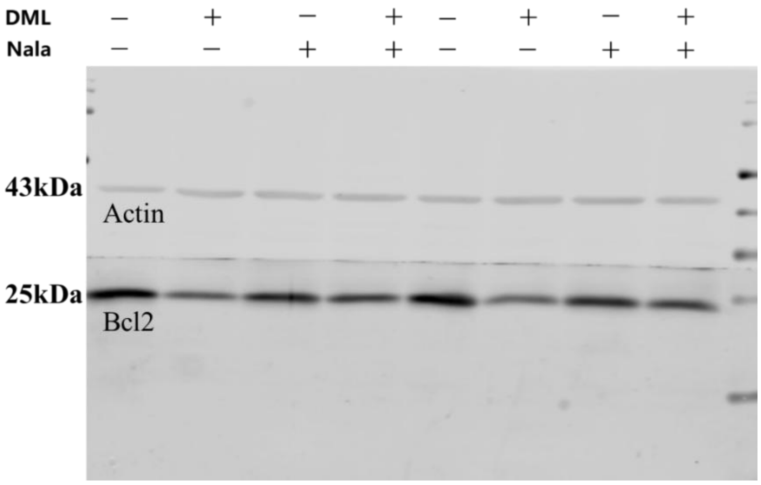

3、

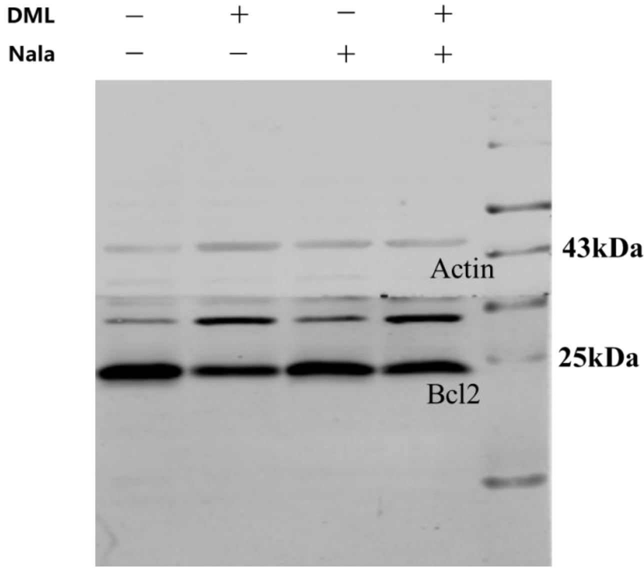

4、 5、

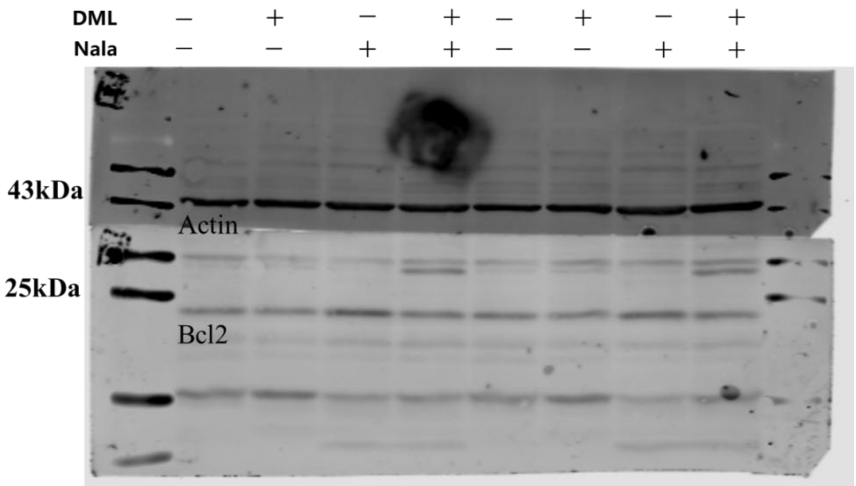

Bax，n=3

1、

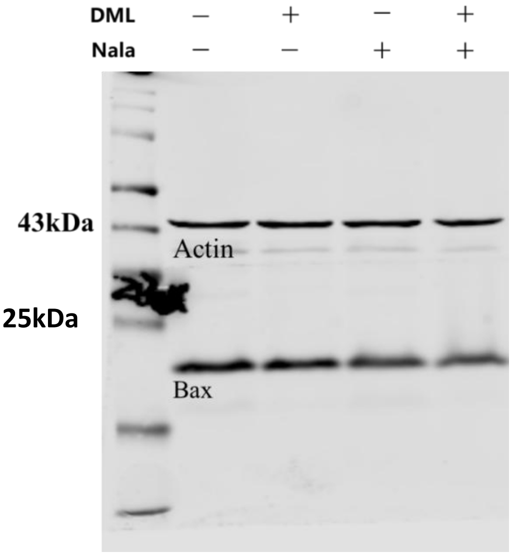

2、

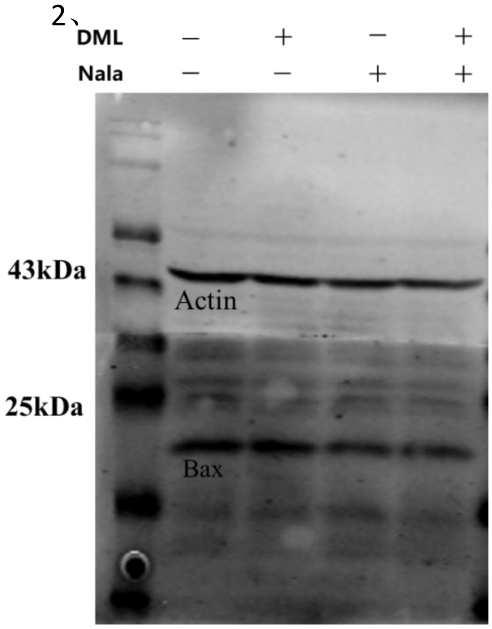

3、

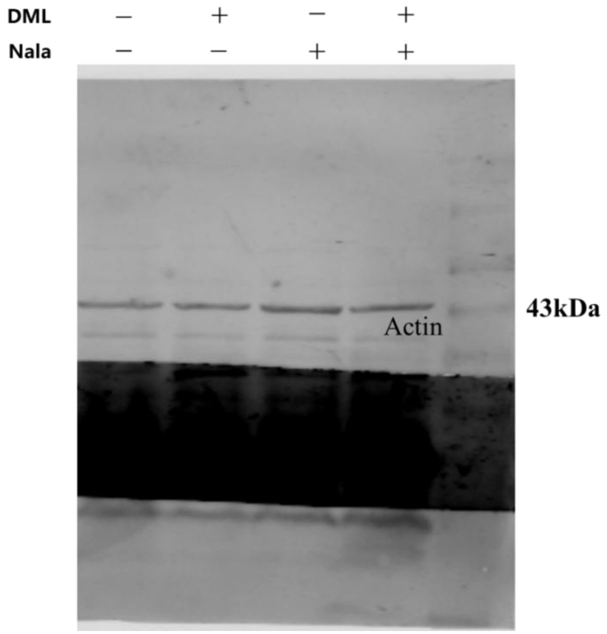

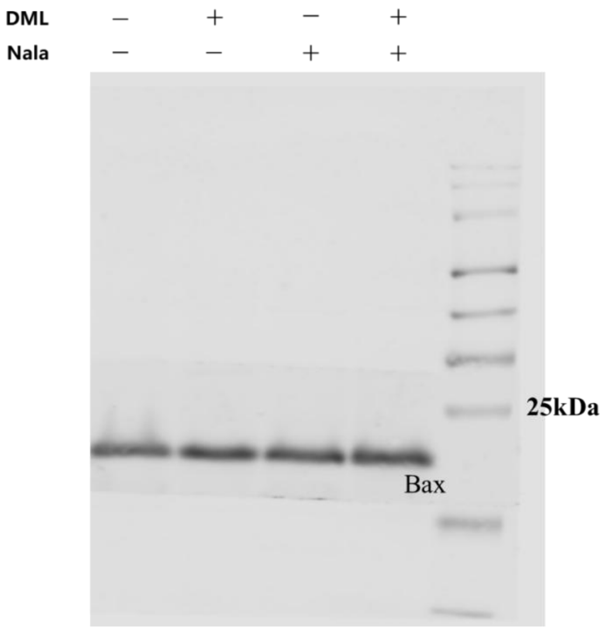

Figure3K:

Pan Kla，n = 5

1、 2、

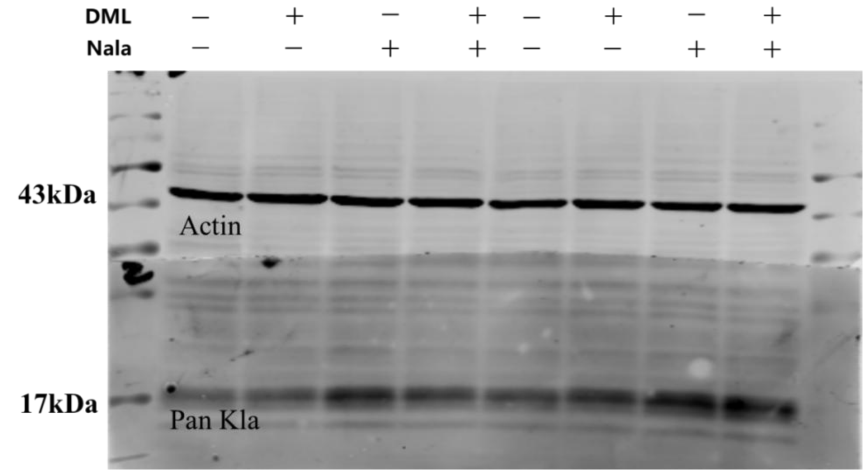

3、 4、

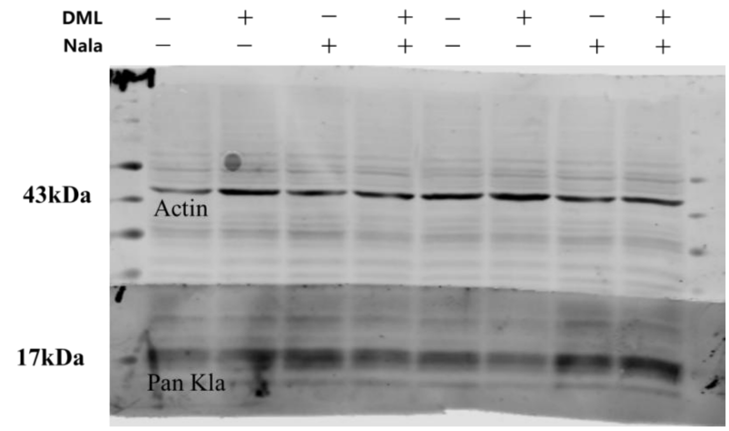

5、

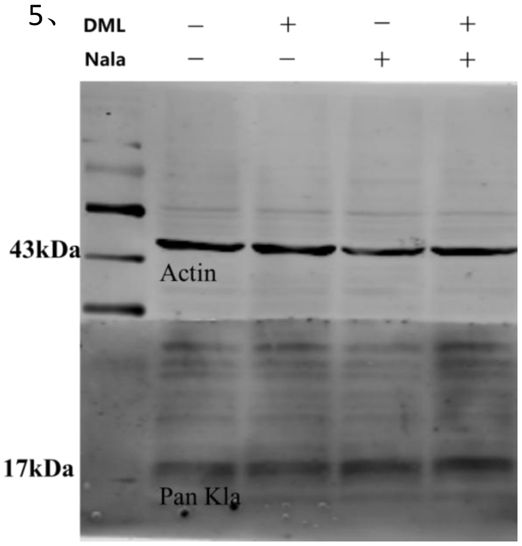

LDHA，n = 4

1、 2、

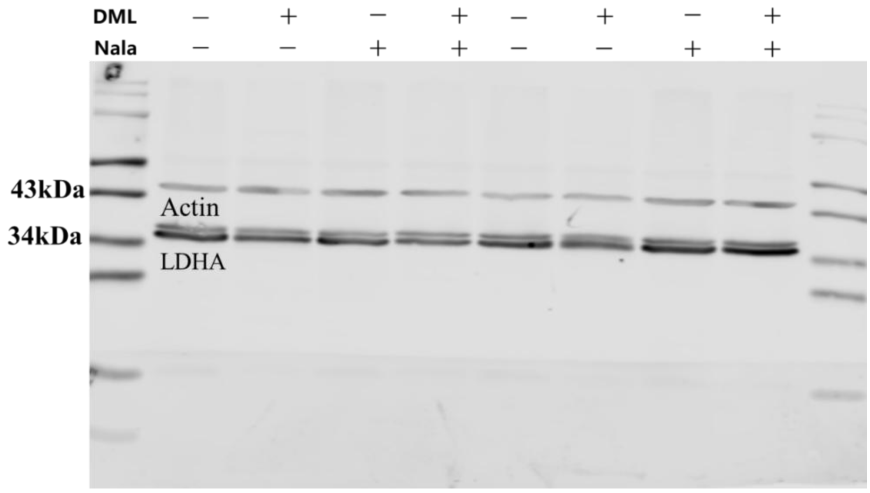

3、 4、

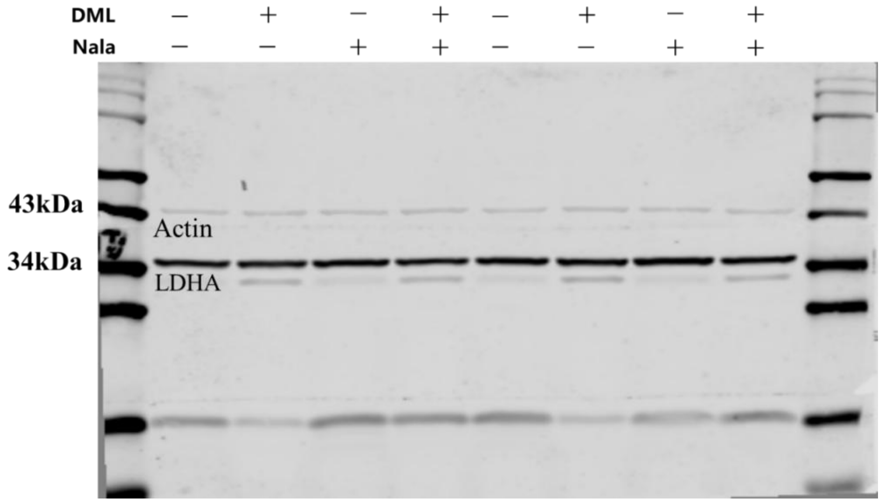

H3K18la，n=4

1、 2、

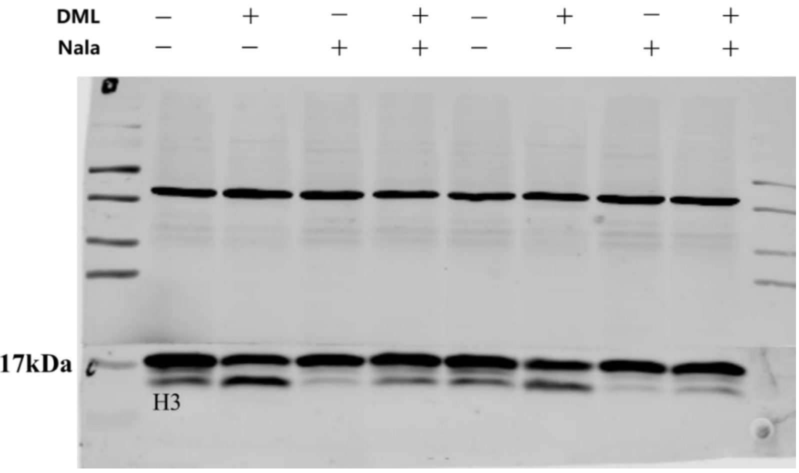

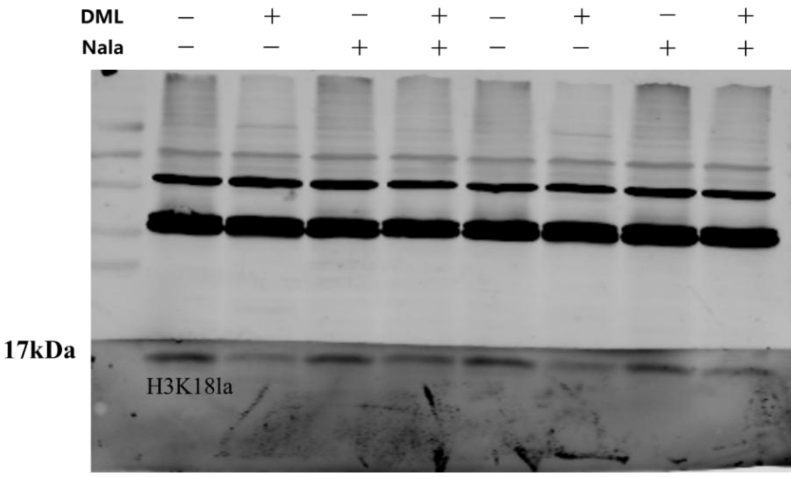

3、 4、

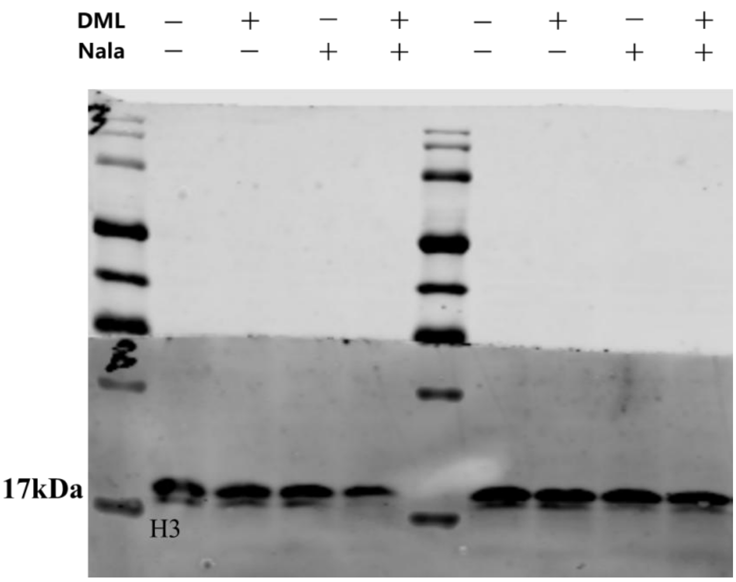

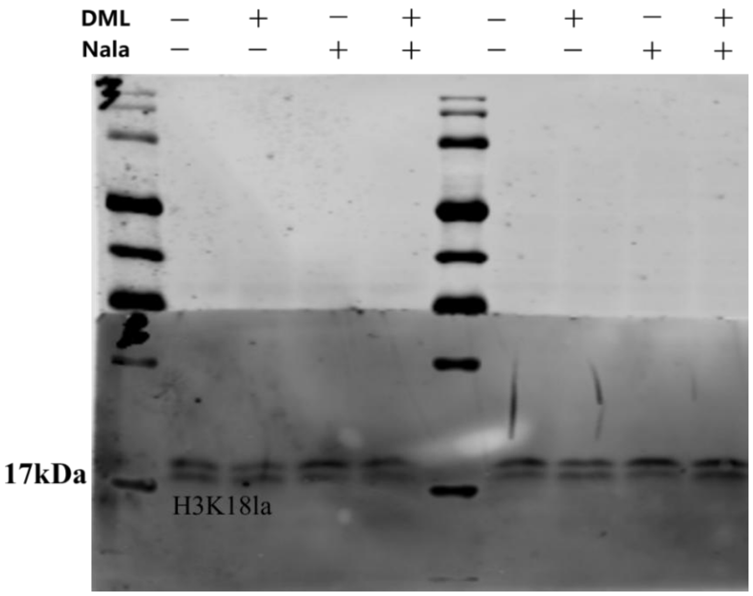

Bcl2，n=3

1、

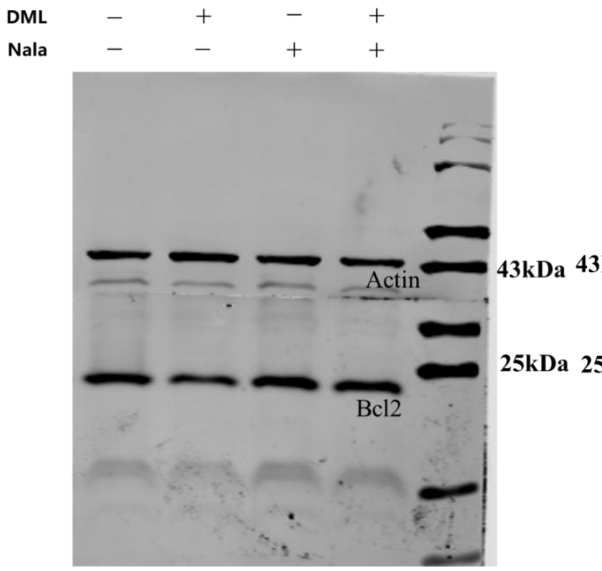

2、 3、

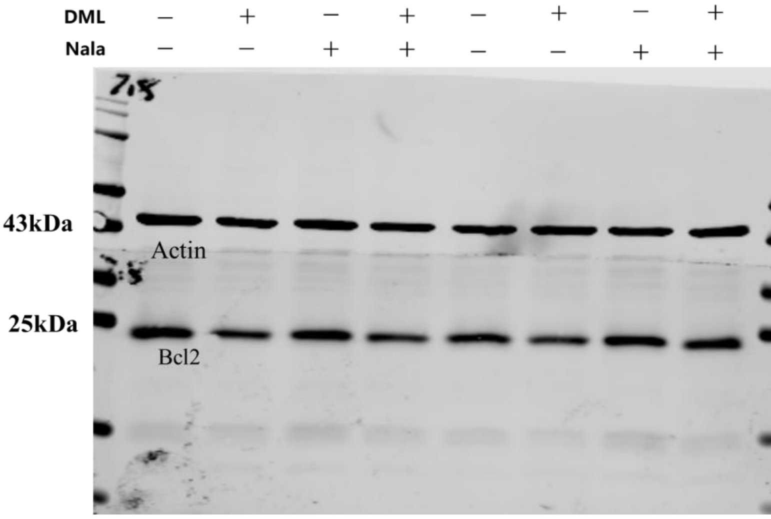

Bax，n=3

1、 2、

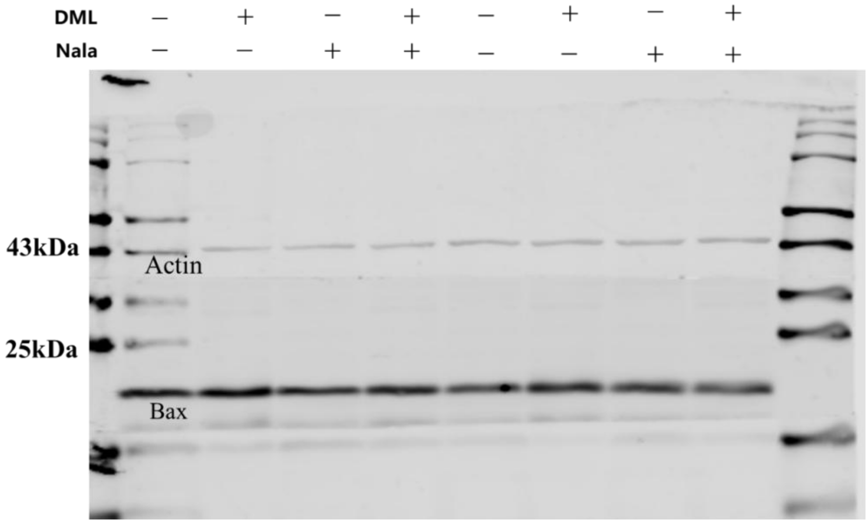

3、

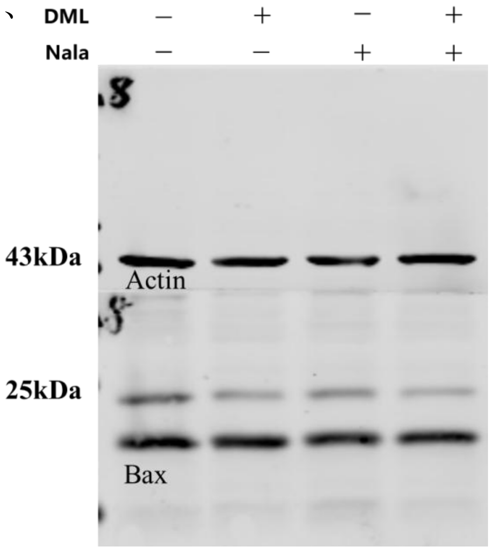

Figure4H:

MESP1，n = 9

1、 2、

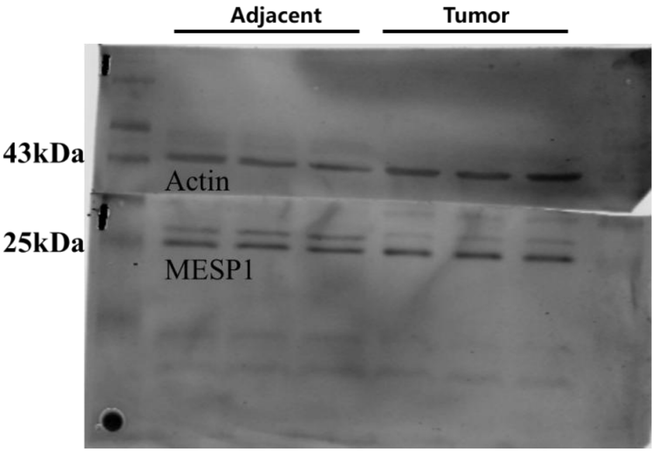

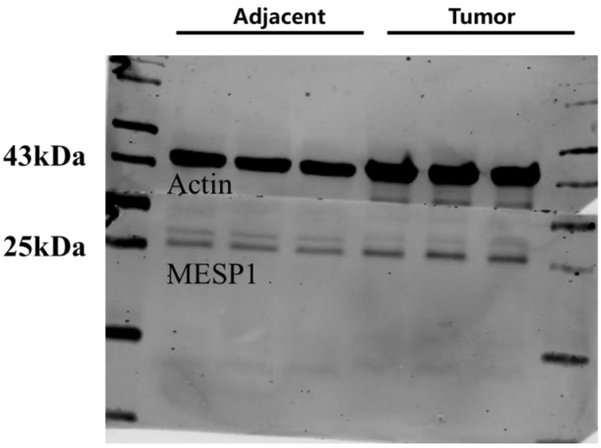

3、

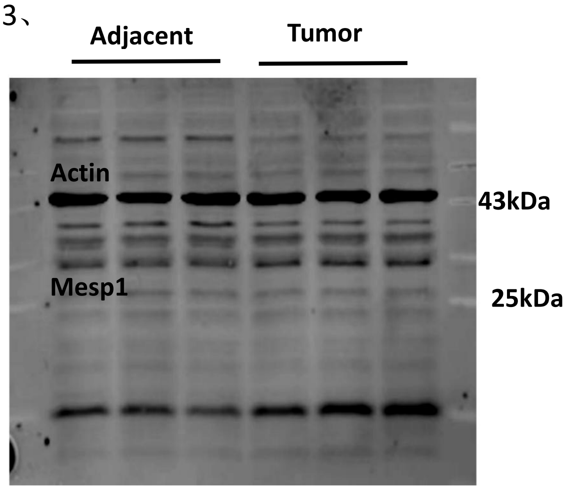

Figure4I:

MESP1，n = 3

1、 2、 3、

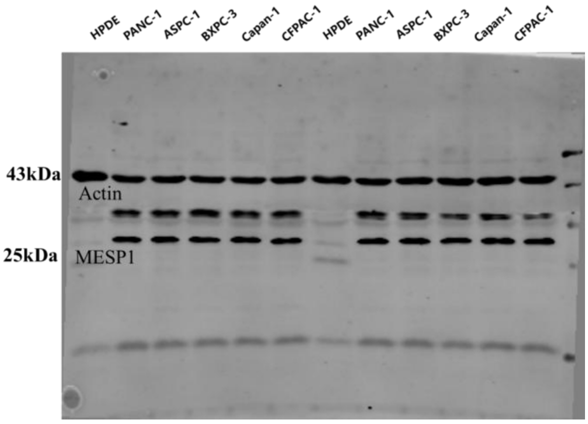

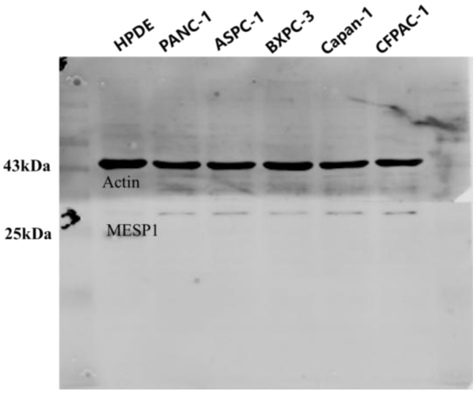

Figure4J: PANC-1

MESP1，n = 3

1、 2、

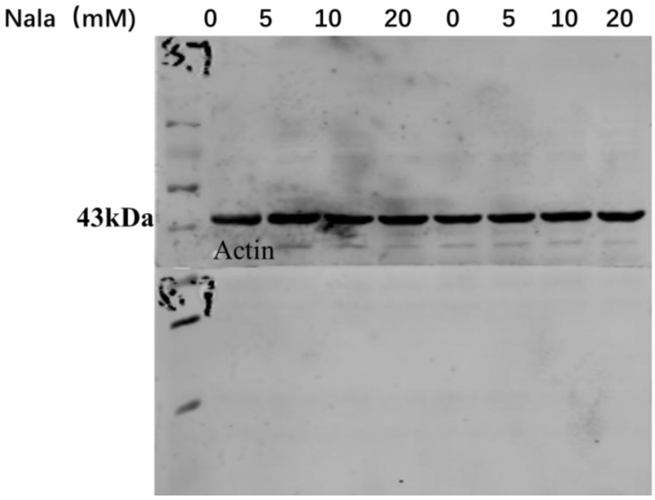

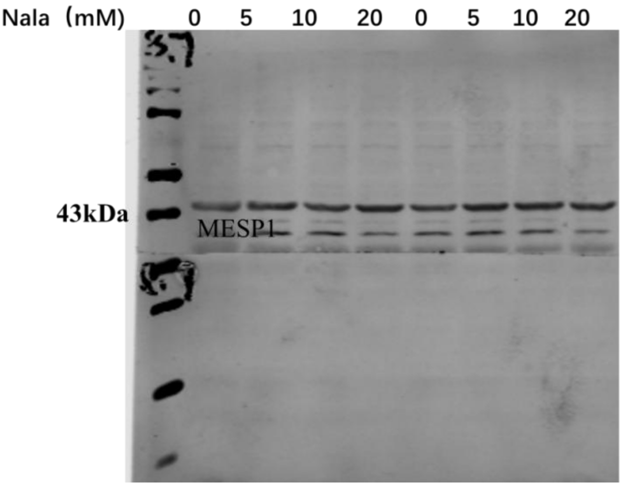

3、

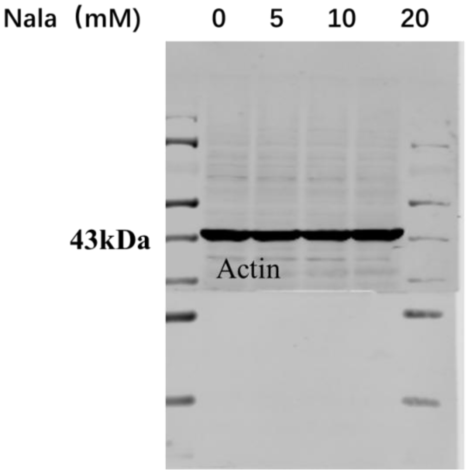

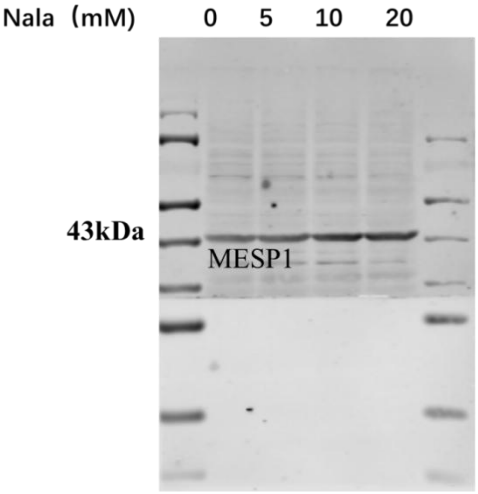

ASPC-1,MESP1，n = 3

1、

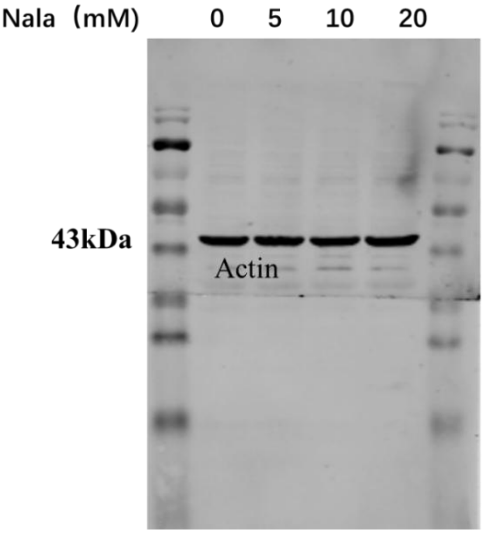

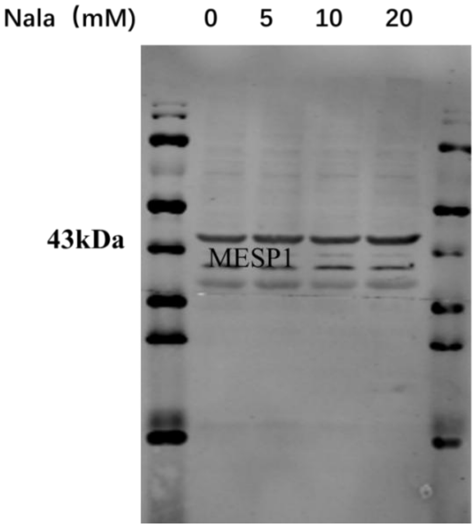

2、

Nala（mM） 0 5 10 20

43kDa

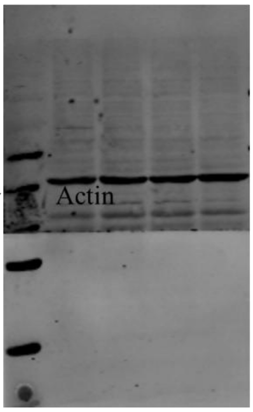

Nala（mM） 0 5 10 20

43kDa

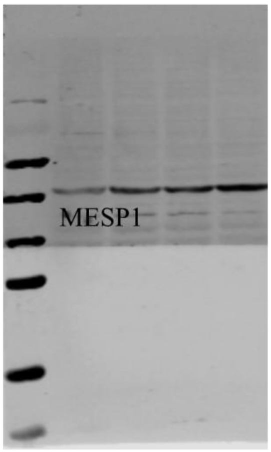

3、

Nala（mM） 0 5 10 20

43kDa

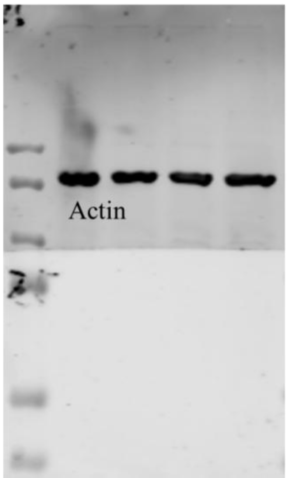

Nala（mM） 0 5 10 20

43kDa

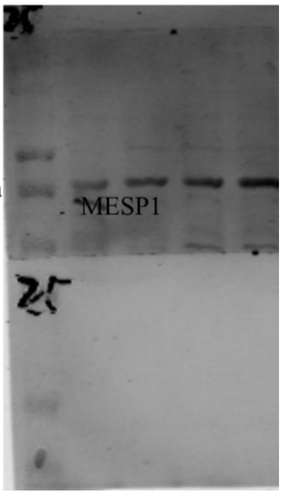

Figure4K: PANC-1

MESP1，n = 3

1、

DML（μM） 0 1 5 10 0 1 5 10

43kDa

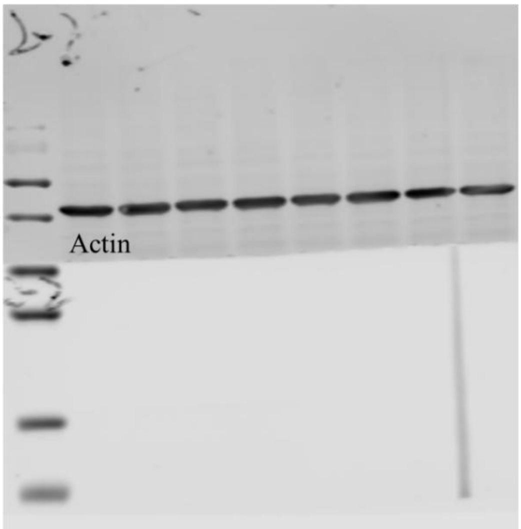

2、

DML（μM） 0 1 5 10 0 1 5 10

43kDa

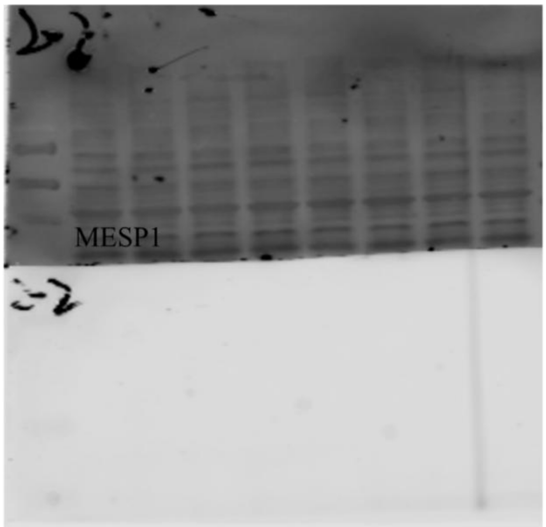

3、

DML（μM） 0 1 5 10

43kDa

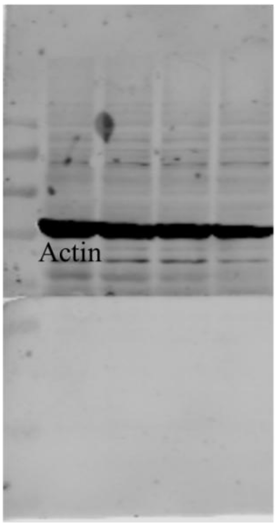

DML（μM） 0 1 5 10

43kDa

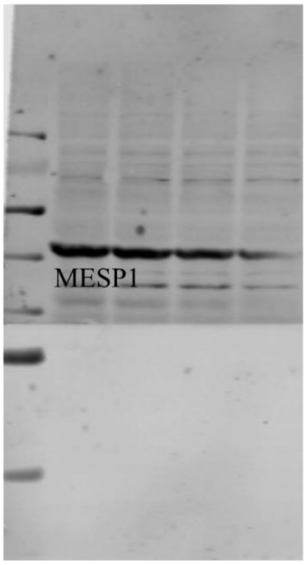

ASPC-1,MESP1，n = 3

1、 2、 3、

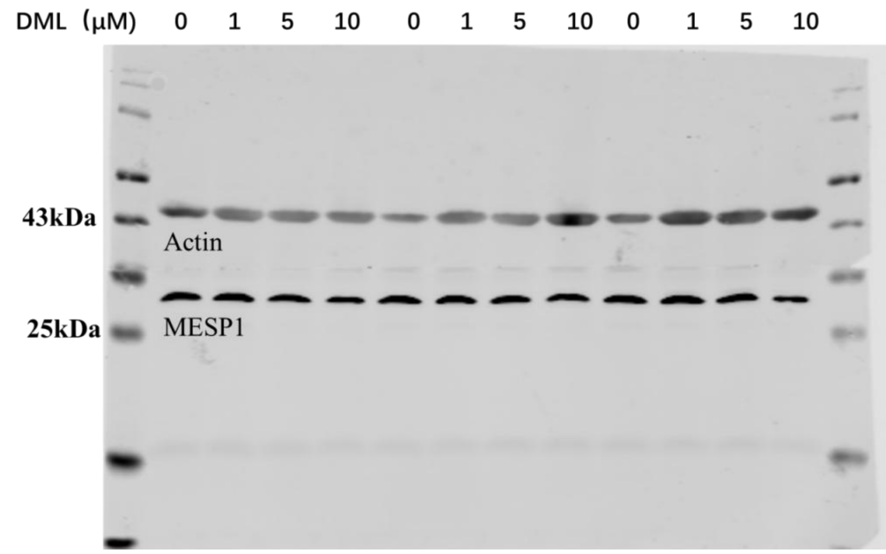

Figure4L:

MESP1，n = 3

1、 2、

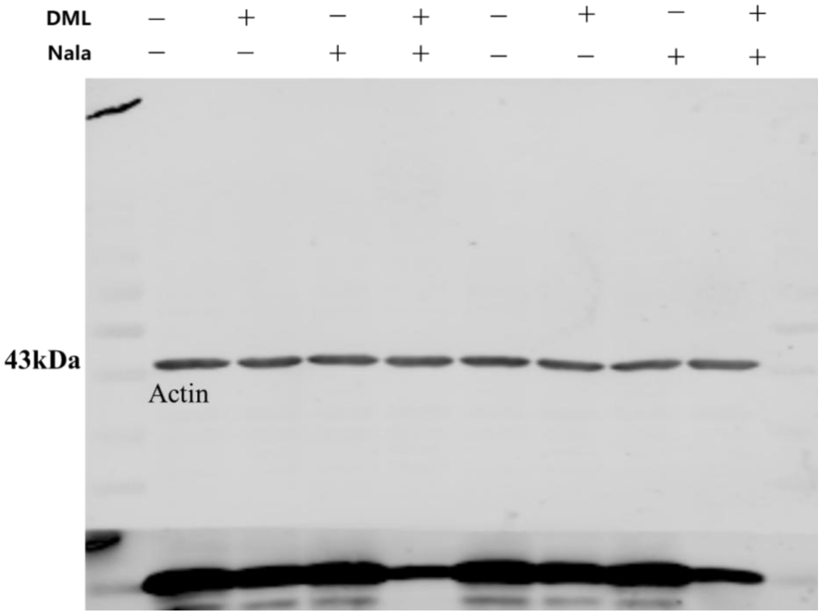

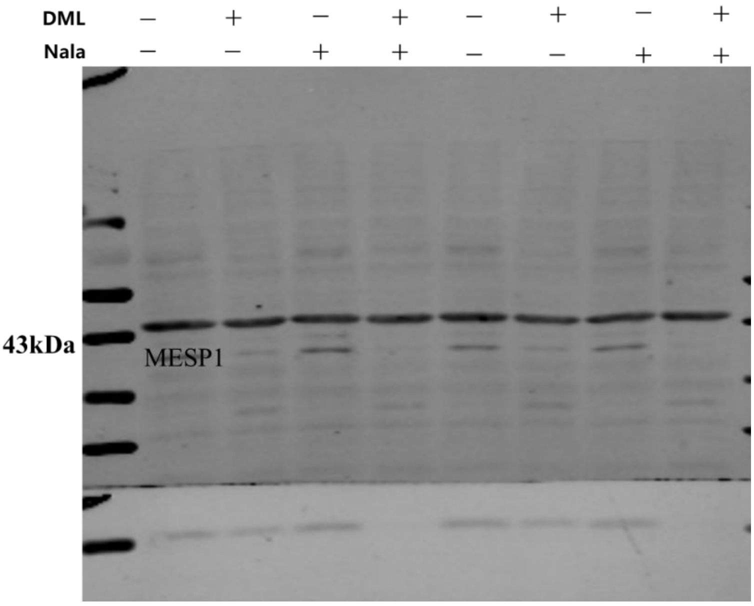

3、

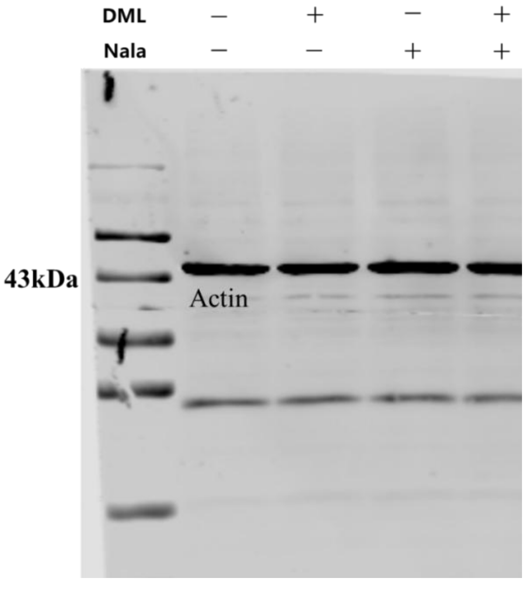

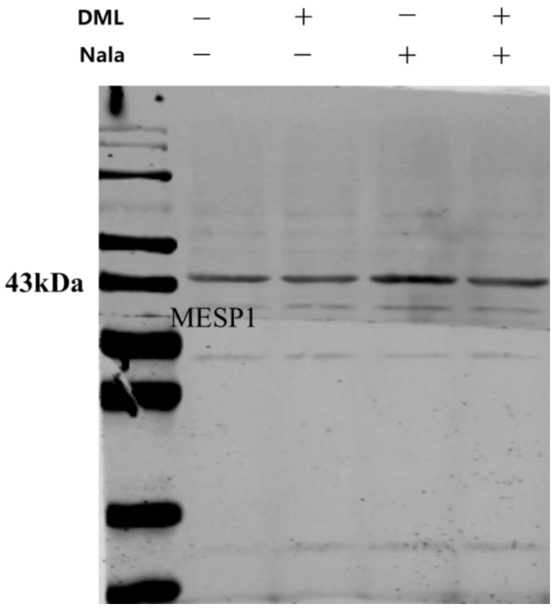

Figure4M:

MESP1，n = 3

1、

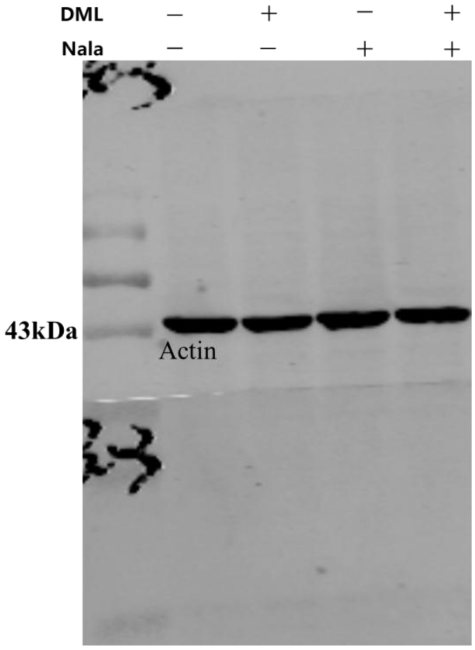

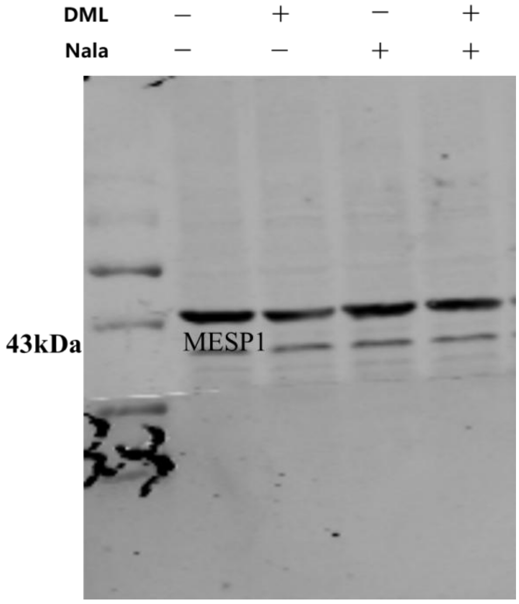

2、

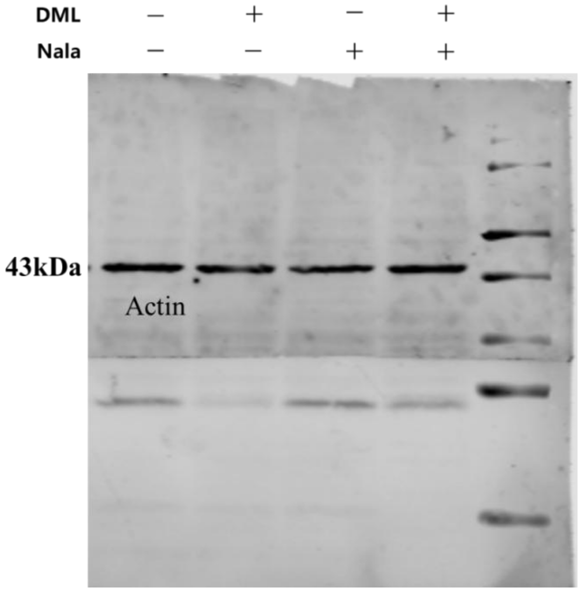

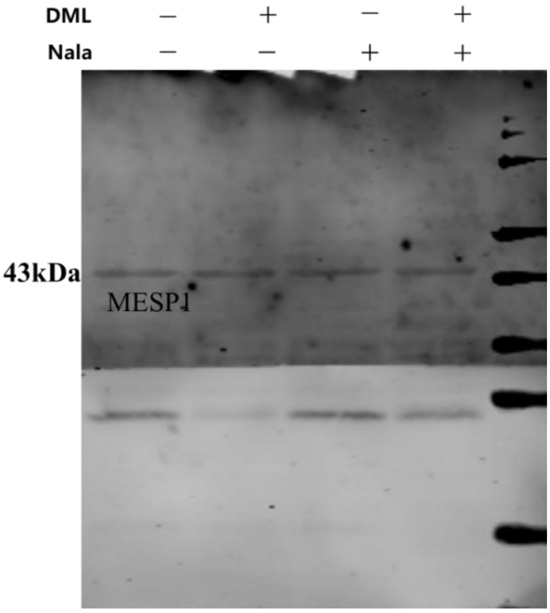

3、

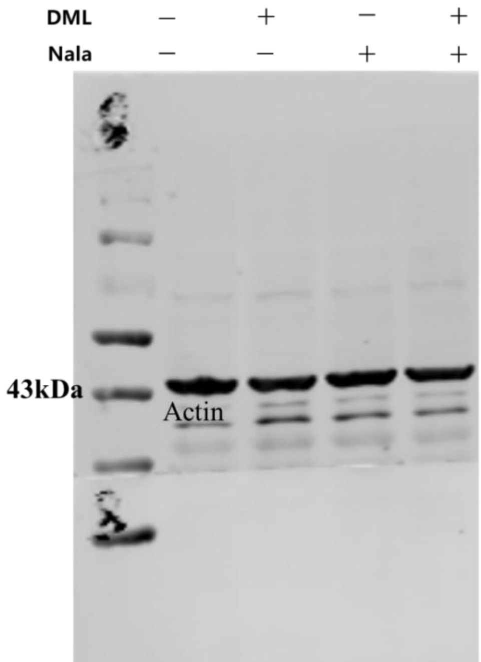

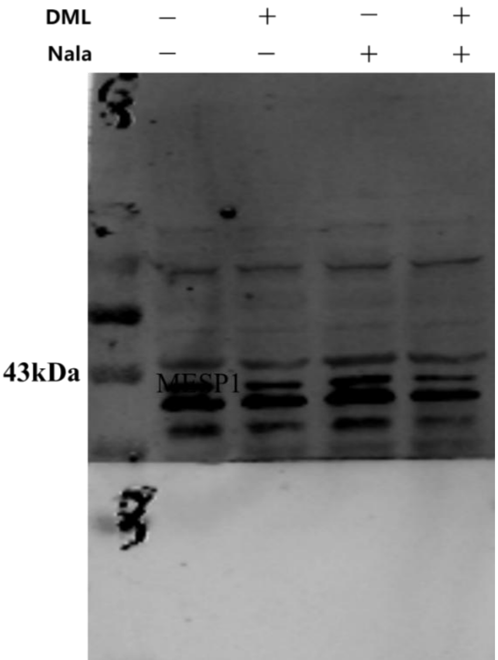

Figure5A:

MESP1，n = 6

1、

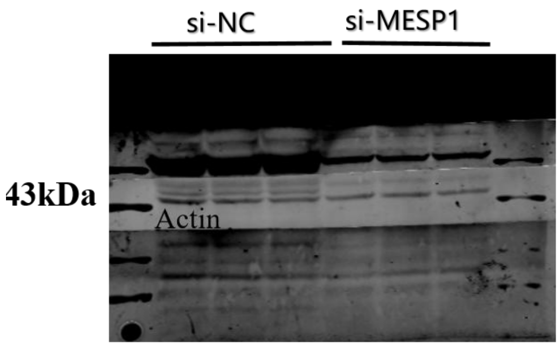

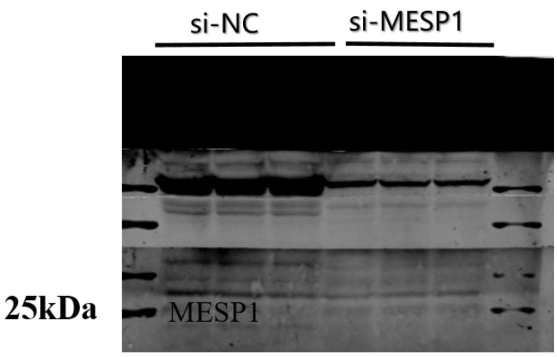

2、

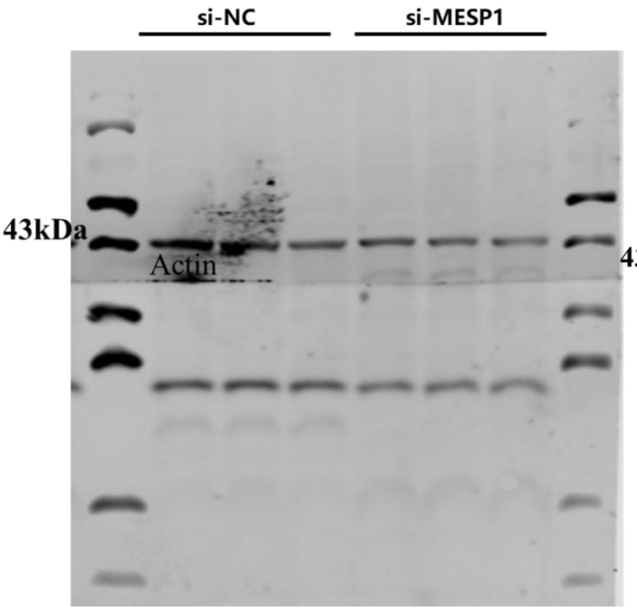

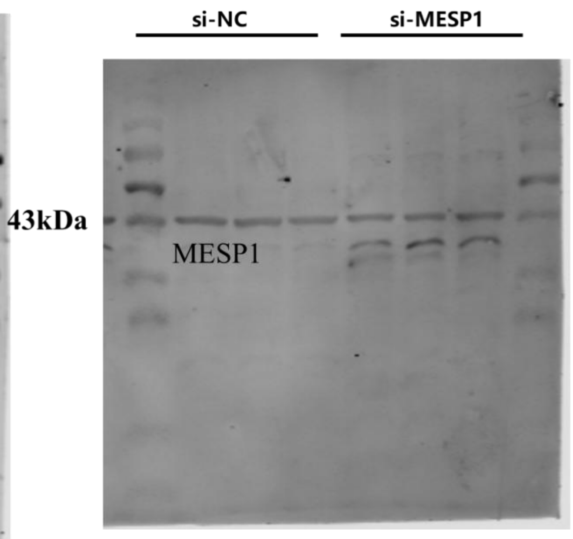

Bax，n = 6；Bcl2，n = 3

1、

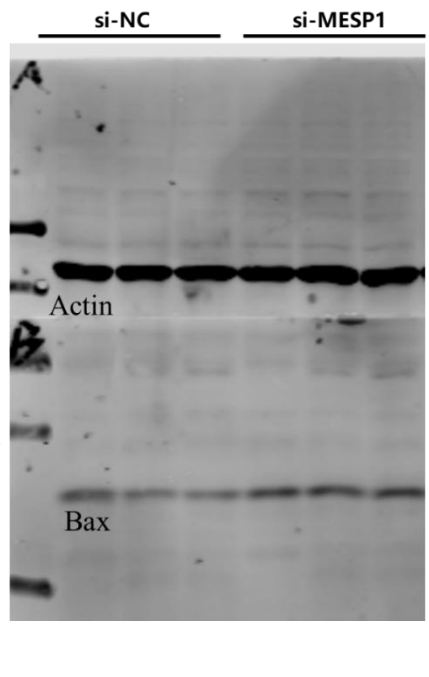

N-cadherin，

2、

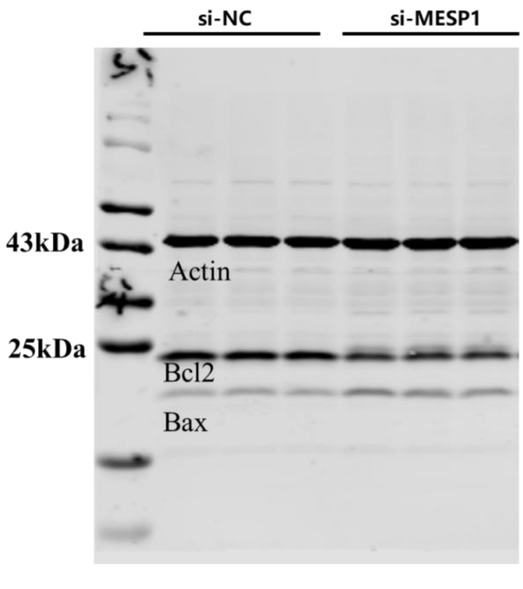

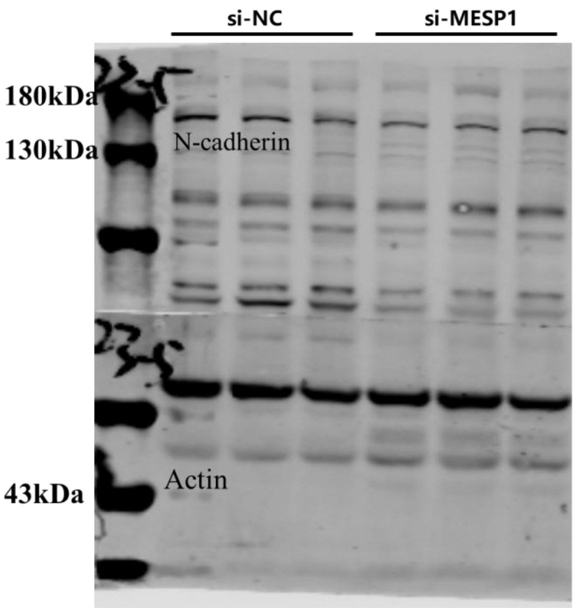

E-cadherin，n=6

1、

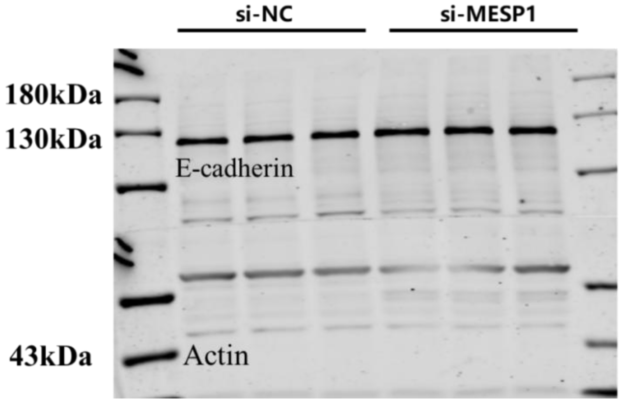

2、

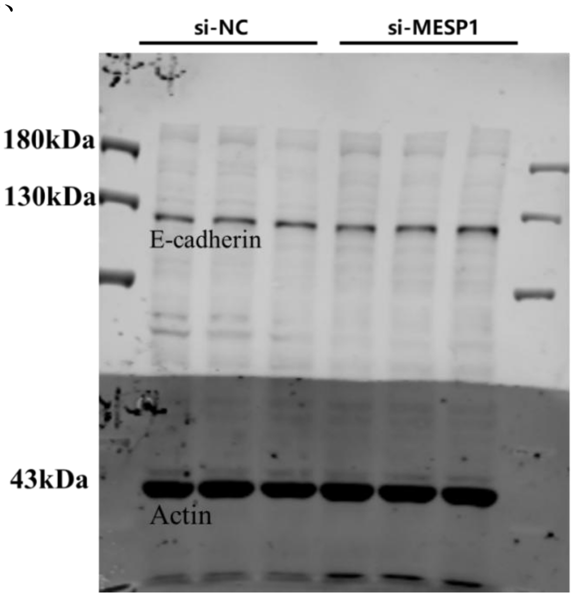

Vimentin，n=6

1、

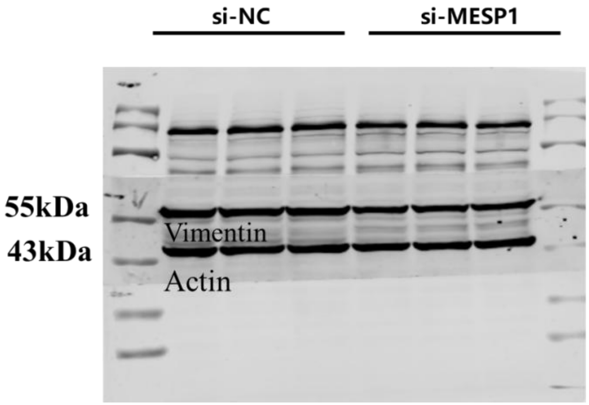

2、

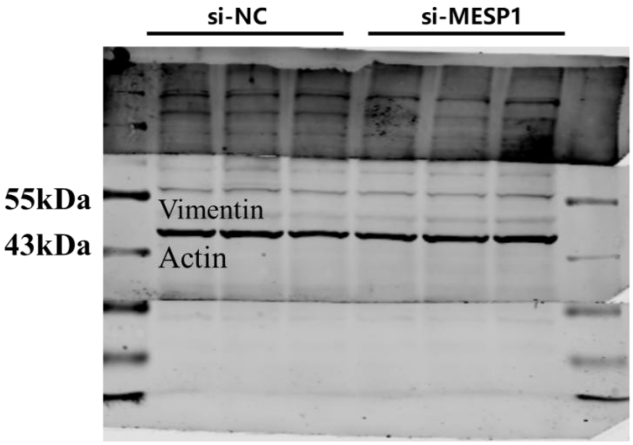

Figure5B:

MESP1，n = 3

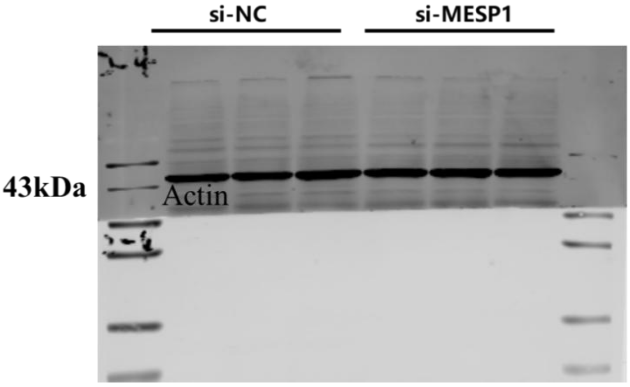

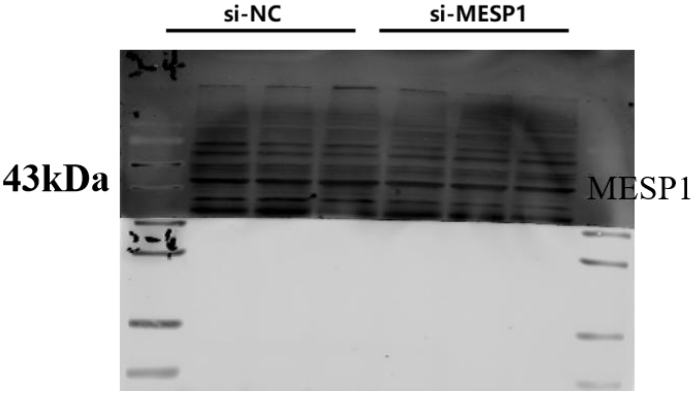

Bax，n = 3;Bcl2，n = 3

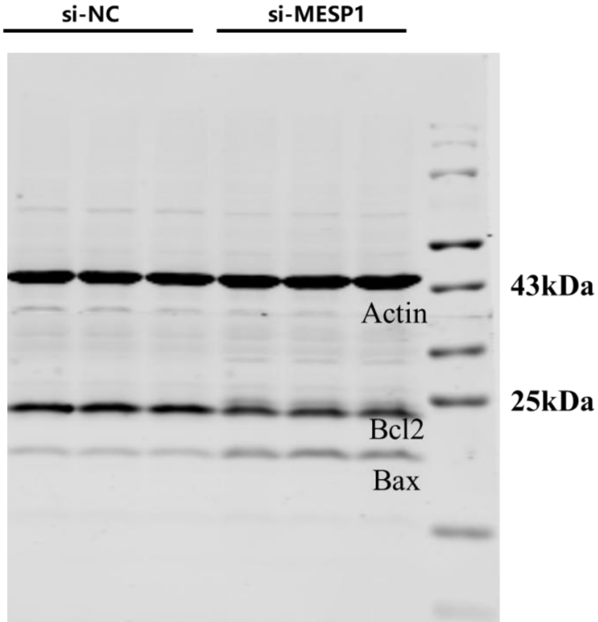

N-cadherin，n=3

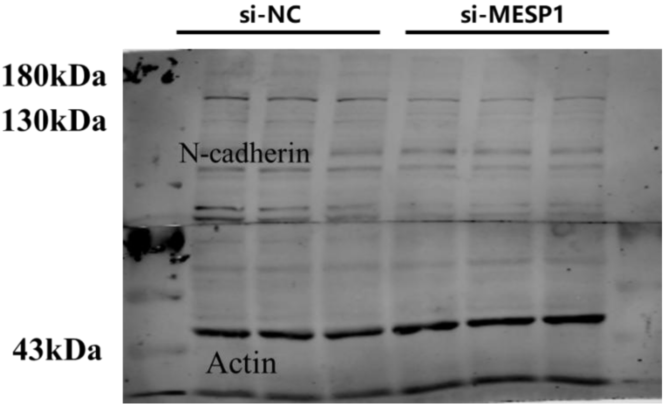

E-cadherin，n=3

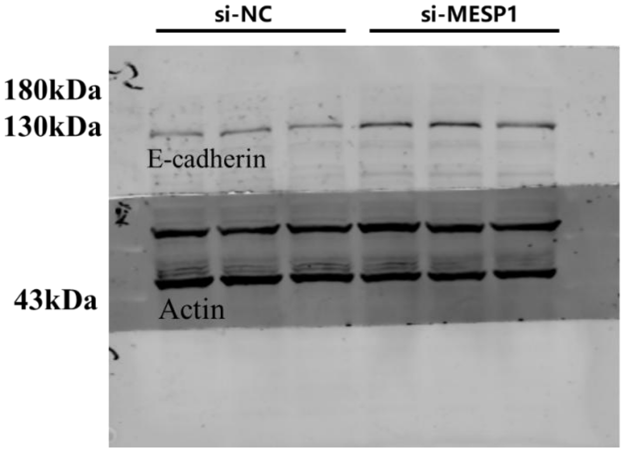

Vimentin，n=6

1、

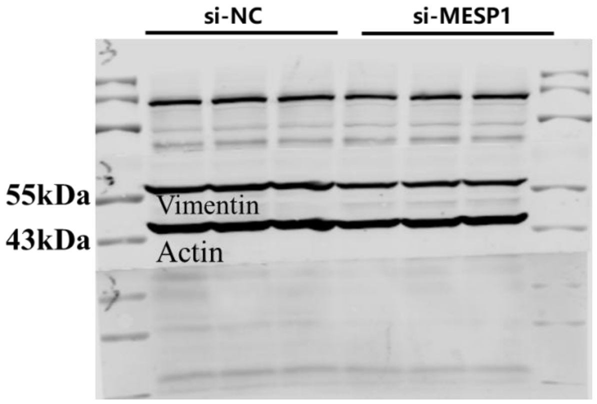

2、

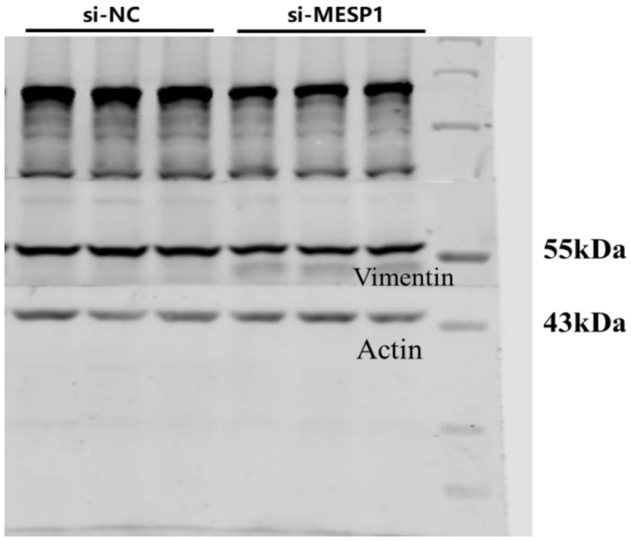

Figure6A:

MESP1，n = 3

1、 2、

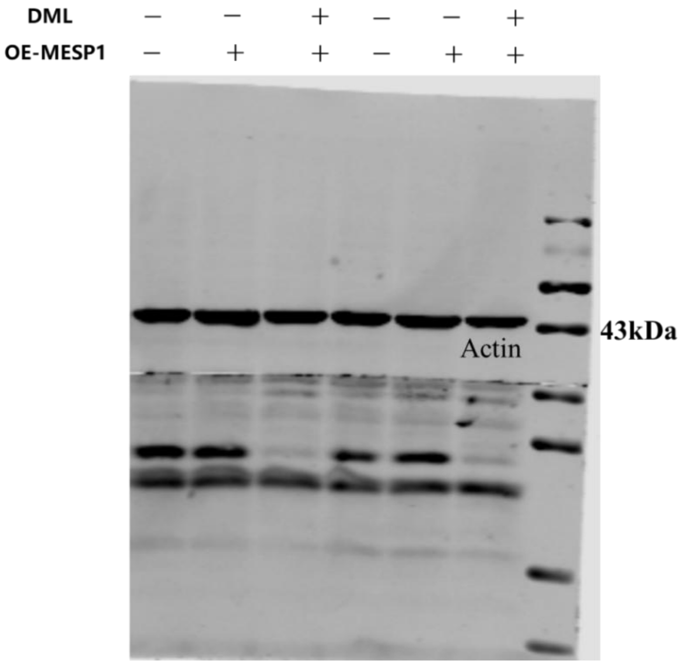

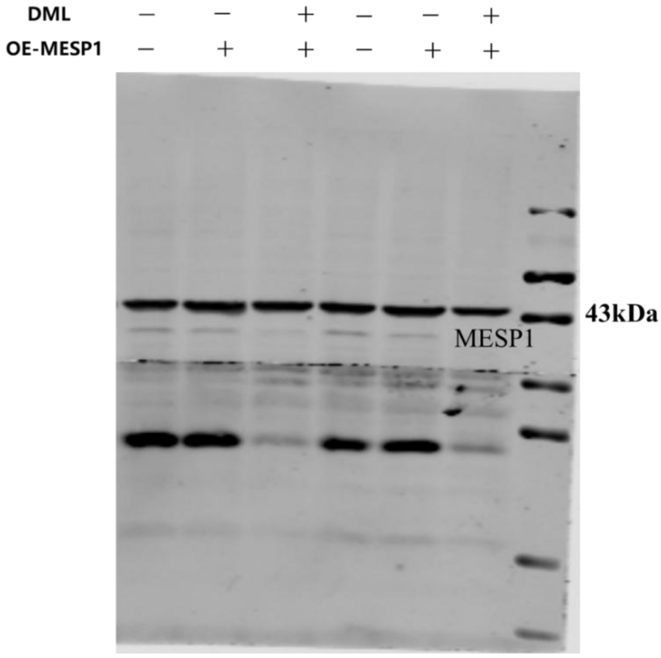

3、

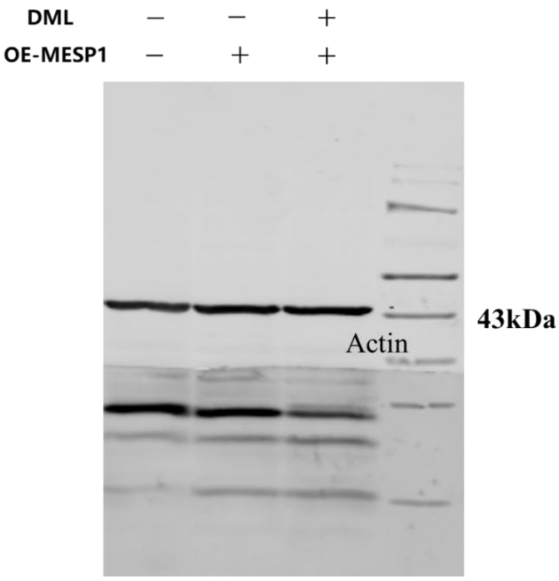

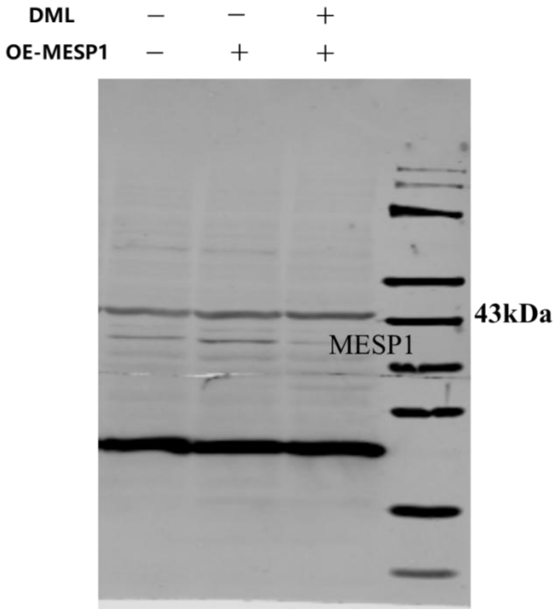

Bax，n=3

1、 2、 3、

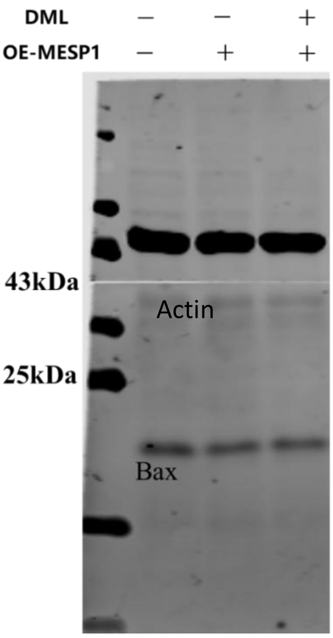

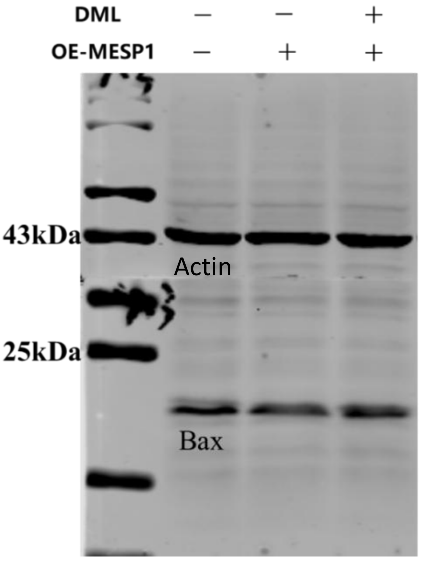

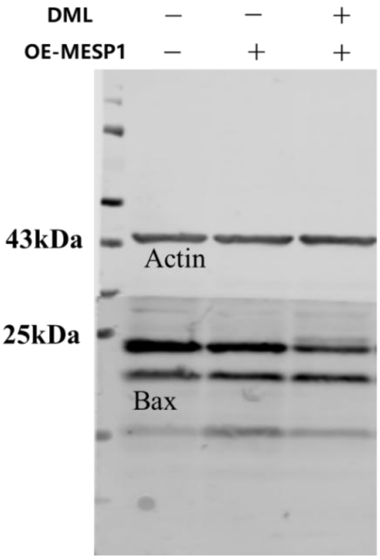

Bcl2，n=3

1、 2、 3、

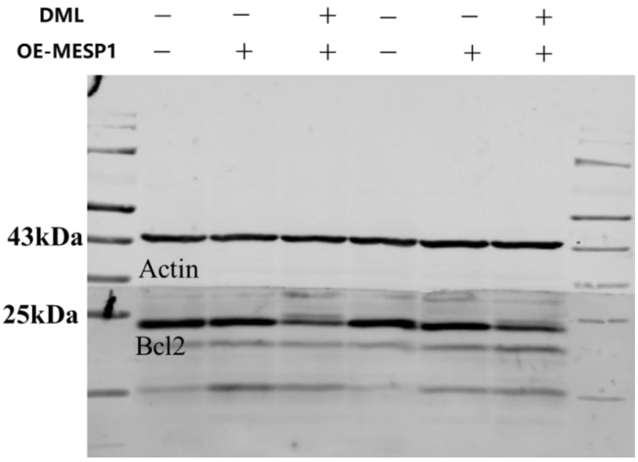

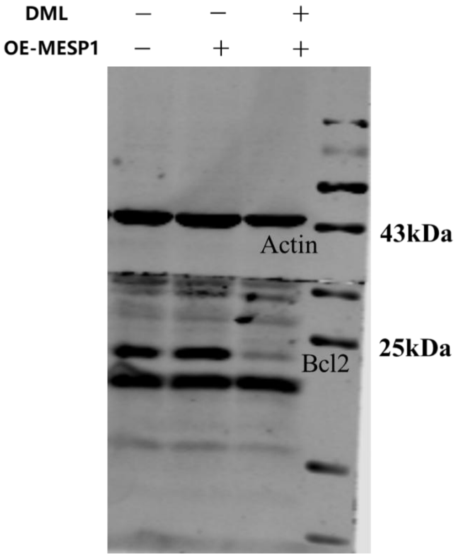

N-cadherin，n=3

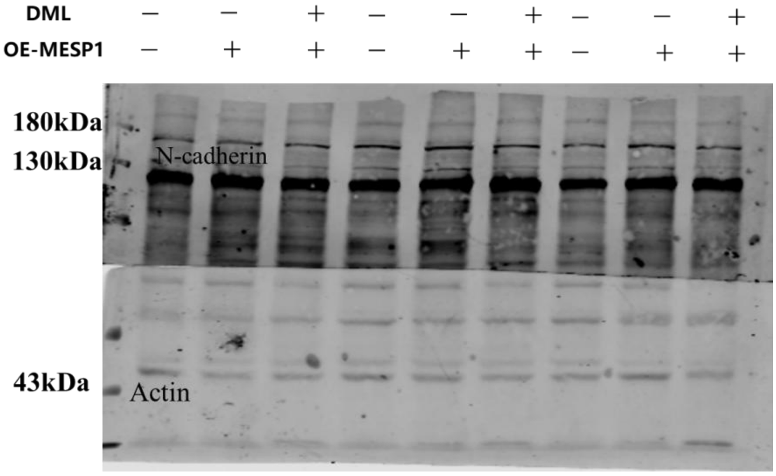

E-cadherin，n=4

1、

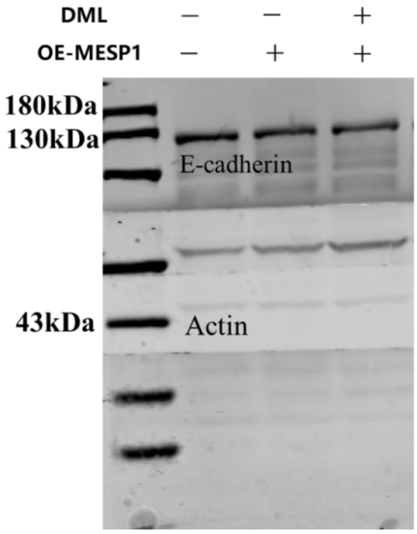

2、

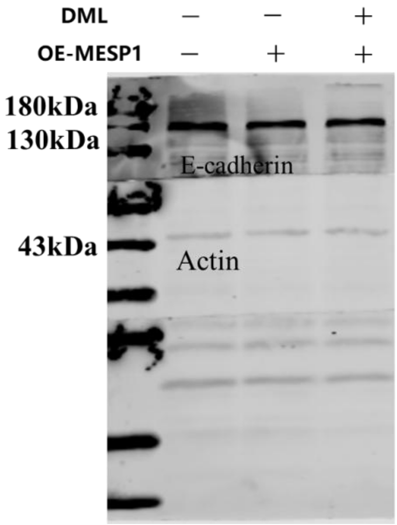

3、

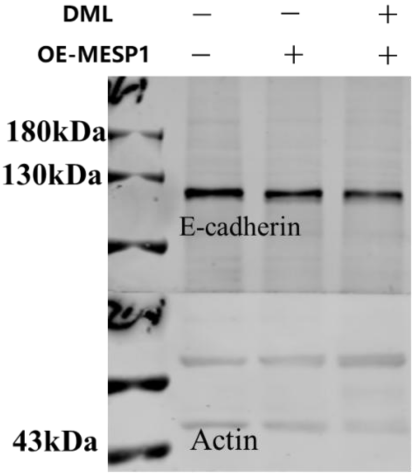

4、

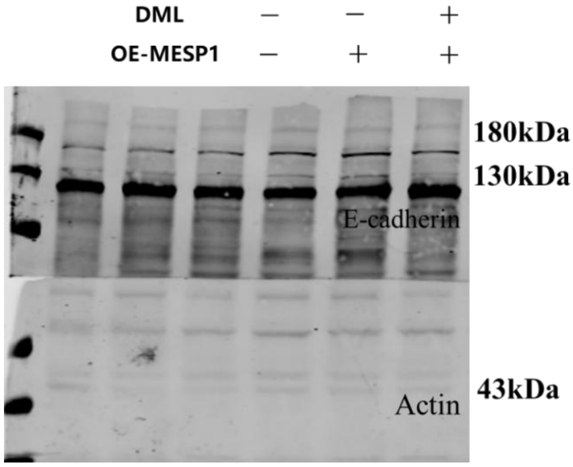

Vimentin，n=4

1、 2、

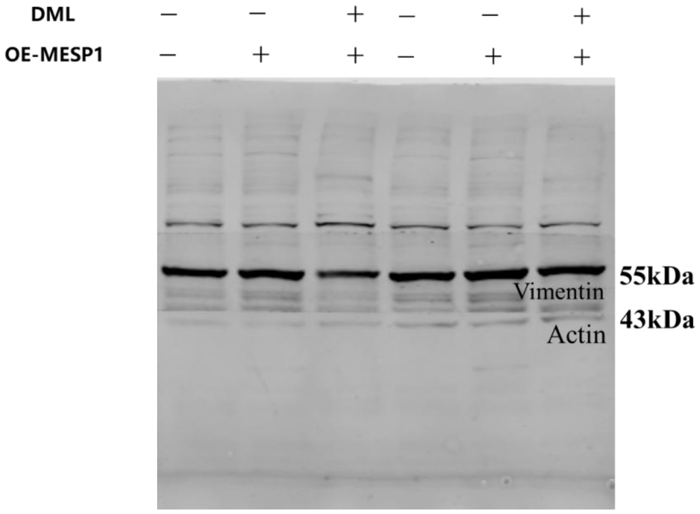

3、 4、

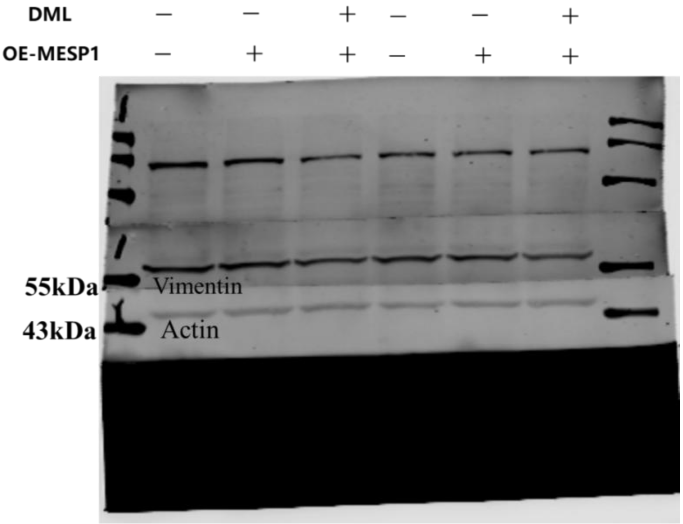

Figure6B:

MESP1，n = 3

1、 2、

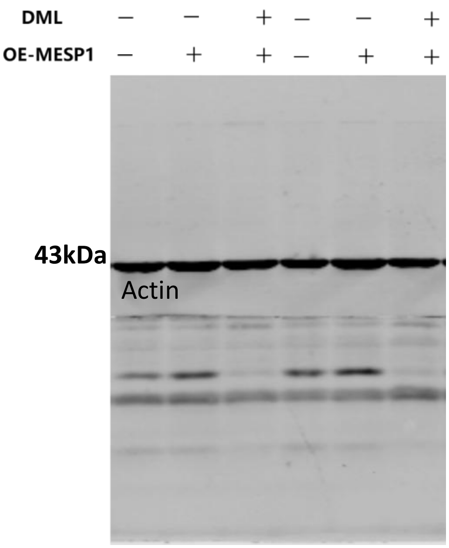

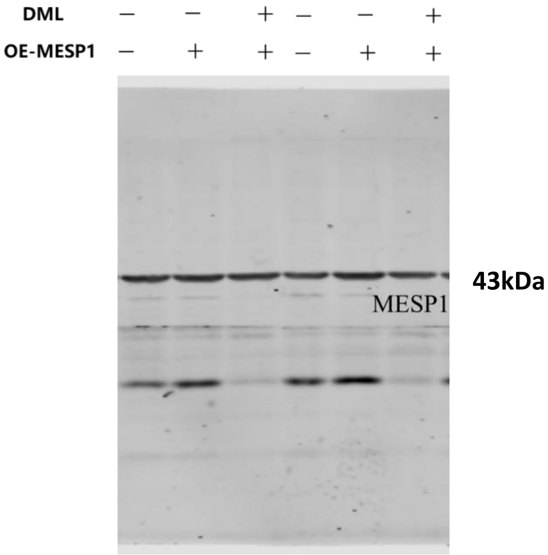

3、

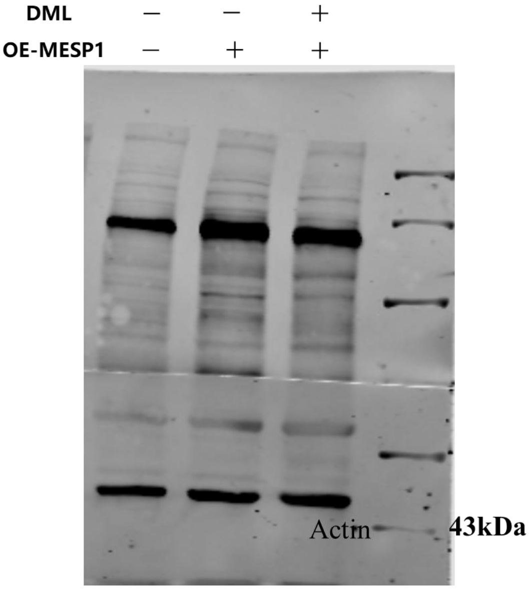

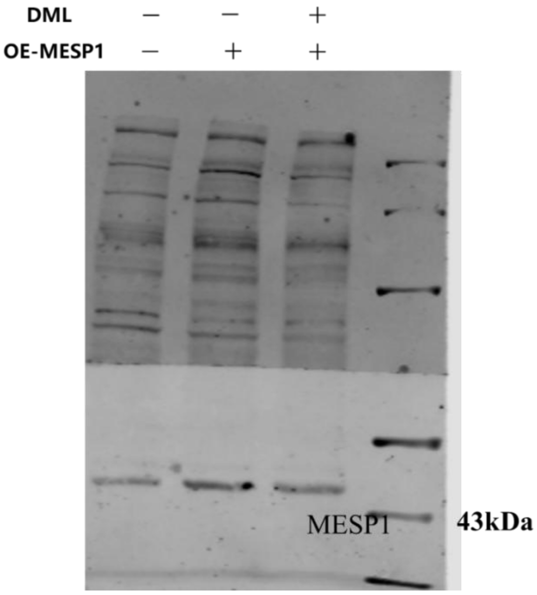

Bax，n=3

1、 2、 3、

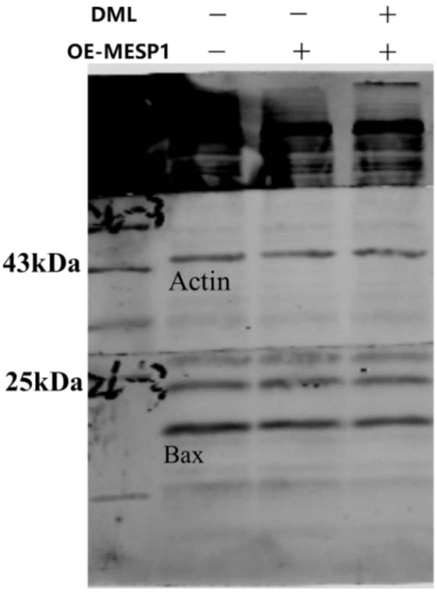

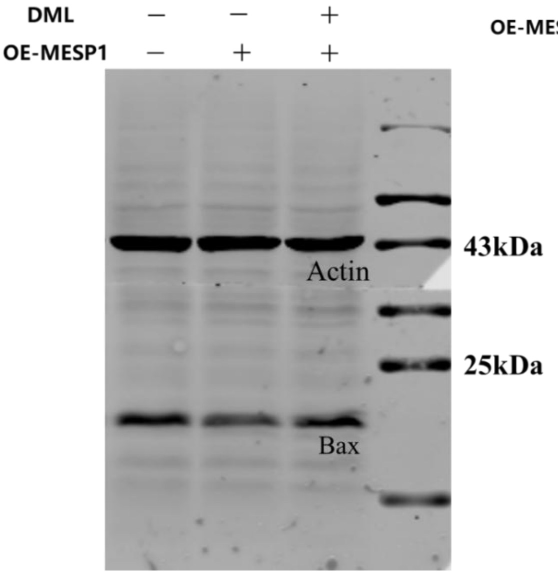

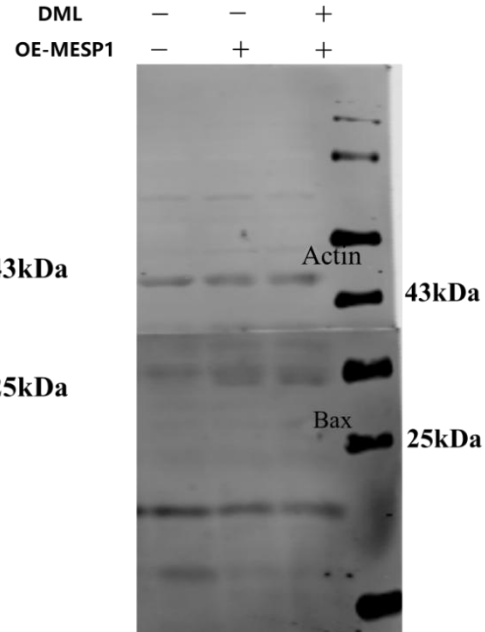

Bcl2，n=3

1、

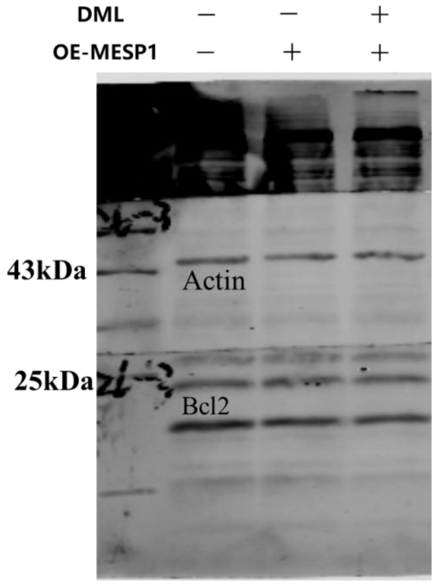

2、 3、

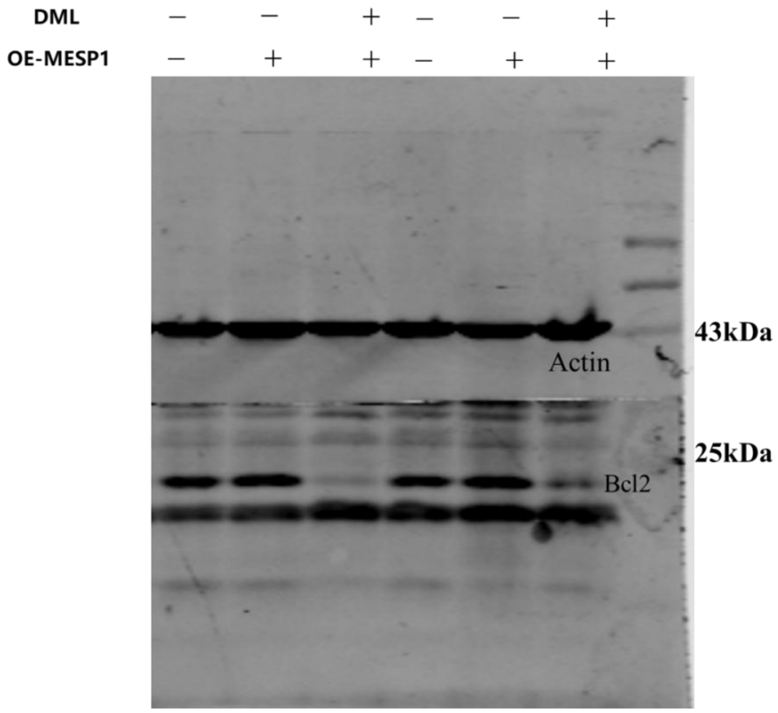

N-cadherin，n=4

1、 2、

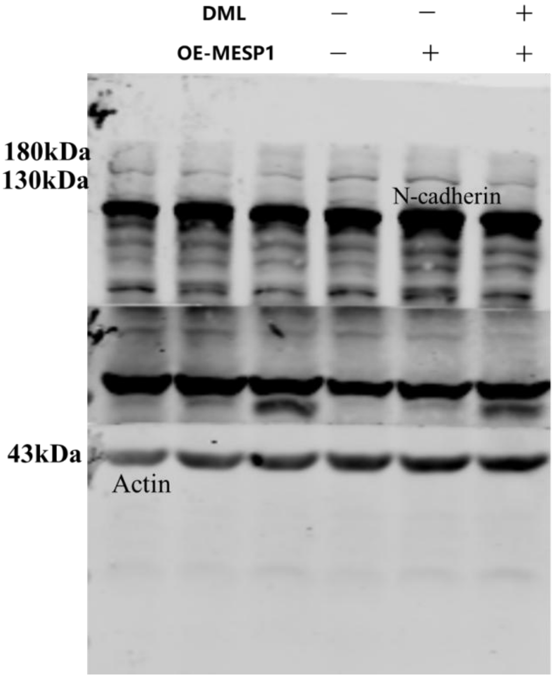

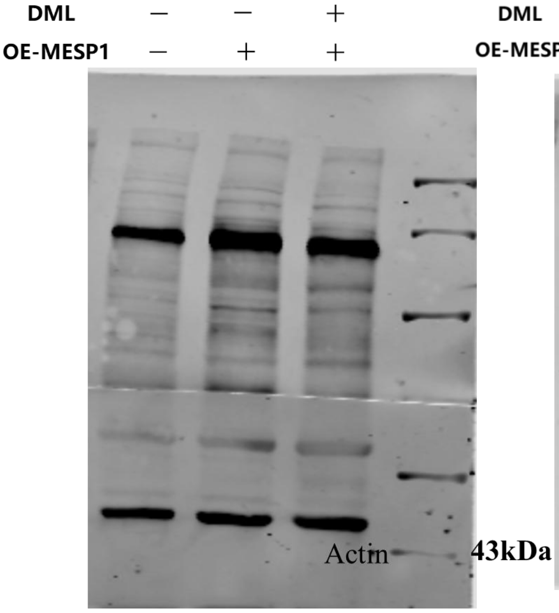

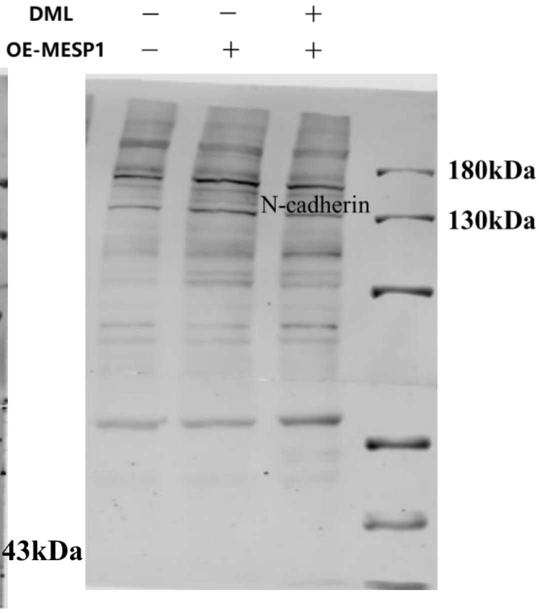

3、 4、

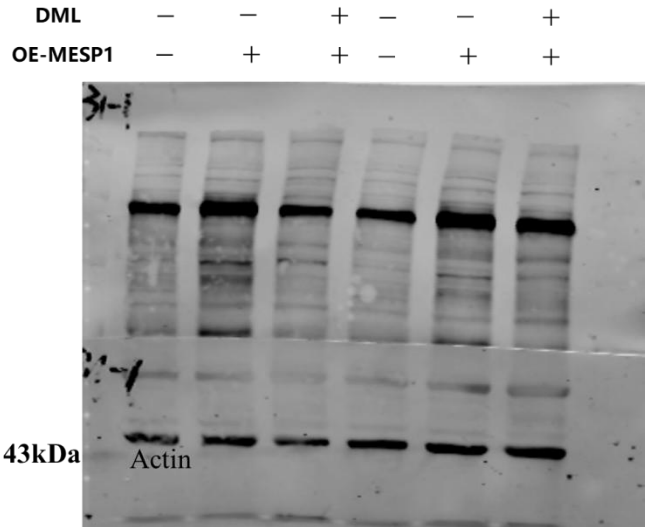

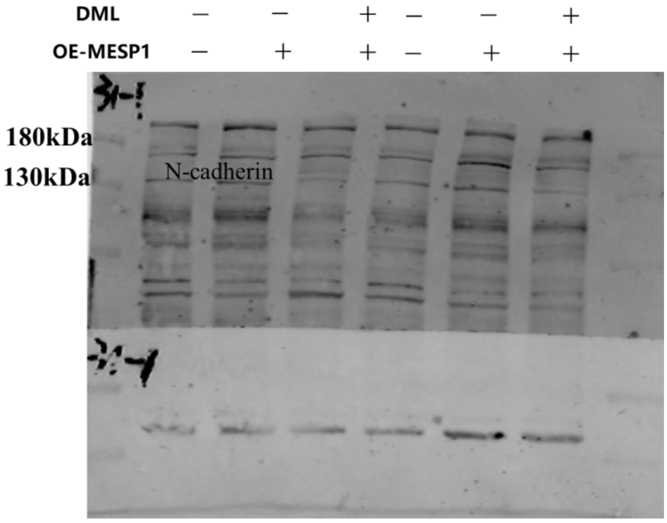

E-cadherin，n=3

1、

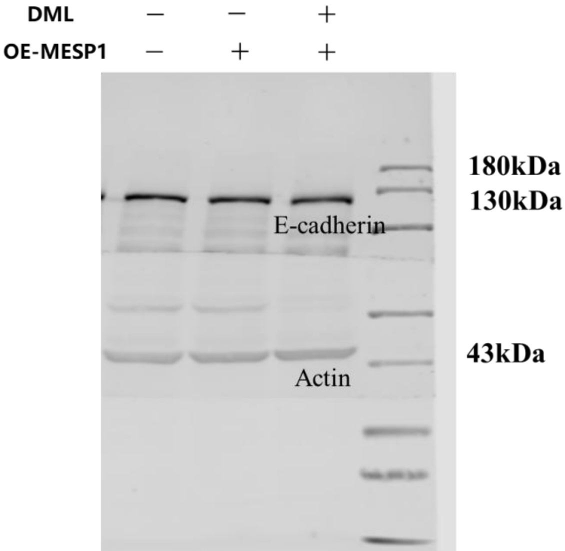

2、

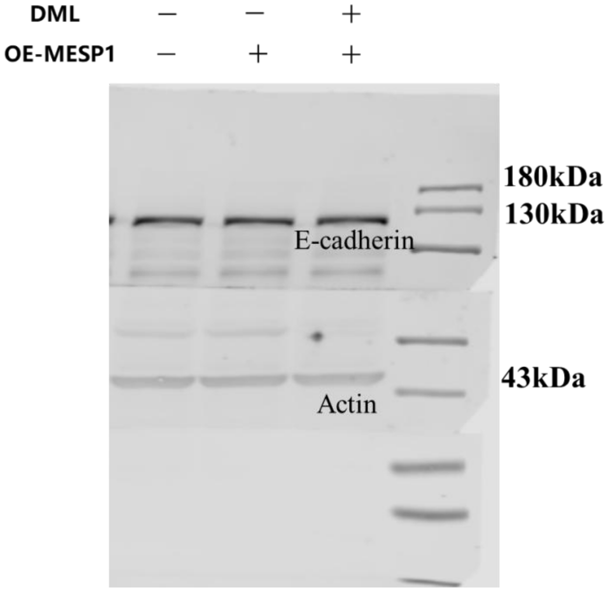

3、

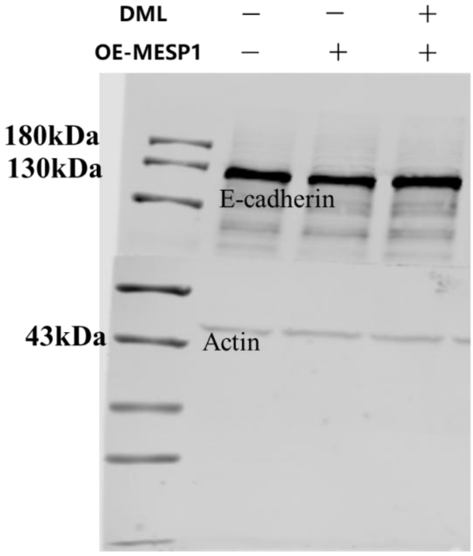

Vimentin，n=3

1、

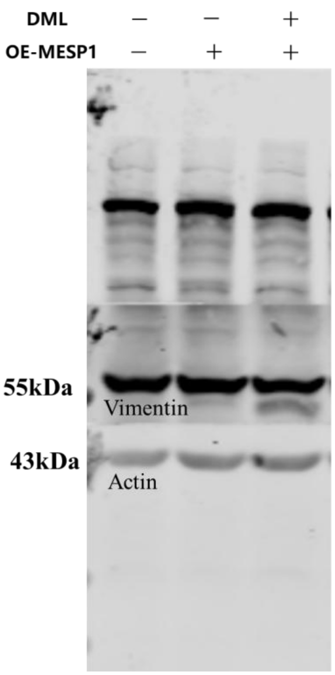

2、

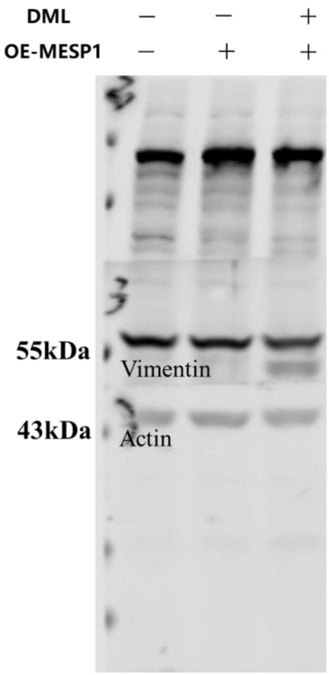

3、

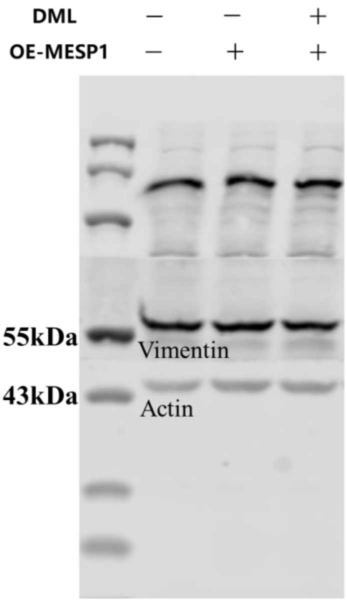

Supplement: Supplementary file 1 — Original western blots [file 41420_2025_2603_MOESM1_ESM.pdf]
