# Supplementary material for: Demethylzeylasteral suppresses the expression of MESP1 by reducing H3K18la level to inhibit the malignant behaviors of pancreatic cancer
Source: Cell Death Discov. 2025 Jul 3;11:305. doi: 10.1038/s41420-025-02603-9 (PMC12229608; doi:10.1038/s41420-025-02603-9)
Supplement: Supplementary file 2 — Supplementary results [file 41420_2025_2603_MOESM2_ESM.docx]

**
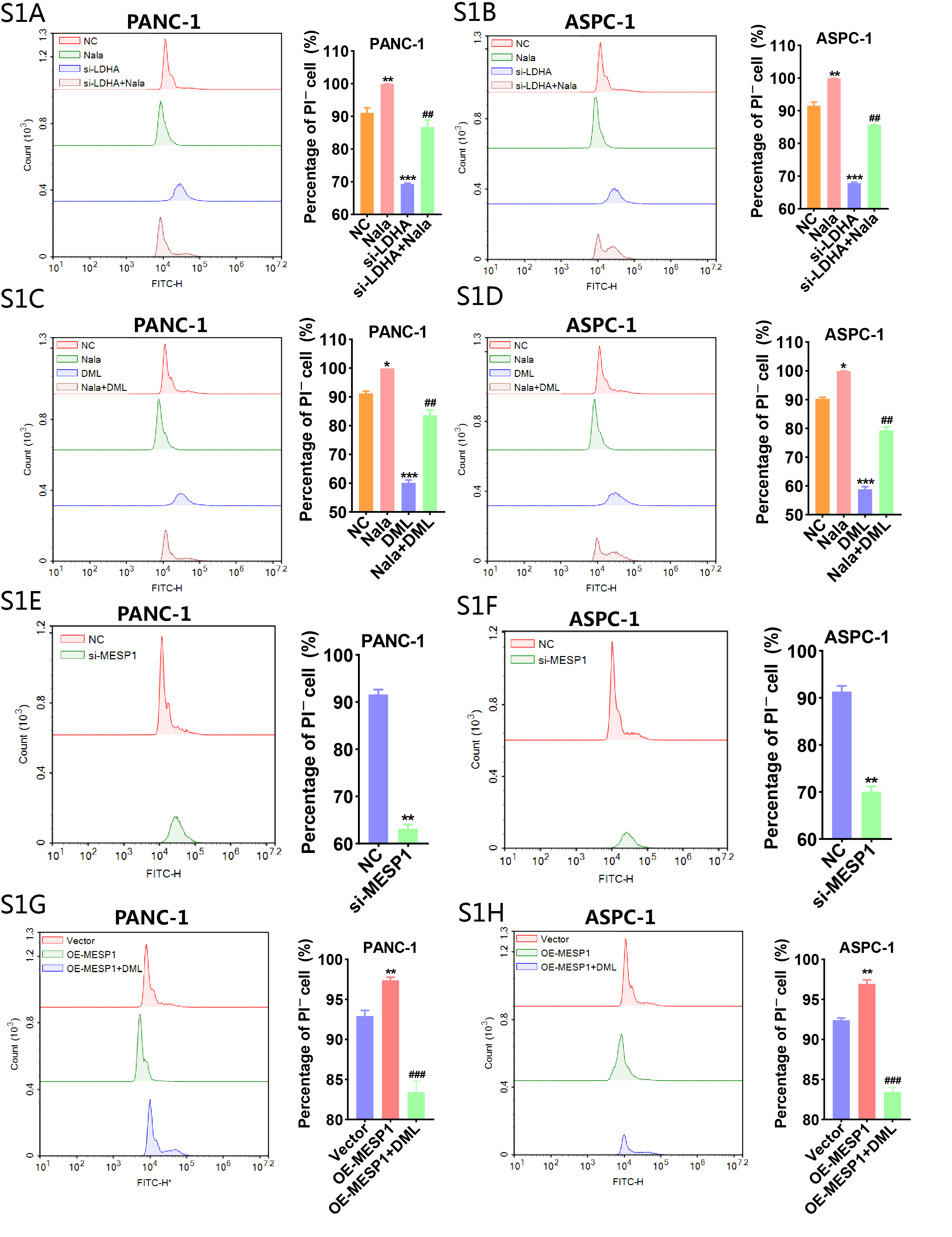
**

**Supplementary Fig. 1 Cellular apoptosis rates were determined via flow cytometry, with propidium iodide (PI)-positive cells identified as apoptotic.** All data are shown as the mean ± SD of at least three independent experiments. **p* < 0.05, ***p* < 0.01, ****p* < 0.001, *vs*. NC or Vector. ^##^*p* < 0.01, vs. DML or OE-MESP1.

**
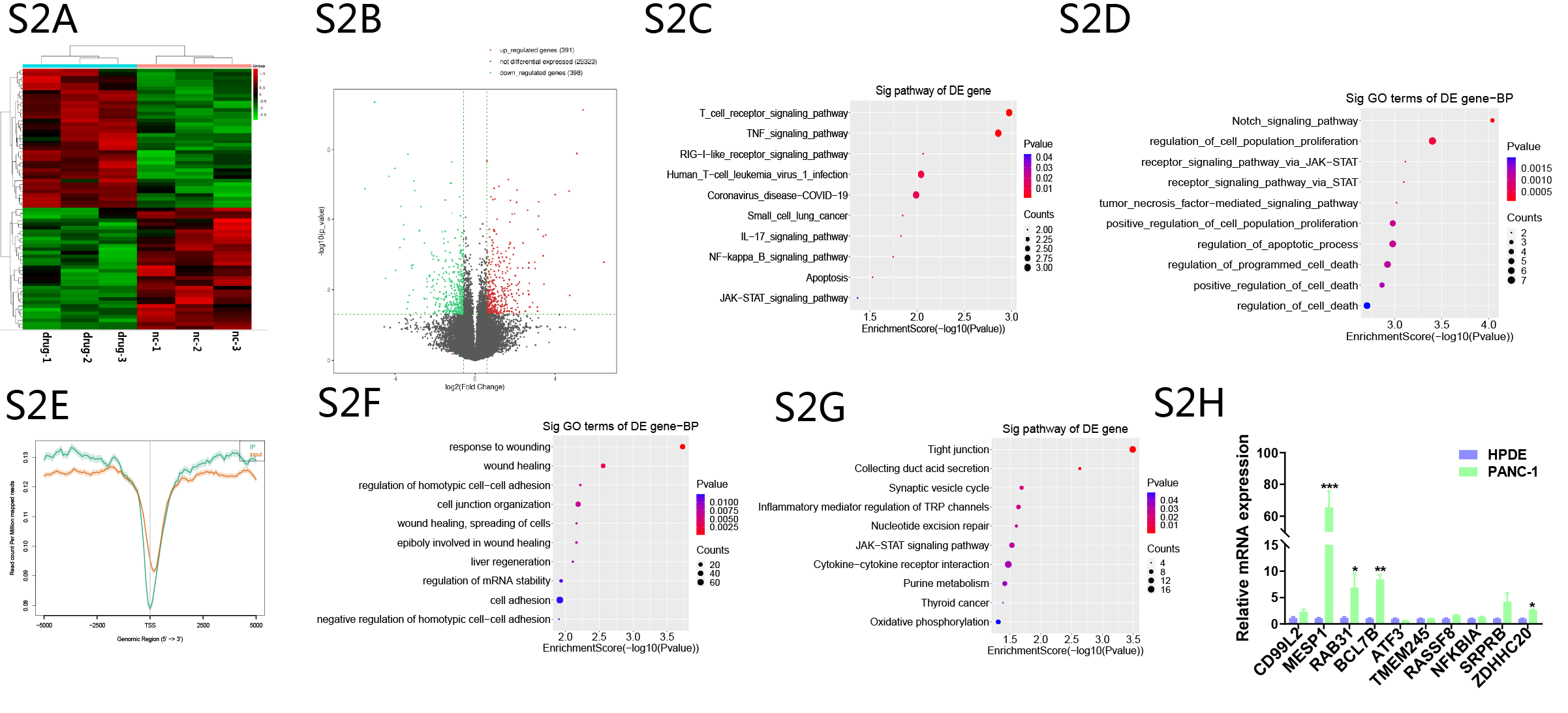
**

**Supplementary Fig. 2 KEGG and GO analyzed RNA-Seq of DML and ChIP-Seq of H3K18la. A** The heatmap of differential genes obtained after DML treatment of PANC-1 cells. **B** Volcano plot of differentially expressed genes, the -log10 (p-value) and the log2 (fold change) are plotted on the y and x axes, respectively**. C, D** KEGG and GO analyses were performed on the differentially expressed genes caused by DML RNA-seq. **E** Distribution of H3K18la sites relative to translation start site (TSS). **F, G** KEGG and GO analyses were performed on the genes bound to H3K18la. **H** The mRNA levels of candidate genes were verified by qRT-PCR in PANC-1 and HPDE. All data are shown as the mean ± SD of at least three independent experiments. **p* < 0.05, ***p* < 0.01, ****p* < 0.001, *vs*. HPDE.


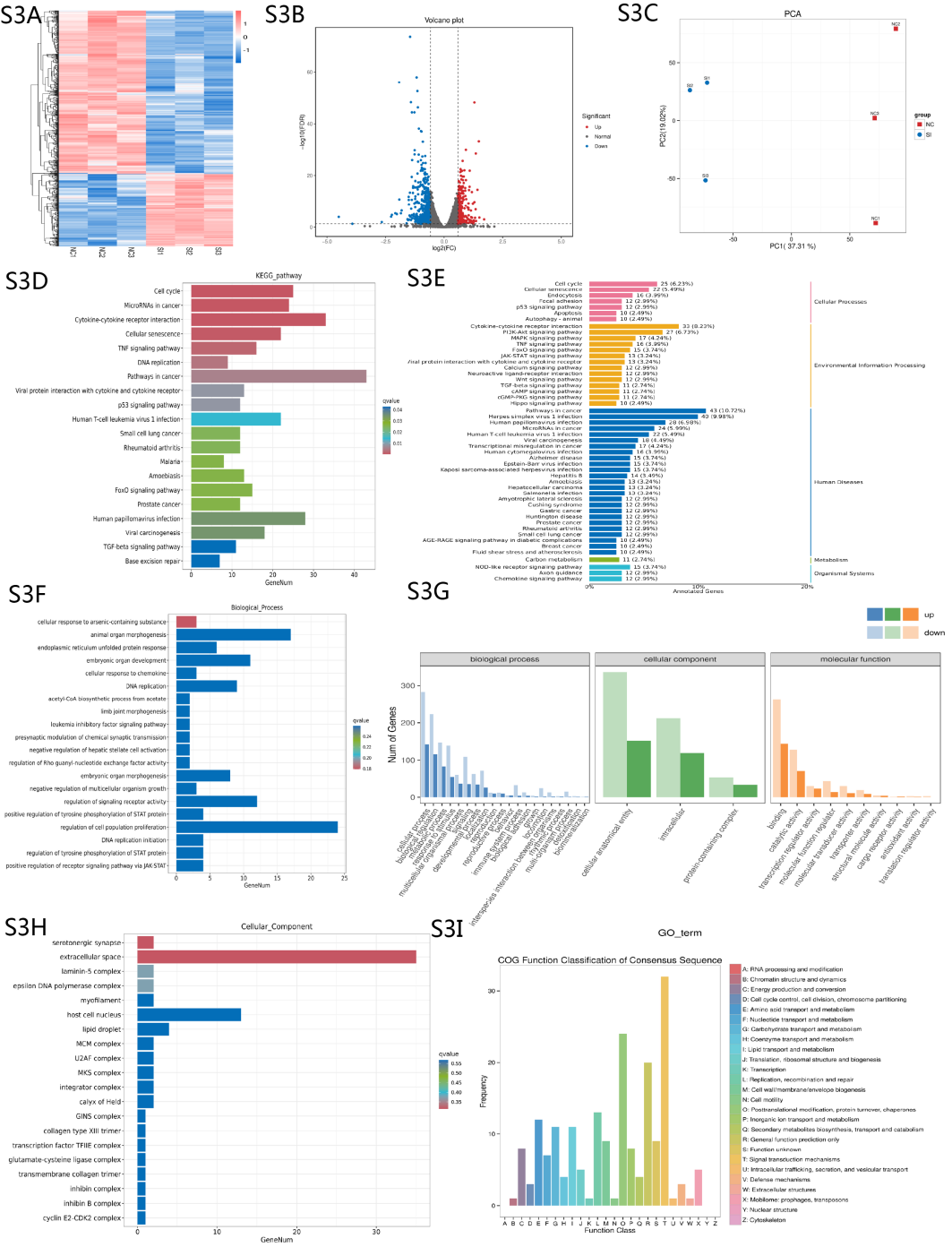


**Supplementary Fig. 3** **The potential cancer**-**promoting mechanism of MESP1 was analyzed. A** The heatmap of differential genes obtained after silencing MESP1 of PANC-1 cells. **B** Volcano plot of differentially expressed genes, the -log10 (p-value) and the log2 (fold change) are plotted on the y and x axes, respectively**. C** Principal components analysis of all genes of si-NC and si-MESP1. **D, E** KEGG analysis of differentially expressed genes was performed (D), and these pathways were classified (E). **F** Biological process of differentially expressed genes. **G** Gene ontology classification analysis to study the biological functions of differentially expressed genes. **H, I** Cellular component analysis (H) and clusters of orthologous groups classification analysis (I) were uesd to analyze the functions of differentially expressed genes comprehensively.
